# StayGold variants for molecular fusion and membrane-targeting applications

**DOI:** 10.1038/s41592-023-02085-6

**Published:** 2023-11-30

**Authors:** Ryoko Ando, Satoshi Shimozono, Hideo Ago, Masatoshi Takagi, Mayu Sugiyama, Hiroshi Kurokawa, Masahiko Hirano, Yusuke Niino, Go Ueno, Fumiyoshi Ishidate, Takahiro Fujiwara, Yasushi Okada, Masaki Yamamoto, Atsushi Miyawaki

**Affiliations:** 1 Laboratory for Cell Function Dynamics, RIKEN Center for Brain Science, Wako-city, Japan; 2 Biotechnological Optics Research Team, RIKEN Center for Advanced Photonics, Wako-city, Japan; 3 Department of Optical Biomedical Science, Institute for Life and Medical Sciences, Kyoto University, Kyoto, Japan; 4 RIKEN SPring-8 Center, 1-1-1 Kouto, Sayo-cho, Sayo-gun, Japan; 5 Cellular Dynamics Laboratory, RIKEN Cluster for Pioneering Research (CPR), Saitama, Japan; 6 Institute for Integrated Cell-Material Sciences (WPI-iCeMS), Kyoto University, Sakyo-ku, Kyoto, Japan; 7 Laboratory for Cell Polarity Regulation, RIKEN Center for Biosystems Dynamics Research, Suita, Japan; 8 Department of Cell Biology, Department of Physics, UBI and WPI-IRCN, The University of Tokyo, Bunkyo-ku, Tokyo, Japan; 9 Laboratory of Bioresponse Analysis, Institute for Life and Medical Sciences, Kyoto University, Kyoto, Japan

**Keywords:** Fluorescence imaging, Fluorescent proteins, Super-resolution microscopy, Wide-field fluorescence microscopy

## Abstract

Although StayGold is a bright and highly photostable fluorescent protein, its propensity for obligate dimer formation may hinder applications in molecular fusion and membrane targeting. To attain monovalent as well as bright and photostable labeling, we engineered tandem dimers of StayGold to promote dispersibility. On the basis of the crystal structure of this fluorescent protein, we disrupted the dimerization to generate a monomeric variant that offers improved photostability and brightness compared to StayGold. We applied the new monovalent StayGold tools to live-cell imaging experiments using spinning-disk laser-scanning confocal microscopy or structured illumination microscopy. We achieved cell-wide, high-spatiotemporal resolution and sustained imaging of dynamic subcellular events, including the targeting of endogenous condensin I to mitotic chromosomes, the movement of the Golgi apparatus and its membranous derivatives along microtubule networks, the distribution of cortical filamentous actin and the remolding of cristae membranes within mobile mitochondria.

## Abstract (web summary)

Monomeric and tandem dimer derivatives of the bright and photostable green fluorescent protein StayGold offer versatile tools for tagging target proteins and membranes in extended live-cell imaging.

## Main

In live fluorescence imaging, an increase in illumination intensity (irradiance) results in an increase in brightness (photon budget), thereby improving the spatiotemporal resolution of an observation; however, elevated irradiance naturally leads to photobleaching of fluorescent dyes and/or photodamage of observed cells. If the photobleaching problem could be solved, how would the spatial and temporal scales of fluorescence imaging be extended? In our previous study, we attempted to answer this question by developing a bright and highly photostable fluorescent protein (FP) that we provocatively named StayGold and demonstrating its usefulness by imaging a cell’s endoplasmic reticulum (ER) and mitochondria with an enhanced spatiotemporal resolution and over an extended observation period^1^. Being a dimer, this FP was expressed as luminal soluble markers in these organelles. In the present study, to further extend the applications of this FP to include attachment to membranes or naturally oligomeric proteins, we endeavored to develop techniques that enable monovalent tagging with StayGold.

We first used a compromised but powerful approach to generate tandem dimer (td) constructs^2^ of StayGold with various adaptors and flexible linkers. Although this approach can, in principle, achieve monovalent tagging, the original td construct (tdStayGold)^1^ gave only dim labeling and some other td constructs were found to exhibit cohesive tendencies under physiological conditions by our fluorescence-based technology detecting protein–protein interaction (Fluoppi) assay^3^ and the organized smooth ER (OSER) assay^4–6^. Therefore, we attempted to create td variants that were practically bright and dispersive.

Our second approach used directed evolution to drive the monomerization of StayGold. Although we have been refining the crystal structure of StayGold to elucidate the molecular mechanism responsible for its outstanding photostability, we here characterized the dimeric structure and, on the basis of the information, introduced mutations into the dimer interface to disrupt the dimerization. Although breaking the dimer interface of StayGold without losing its high photostability and brightness was challenging, we eventually produced practically useful monomeric variants that exhibited excellent dispersibility and retained the high photostability and brightness of StayGold. In addition, we found that their brightness and photostability were further improved in some respects compared to the original FP.

In our previous study, to achieve high-speed super-resolution or volumetric imaging for extended periods, we used structured illumination microscopy (SIM)^7^ and spinning-disk super-resolution microscopy (SDSRM)^8^, both of which can demonstrate the best features of StayGold^1^. In the present study, continuous sustainable observation of molecules and membranes labeled with the new StayGold variants enabled us to visualize subcellular dynamics more comprehensively and quantitatively in space and time than before.

## Results

### Assessment of monomericity or dispersibility of FPs

**Extended Data Fig. 1:**
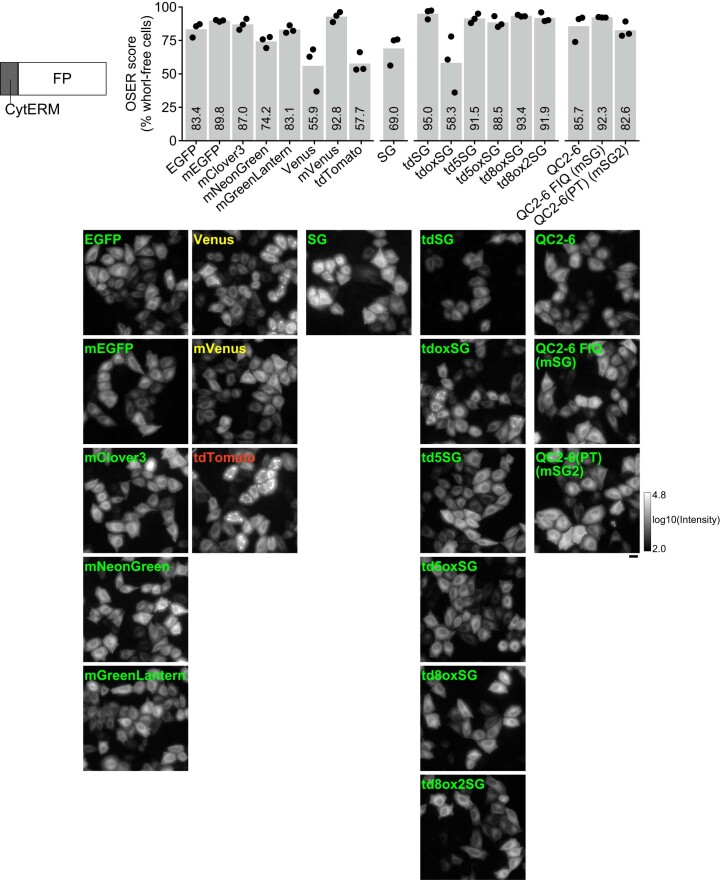
OSER assay for assessment of monomericity and dispersibility of StayGold variants and reference FPs. HeLa cells transfected with constructs encoding CytERM-FP were imaged using wide-field (WF) microscopy. StayGold is abbreviated as SG. The constructs are grouped into four categories: eight reference FPs, StayGold, tandem dimers, and monomers, from left to right. For each construct, the percentage of cells scored without visible whorl structures is plotted (black dots) (top) and one representative close-up image is shown (bottom). The logarithmic gray scale (2.0–4.8) indicates that the lowest and highest fluorescence intensities of all the images are 10^2.0^ and 10^4.8^, respectively. Transfection was repeated three times; the mean scores are shown by gray bars with numerical values (top). Scale bar, 20 µm. Source data

**Extended Data Fig. 2:**
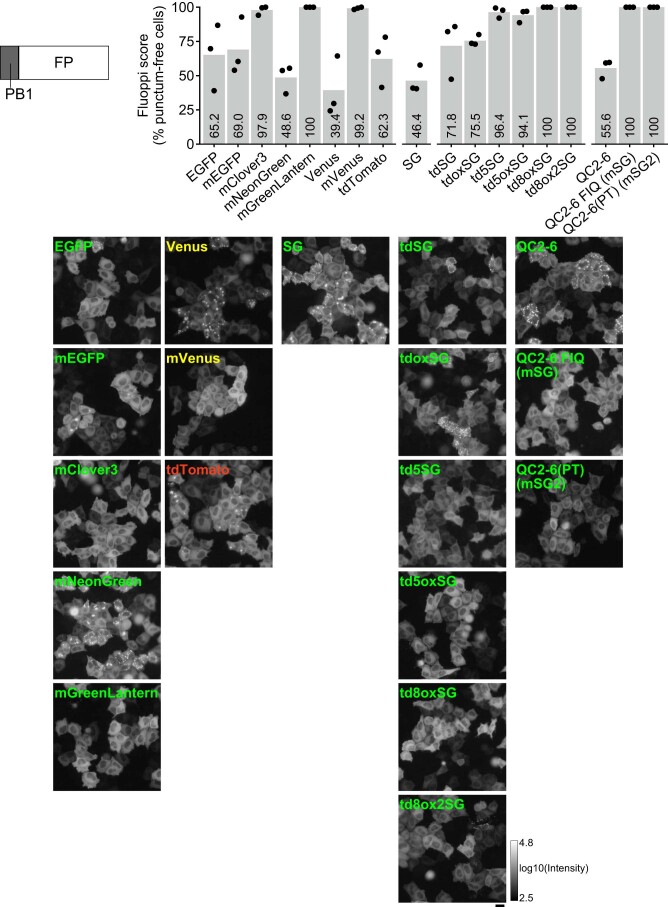
Fluoppi assay for assessment of monomericity and dispersibility of StayGold variants and reference FPs for C-terminal tagging. HeLa cells transfected with constructs encoding PB1-FP were imaged using WF microscopy. StayGold is abbreviated as SG. The constructs are grouped into four categories: eight reference FPs, SG, tandem dimers, and monomers, from left to right. For each construct, the percentage of cells scored without fluorescent puncta is plotted (black dots) (top) and one representative close-up image is shown (bottom). The logarithmic gray scale (2.5–4.8) indicates that the lowest and highest fluorescence intensities of all the images are 10^2.5^ and 10^4.8^, respectively. Transfection was repeated three times; the mean scores are shown by gray bars with numerical values (top). Scale bar, 20 µm. Source data

A previous OSER assay by Cranfill et al. investigated the monomeric quality of various FPs commonly used for molecular fusion applications^6^; monomeric FPs (mFPs) were basically characterized to show high OSER scores (percentages of whorl-free cells). We performed an OSER assay on eight common FPs^9–13^ (Extended Data Fig. 1). We confirmed the effect of the A206K mutation for monomerization of *Aequorea* sp. green fluorescent protein (GFP) variants^9^ (Supplementary Note 1). To evaluate FP monomericity or dispersibility on the basis of a different physicochemical principle, we took advantage of Fluoppi, the genetically encoded protein–protein interaction visualization system that harnesses the dynamics of condensed liquid-phase transitions (Supplementary Fig. 1)^3^. We developed a new method in which an FP of interest is simply fused to the Phox and Bem1p (PB1) domain of p62/SQSTM1. After transfection into cultured cells, oligomerization or assembly of the FP and homo-oligomerization of PB1 should result in crosslinking to form liquid-phase droplets that emit green fluorescence in the cytoplasmic compartment (Supplementary Fig. 2). We fused eight common FPs to the C terminus of PB1 for the assessment of their monomericity (Extended Data Fig. 2) and obtained results similar to those of the OSER assay except that PB1-mNeonGreen produced a substantial number of puncta.

### oxStayGold

We previously carried out combinatorial saturation mutagenesis on StayGold for cysteine residues. Simultaneous C174I and C208I mutations combined with an additional mutation H169Y led to the development of oxStayGold^1^. This variant of StayGold was engineered to efficiently label the ER from the inside^5^. Because oxStayGold was found to label all subcellular components, including the cytoplasm, very brightly, it has replaced StayGold in many experiments; however, oxStayGold remains a dimer.

### Tandem dimer constructs

**Extended Data Fig. 3:**
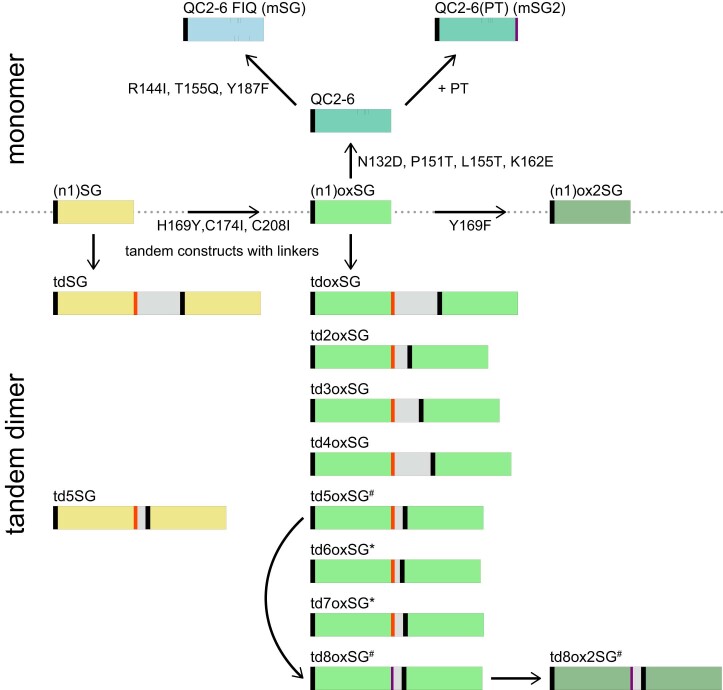
The evolution from (n1)StayGold to monomeric (top) and tandem dimer (bottom) variants. StayGold (SG) and its variants are distinctly colored as follows. SG: khaki, oxSG: light green, ox2SG: dark sea-green, QC2-6: seafoam blue, QC2-6 FIQ (mSG): light blue. Adaptors and linkers are represented with the following deep colors. n1: black, c4: orange red, PT: purple, linker (for tandem construction): gray. #The 21-residue linker (gray) was composed of a 12-residue linker (GHGTGSTGSGSS) and a possibly protease-resistant linker^2^. *Although we used two linkers of 13 and 22 residues reported in the tdTomato publication to generate td6oxSG and td7oxSG, respectively, neither of them matched td5oxSG. See Supplementary Fig. 3. Except QT2-6(PT) (mSG2), all the constructs shown here are used with no tagging or for tagging the C terminus of a host protein and thus have no c4 adaptor at their C-termini; please see Supplementary Fig. 14 concerning tagging the N terminus of a host protein.

The domain structures and amino acid sequences of td constructs are shown in Extended Data Fig. 3 and Supplementary Fig. 3, respectively. The original td construct (tdStayGold) was composed of n1, StayGold, c4, a 116-amino-acid linker (EV linker), n1 and StayGold, where n1 and c4 are adaptors that facilitate fusions to the N and C termini of StayGold, respectively^1^. In the OSER assay, the fusion of tdStayGold to CytERM labeled the ER network with moderate brightness and led to a very low propensity to form ER whorls, with an OSER score of 95.0% (Extended Data Fig. 1)^1^. Subsequently, oxStayGold was substituted in the td construct to create tdoxStayGold, which consistently achieved brighter molecular fusions than tdStayGold; however, we found that CytERM-tdoxStayGold showed an undesirable score (58.3%) (Extended Data Fig. 1). In Fluoppi assays, PB1-StayGold, PB1-tdStayGold and PB1-tdoxStayGold scored 46.4%, 71.8% and 75.5%, respectively (Extended Data Fig. 2), roughly corroborating the OSER assay results. To improve the dispersibility of tdoxStayGold, we re-examined the polypeptide linker between the two copies of (ox)StayGold. First, we trimmed the EV linker (116 residues) into three polypeptides that spanned 34, 66 and 97 amino acids; however, none of the td constructs with these shortened EV linkers (td2oxStayGold, td3oxStayGold and td4oxStayGold, respectively) showed improved OSER or Fluoppi scores. We subsequently harnessed the linker used for generating tdTomato^2,13^. The use of a 21-residue linker resulted in the generation of td5StayGold and td5oxStayGold, both of which gave high OSER scores (91.5% and 88.5%, respectively) (Extended Data Fig. 1) and high Fluoppi scores (96.4% and 94.1%, respectively) (Extended Data Fig. 2).

### Monomeric versions

**Fig. 1:**
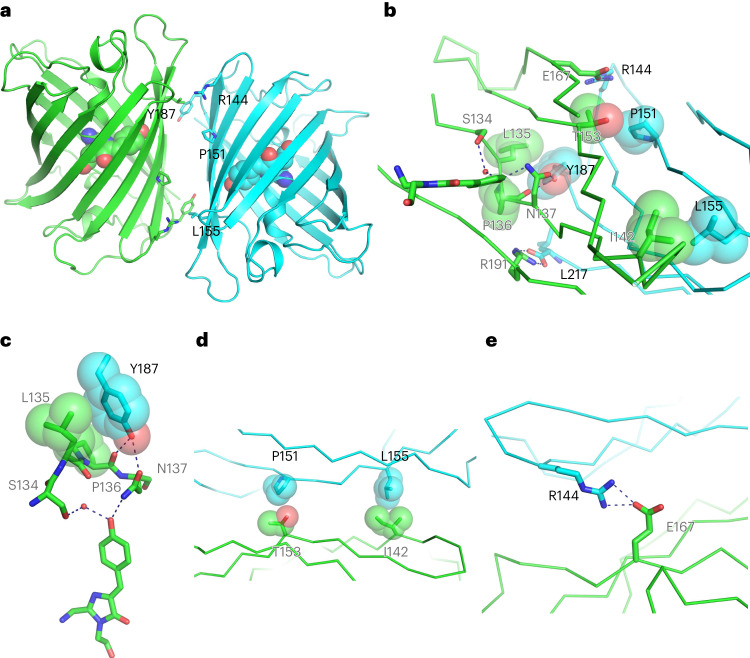
Dimeric structure of StayGold. **a**, Side view of the overall structure of the AC dimer with the chromophores (space-filling representation) and mutated residues (side chains) at the interface (stick representation). **b**–**e**, The dimer interface viewed from A protomer (**b**). Interacting side chains and the chromophore are represented in stick format. There is a salt bridge of Leu^217^ of C protomer and Arg^191^ of A protomer. Other interactions are resolved in three images (**c**–**e**) viewed from different angles. Detailed view of the interaction between Tyr^187^ (C protomer) and a four-amino-acid stretch (^134^SLPN^137^) that anchors the chromophore apex (A protomer) (**c**). Hydrophobic contact sites between Pro^151^ of C protomer and Thr^153^ of A protomer and between Leu^155^ of C protomer and Ile^142^ of A protomer (**d**). A salt bridge between Arg^144^ of C protomer and Glu^167^ of A protomer (**e**). One protomer (A) is shown in green and the other (C) in light blue (**a**–**e**). The atoms are color-coded as follows: carbon, green (A protomer) or light blue (C protomer); oxygen, red; nitrogen, blue. Mutated residues are displayed on the C protomer with black letters. The opposed residues are displayed on the A protomer with gray letters. The peptide backbones are shown in cartoon format (**a**) or as tubes (**b**, **d** and **e**). Hydrophobic interactions are shown with van der Waals surfaces of the side chains (**b**–**d**). Polar interactions are shown as dashed lines (**b**, **c** and **e**). See Extended Data Fig. 4.

**Extended Data Fig. 4:**
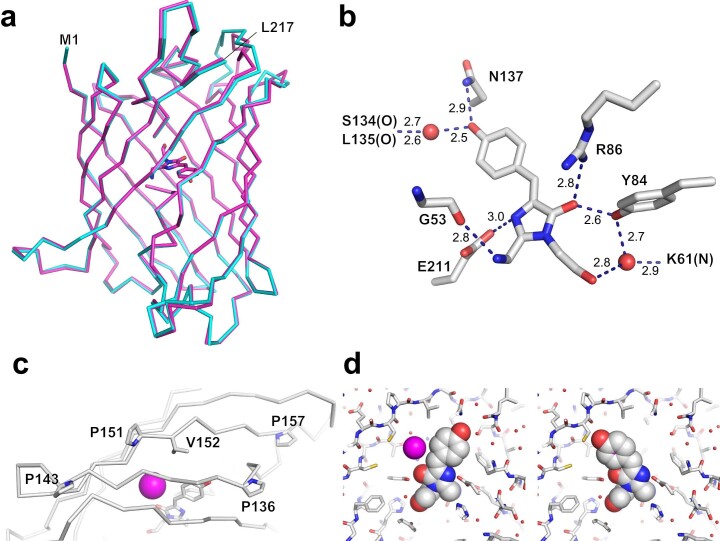
Crystal structure of StayGold. StayGold crystallized at pH 8.5 in space group *P*2_1_ and diffracted to 1.56 Å. The final model has an *R*_work_ of 18.63% and an *R*_free_ of 21.53%. StayGold also crystallized at pH 5.6 in space group *P*6_1_ and diffracted to 2.2 Å with the final model having an *R*_work_ of 15.47% and an *R*_free_ of 18.97%. See Supplementary Table 1 for a summary of the refinement statistics and model quality. The coordinates and structure factors have been deposited in the PDB data base (pH 8.5, entry ID: 8ILK; pH 5.6, entry ID: 8ILL). **a**, Superposition of the α-carbon backbone and chromophore (A protomer) of the two crystal structures (pH 8.5, cyan; pH 5.8, magenta). The root-mean-square deviation value was calculated to be 0.664 Å. M1 and L217 indicate the N- and C-terminal ends, respectively. Because of its higher resolution, the pH 8.5 crystal structure is used hereafter for characterizing the atomic structure of StayGold. **b**, The chromophore and surrounding side chains represented in *stick* format. Hydrogen bonds are represented as broken lines with indicated distances in angstroms. Water molecules are indicated as red spheres. **c**, Four proline residues (Pro^136^, Pro^143^, Pro^151^, and Pro^157^) located on the seventh and eighth β-strands, which are close to the hydroxy group of the *p*-hydroxyphenyl ring of the chromophore in the *cis* conformation (*stick* representation). These prolines may rigidify this region of the β-barrel, thus stabilizing the chromophore in the *cis* state. It is noted that most fluorescent proteins have only one proline in the corresponding region. The side chain of Val^152^ may also contribute to the stabilization of the chromophore. During the crystal structure refinement, placement of a chloride ion was found to account for the peak observed in the *Fo–Fc* map near the chromophore. The chloride ion (indicated as a magenta sphere) is expected to sterically prevent the chromophore from adopting a *trans* conformation. **d**, The chromophore (represented in the *space-filling* format) is positioned in its *cis* (left) or *trans* (right) conformation. In the latter case, there is an apparent collision against the chloride ion. **b–d**, The atoms are color-coded as follows: carbon, white; oxygen, red; nitrogen, blue; sulfur, yellow.

We determined the crystal structure of StayGold to 1.56 Å resolution (Fig. 1, Extended Data Fig. 4 and Supplementary Table 1). The solved structure also shows the dimer formation of this FP as well as the amino acids around the chromophore. In the present study, we focused on the dimeric interface to adopt the monomerization approach^2^ using (n1)oxStayGold as a starting material (Extended Data Fig. 3 and Supplementary Fig. 3). We gradually introduced mutations into the interface to generate dozens of monomeric versions (Supplementary Note 2). We comprehensively prepared several libraries of (n1)oxStayGold variants that carried different partial mutations at the interface. In one attempt, we focused on threonine substitution at Pro^151^ and Leu^155^ and screened candidates in a library of (n1)oxStayGold P151T/L155T iteratively with multiple cycles of random mutagenesis to produce QC2-6. This variant contained four mutations (P151T, L155T, N132D and K162E) relative to (n1)oxStayGold. When observing the fluorescence in the cytosolic and nuclear compartments of transfected cells under intense illumination, we confirmed that QC2-6 was highly photostable and bright. When QC2-6 was expressed in bacteria and purified, the protein product showed monomer-like behavior in pseudonative SDS–PAGE analysis (Supplementary Fig. 4). Transfection of CytERM-QC2-6 into HeLa cells generated only a small number of ER whorls in an OSER assay (score, 85.7%) (Extended Data Fig. 1). We noticed, however, that PB1-QC2-6 yielded a substantial number of puncta in a Fluoppi assay (score, 55.6%) (Extended Data Fig. 2). With this unacceptable result, we decided to drive the monomerization further on the basis of QC2-6. Performing combinatorial saturation mutagenesis at Tyr^187^, Arg^144^ and Thr^155^ and screening for brightness, photostability and monomericity yielded QC2-6 FIQ, which contained Y187F, R144I and T155Q mutations relative to QC2-6. QC2-6 FIQ gave high scores for both OSER (92.3%) (Extended Data Fig. 1) and Fluoppi (100%) (Extended Data Fig. 2) assays.

### A new appendage at the StayGold C terminus

In one experiment to amplify the full length of QC2-6, we accidentally used a defective reverse primer to introduce a mutation at the termination codon. The translation readthrough led to the addition of a short C-terminal tail (PT) composed of YSRTKLE. Notably, this protein product QC2-6(PT) (Extended Data Fig. 3 and Supplementary Fig. 3) showed high scores in OSER (82.6%) (Extended Data Fig. 1) and Fluoppi (100%) (Extended Data Fig. 2) assays. Because the StayGold dimeric interface seems to involve the carboxyl group of the end residue (Leu^217^), such a simple extension might improve the monomericity of QC2-6; however, the reality is complicated. For example, c4 did not replace PT in this situation; c4 works well as an adaptor but not as a tail at the C terminus. Assuming that QC2-6(PT) could be a potentially useful byproduct, we considered its further characterization worthwhile (see below).

We found that PT can be used as another adaptor for fusion at the StayGold C terminus. By replacing c4 with PT in td5oxStayGold, we generated td8oxStayGold. In addition, we performed saturation mutagenesis on td8oxStayGold for Tyr^169^ and generated td8ox2StayGold with a Y169F mutation. Both td8oxStayGold and td8ox2StayGold exhibited excellent dispersibility (Extended Data Figs. 1 and 2).

### Photostability

**Fig. 2:**
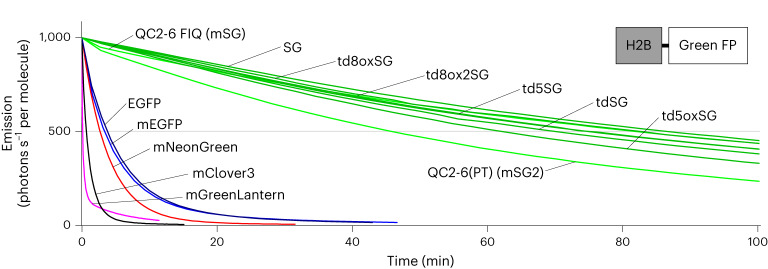
Photostability of StayGold variants and reference green-emitting FPs in live cells. Plot of intensity versus normalized total exposure time, with an initial emission rate of 1,000 photons s^−1^ per molecule. FPs were expressed as fusions to H2B in HeLa cells. Illumination intensity, 8.66 W cm^−2^. The curves shown are representative of three repetitions (*n* = 3 independent experiments). The statistical values of *t*_1/2_ (time for photobleaching from an initial emission rate of 1,000 photons s^−1^ per molecule down to 500) are shown in Table 1. SG, StayGold. Curves are colored as follows. QC2-6 FIQ (mSG) and QC2-6(PT) (mSG2), green; SG and its tandem dimers, dark green; EGFP, dark blue; mEGFP, blue; mNeonGreen, red; mClover3, black; mGreenLantern, magenta. Intensity-normalized curves are shown in Supplementary Fig. 6. Source data

To examine the performance of monovalent tagging with bright and photostable fluorescence, we characterized four tandem dimers (td5StayGold, td5oxStayGold, td8oxStayGold and td8ox2StayGold) and two monomers (QC2-6 FIQ and QC2-6(PT)) of StayGold. After preparing purified protein products, we determined the extinction coefficients (εs) (Supplementary Fig. 5) and the fluorescence quantum yields (QY_f_s) of these six variants (Table 1). Their molecular brightness (the product of ε and QY_f_) was nearly the same as that of StayGold. To assess their photostability, we expressed them in comparison with five reference green-emitting FPs (EGFP, mEGFP, mClover3, mNeonGreen and mGreenLantern) in cultured HeLa cells as fusions to histone 2B (H2B) to be immobilized on the chromatin structures inside the nucleus^14^. We exposed live-cell samples to continuous illumination under an unattenuated light-emitting diode (LED) lamp (Supplementary Fig. 6) and normalized the photobleaching curves using the standard method that considers the molecular brightness of each FP^13–15^(Fig. 2). The time required for photobleaching from an initial emission rate of 1,000 to 500 photons s^−1^ molecule^−1^ (*t*_1/2_) of each FP is presented in Table 1. All of the new variants are clearly similar to the original StayGold in terms of photostability and molecular brightness and are thus more than one order of magnitude more photostable than any currently available FPs.

**Table 1:** Characteristics of common green-emitting FPs, StayGold and its variants

	λab^a^/λem^b^ (nm)	ε^c^ (10^3^ M^−1^cm^−1^)		QY_f_^d^	Brightness		Photostability *t*_1/2_ (s)^g^	Monomericity/dispersibility (% normal-looking cells)		
		λab	483		Mol^e^	Cell^f^		OSER	Fluoppi	
								CytERM-FP	PB1-FP	FP-PB1
EGFP	488/509	51	51	0.71	36	0.22	227 ± 9	83.4	65.2	NT
mEGFP	488/509	56.5	56.5	0.70	40	NT	203 ± 7	89.8	69.0	NT
mClover3	505/518	99	52	0.84	83	NT	40 ± 3	87.0	97.9	NT
mNeonGreen	505/518	112	64	0.87	97	0.32	162 ± 6	74.2	48.6	NT
mGreenLantern	501/515	117	76	0.74	87	0.51	6.5 ± 0.2	83.1	100	NT
StayGold	496/505	159	105	0.93	148	1.00	5,190 ± 138	69.0	46.4	20.7
tdStayGold	496/504	162	108	0.90	146	NT	4,198 ± 106	95.0	71.8	64.6
tdoxStayGold	496/504	NT	NT	NT	NT	NT	NT	58.3	75.5	64.5
td5StayGold	496/504	155	103.4	0.91	141	2.09	4,491 ± 201	91.5	96.4	90.1
td5oxStayGold	496/506	156	102.6	0.93	145	2.71	3,753 ± 340	88.5	94.1	60.1
td8oxStayGold	496/506	157	103	0.93	146	2.73	4,958 ± 137	93.4	100	97.5
td8ox2StayGold	496/506	159	105	0.93	148	2.77	4,495 ± 177	91.9	100	93.9
QC2-6	496/504	NT	NT	NT	NT	NT	NT	85.7	55.6	NT
QC2-6 FIQ (mStayGold)	499/510	164	100	0.83	136	0.95	4,898 ± 199	92.3	100	100
QC2-6(PT) (mStayGold2)	499/509	175	110	0.79	138	0.71	2,753 ± 172	82.6	100	98.2

^a^Absorbance maximum.

^b^Emission maximum.

^c^Absolute extinction coefficient at λab (left) and 483 nm (right). The measurement was based on the fact that after alkali denaturation of these FPs, the chromophore, containing a dehydrotyrosine residue conjugated to the imidazolone group, absorbs light maximally at 447 nm with a molar extinction coefficient of 44,000 M^−1^ cm^−1^ (ref. ^45^). See Supplementary Fig. 5.

^d^Fluorescence quantum yield measured using an absolute photoluminescence quantum yield spectrometer.

^e^Product of ε (λab) and QY_f_. This value reflects the molecular brightness of an FP^46^.

^f^Cellular brightness calculated from data shown in Fig. 3b. The fluorescence from each green-emitting FP (with excitation at 483 nm) was corrected for mCherry fluorescence and spectral throughput (Supplementary Fig. 7) and then normalized to that of StayGold. Equimolar coexpression of a green-emitting FP and mCherry using the bicistronic expression system^12^.

^g^Time in seconds to reduce the emission rate from 1,000 to 500 photons s^−1^ per molecule under WF illumination. Live HeLa cells expressing H2B-FP fusions were used (Fig. 2). Values are mean ± s.d. (*n* = 3 independent experiments). All values were measured by us (underlined, in a previous study^1^; otherwise, in this study). NT, not tested. QC2-6 FIQ and QC2-6(PT) were designated as mStayGold (mSG) and mStayGold2 (mSG2), respectively.

### Practical brightness

**Fig. 3:**
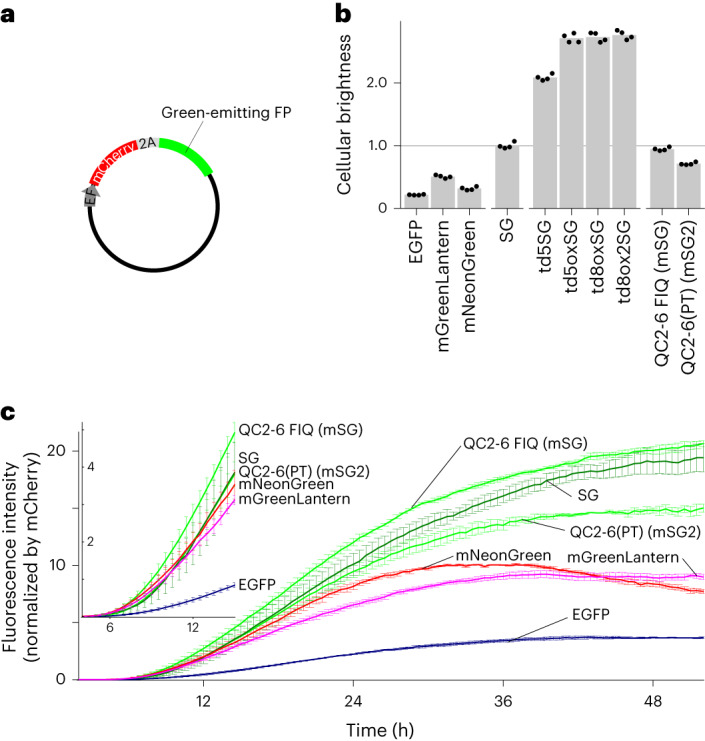
Brightness of StayGold variants and reference green-emitting FPs in live cells. **a**, Cotranslation of green-emitting FP with mCherry using the bicistronic coexpression system. Transfection was performed with pCSII-EF/mCherry-T2A-green-emitting FP. **b**, Cellular brightness 48 h after transfection. The green fluorescence was corrected for the mCherry fluorescence and spectral throughput (Supplementary Fig. 7) and normalized to that of StayGold (Supplementary Table 2). Transfection was repeated four times; the mean values are shown by gray bars and are reported in Table 1 (cellular brightness). **c**, Fluorescence development after transfection. Side-by-side comparison of six green-emitting FPs for their chromophore maturation using an automated time-lapse imaging system that accommodates a six-well plate. The green fluorescence was corrected for the mCherry fluorescence (*t* = 48 h) (Supplementary Note 8) and spectral throughput (Supplementary Fig. 8). Data points are shown as mean ± s.e.m. (*n* = 3 independent experiments). Curves are colored as follows. QC2-6 FIQ (mSG) and QC2-6(PT) (mSG2), green; SG, dark green; EGFP, dark blue; mNeonGreen, red; mGreenLantern, magenta. Inset shows curves during the early stage. Source data

We next examined the actual brightness of the six aforementioned StayGold variants in living cells in comparison with StayGold and three reference green-emitting FPs (Fig. 3 and Table 1). We used an expression system (cotranslation via the T2A peptide) (Fig. 3a) to correct the brightness of each green-emitting FP by that of mCherry. We also considered the spectral throughput of each green-emitting FP in the imaging system to ensure an unbiased comparison (Supplementary Fig. 7and Supplementary Table 2). We determined the cellular brightness that reflected the FP maturation yield at a specific time point (48 h after transfection) (Fig. 3b). EGFP, mGreenLantern and mNeonGreen showed 22%, 51% and 32% brightness, respectively, compared to StayGold. Also, all the td constructs were two to three times brighter than StayGold, indicating the general merit of such td constructs for doubling the fluorescence brightness per unit of host protein compared to the brightness achieved with the conventional monomer. We noted that QC2-6 FIQ and QC2-6(PT) showed similar cellular brightness to StayGold, demonstrating the practical usefulness of these two StayGold monomers. Then, we used an automated live-cell time-lapse imaging system to evaluate the maturation speed of these two monomers in comparison with those of StayGold and the above three reference green-emitting FPs. After correction for spectral throughput and mCherry fluorescence (Supplementary Fig. 8), we found that QC2-6(PT) matured as fast as StayGold, mNeonGreen and mGreenLantern and that QC2-6 FIQ matured faster than any of the other FPs examined here (Fig. 3c). The excellent maturation of QC2-6 FIQ compared to StayGold and QC2-6(PT) was confirmed by classic experiments that used bacteria (Supplementary Note 3, Supplementary Fig. 9 and Supplementary Video 1).

All things considered, we designated QC2-6 FIQ as the most useful monomeric version and named it mStayGold. We also kept QC2-6(PT) as a reserve monomer and named it mStayGold2. Their fluorescence was slightly red shifted compared to that of StayGold (Supplementary Fig. 10). mStayGold and mStayGold2 showed fluorescence lifetimes of 2.79 ± 0.01 ns and 2.83 ± 0.02 ns, respectively (mean ± s.d., *n* = 6) and both exhibited a fluorescence pKa value of 4.8 (Supplementary Fig. 11). Despite the possibility that the chromophore may be affected by an external chloride ion (Extended Data Fig. 4), their absorption and fluorescence were resistant to high concentrations of KCl (Supplementary Fig. 12). Last, neither mStayGold nor mStayGold2 exhibited reversible photo-switching behavior (Supplementary Fig. 13). These results suggest that mStayGold and mStayGold2 produce stable fluorescence under normal physiological conditions.

### N- or C-terminal tagging with StayGold variants

**Extended Data Fig. 5:**
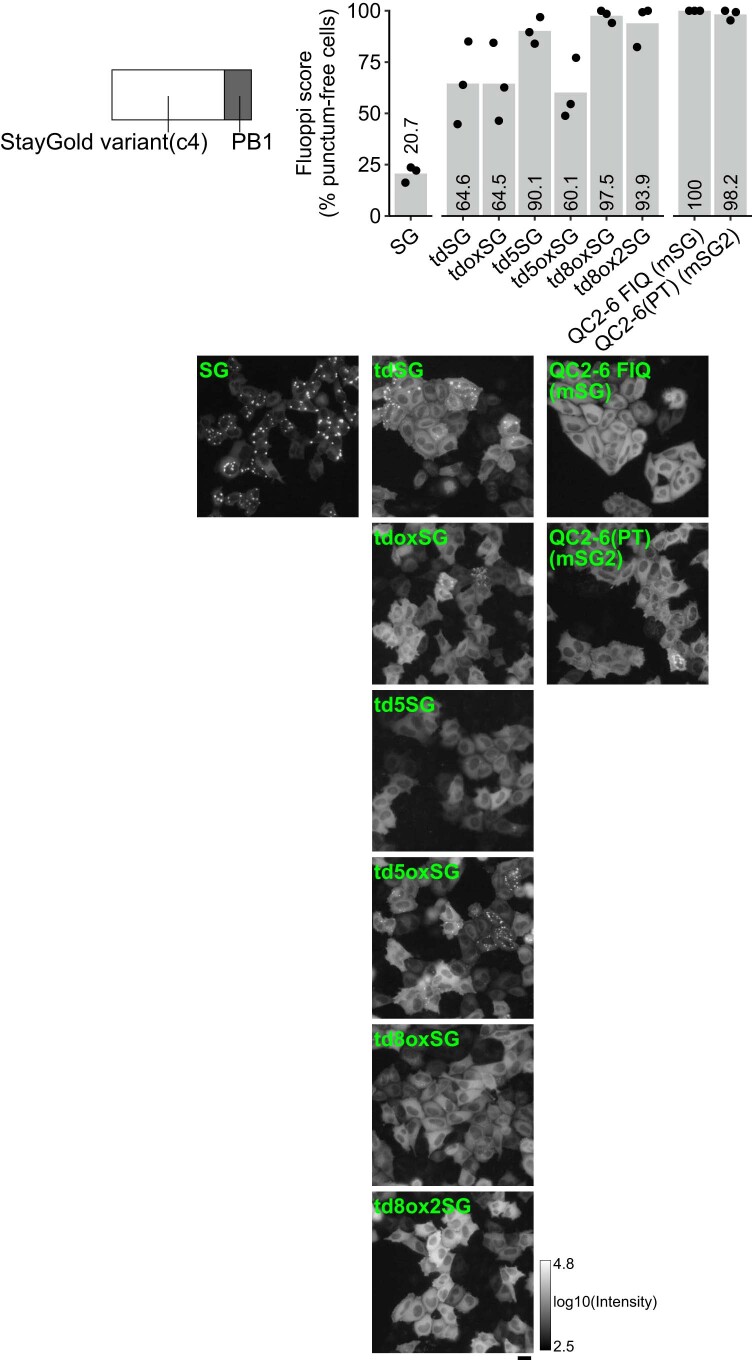
Fluoppi assay for assessment of monomericity and dispersibility of StayGold variants for N-terminal tagging. HeLa cells transfected with constructs encoding FP-PB1 were imaged using WF microscopy. StayGold is abbreviated as SG. The constructs are grouped into three categories: SG, tandem dimers, and monomers, from left to right. For each construct, the percentage of cells scored without fluorescent puncta is plotted (black dots) (top) and one representative close-up image is shown (bottom). The logarithmic gray scale (2.5–4.8) indicates that the lowest and highest fluorescence intensities of all the images are 10^2.5^ and 10^4.8^, respectively. Transfection was repeated three times; the mean scores are shown by gray bars with numerical values (top). Scale bar, 20 µm. Source data

A guide to the design of C-terminal or N-terminal tagging of a protein of interest with StayGold variants is provided in Supplementary Fig. 14. All the StayGold variants have an n1 adaptor at their N terminus and are useful for C-terminal tagging, as observed for CytERM-FP (Extended Data Fig. 1) and PB1-FP (Extended Data Fig. 2). In this case, the original C terminus of StayGold can be safely kept; its extension with polypeptides seems to affect expression of the fusions. An exception is the PT adaptor, which can function also as a C-terminal tail. By contrast, tagging a protein at the N terminus with a StayGold variant requires an adaptor (c4 or PT) at the FP C terminus (Supplementary Note 4). Whereas CytERM permits only C-terminal tagging, PB1 can be tagged at both termini. Thus, we fused StayGold and its variants to the N terminus of PB1 via the c4 adaptor for a Fluoppi assay (Extended Data Fig. 5). Whereas StayGold(c4)-PB1 exhibited a low score (20.7%), high Fluoppi scores were obtained with td5StayGold (90.1%), td8ox2StayGold (93.9%), mStayGold (100%) and mStayGold2 (98.2%).

### Condensin I as a migrant chromosome stabilizer

**Fig. 4:**
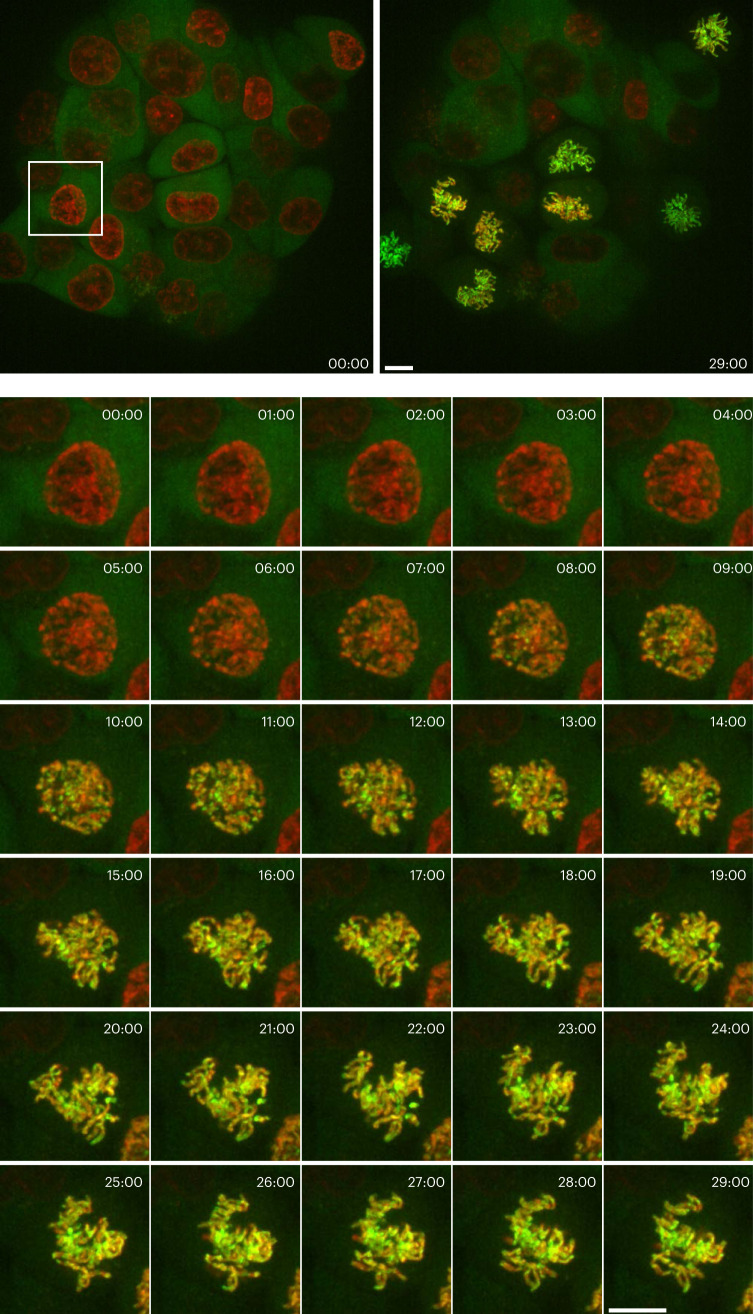
Visualization of chromosome targeting of td5oxStayGold-tagged condensin I at low copy number expressed via a genome-editing technique. After release from cell cycle arrest, genome-edited HCT116 cells (#897) were imaged for CAP-H-td5oxStayGold (at 488 nm excitation) and SiR-DNA-labeled chromosomes (at 637 nm excitation) using spinning-disk LSCM (SpinSR10) at the indicated times (min:s). Every 1 min, three-dimensional (3D) scanning was executed with a *z* step size of 1 μm over an axial range of 13 μm and the green and far-red fluorescence images were merged. Maximum intensity projection (MIP) images are shown. Time-series image data of the cell (bottom) boxed in the entire field of view (top). Representative of *n* = 3 independent experiments. Scale bars, 10 μm. See Supplementary Video 2.

**Extended Data Fig. 6:**
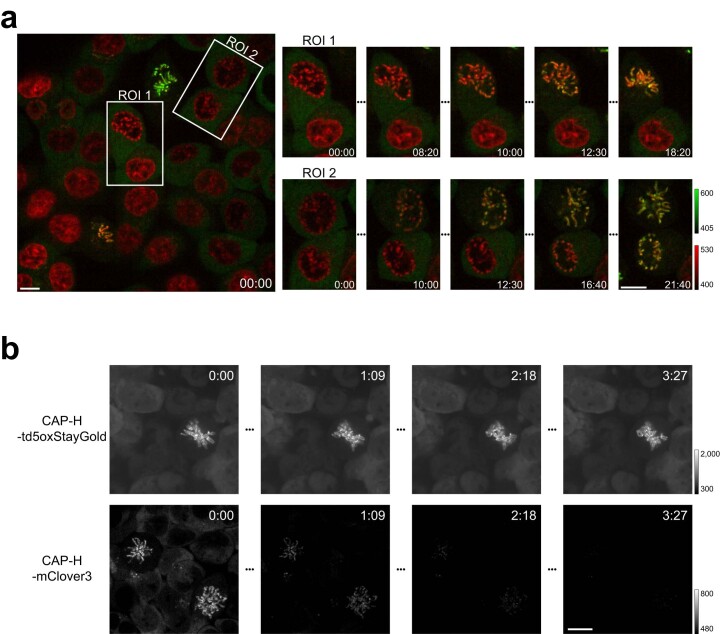
High-speed visualization of chromosome targeting of FP-tagged condensin I in genome-edited HCT116 cells. **a**, After release from cell cycle arrest, genome-edited HCT116 cells (#897) were imaged under 488 nm excitation for observation of CAP-H-td5oxStayGold and under 637 nm excitation for observation of SiR-DNA–labeled chromosomes by spinning-disk LSCM (SpinSR10) at 1 frame per second. Merged images at the indicated times (min:s). Scale bars, 10 µm. See Supplementary Video 3. **b**, Photostability comparison between CAP-H-td5oxStayGold and CAP-H-mClover3 under the same optical conditions. Illumination intensity, 2.24 W cm^−2^. Genome-edited HCT116 cells (#897, top vs. #899, bottom) during prometaphase were volume imaged (*z*-step, 0.25 µm; *z*-range, 2.5 µm) every 6.9 s over a total period of 278 s, with continuous excitation at 488 nm. Each gray scale indicates that the lowest and highest fluorescence intensities. Scale bar, 10 µm. See Supplementary Video 4.

During mitosis, condensins play a central role in chromosome assembly and segregation^16^. Two types of condensin complexes are present in most eukaryotic cells: condensin I and condensin II. Whereas condensin II binds to chromosomes throughout the cell cycle, condensin I does so in a specific time window; it is excluded from the nucleus in interphase and prophase but associates with mitotic chromosomes after nuclear envelope breakdown (NEBD), which causes a mixing of nuclear and cytoplasmic macromolecules. It is thus interesting to visualize at high-spatiotemporal resolution how this cytoplasmic chromosome-stabilizing factor gets to interact with mitotic chromosomes. In a previous seminal study, HeLa cell clones stably expressing functional EGFP-tagged subunits of condensin I were generated to time-lapse image volumes every 1 min using single-beam laser-scanning confocal microscopy (LSCM)^17^. After a lapse of many years, we took a more advanced approach in the present study. Specifically, we used the CRISPR-Cas9 genome-editing technique^18,19^ to produce HCT116 cells in which a subunit of condensin I (CAP-H) was endogenously tagged at the C terminus with td5oxStayGold (CAP-H-td5oxStayGold) (Supplementary Fig. 15). Before observation, nuclei were stained with SiR-DNA, which is a far-red, fluorogenic, cell permeable and highly specific live-cell DNA probe. We used SpinSR10, a type of spinning-disk LSCM, to image a physiological concentration of CAP-H-td5oxStayGold in parallel with chromosomal DNA in multiple mitosing cells. First, we carried out a volumetric imaging experiment in which *z*-stack images were acquired every 1 min for 30 min (Fig. 4 and Supplementary Video 2). In a different experiment, we carried out a high-speed continuous imaging at one *z* position at a temporal resolution of one frame s^−1^ for 30 min (Extended Data Fig. 6a and Supplementary Video 3). These two imaging modes complementarily followed the targeting behavior of condensin I to chromosomes. During prophase, the far-red fluorescence of SiR-DNA was partially condensed inside the nucleus and the green fluorescence of CAP-H-td5oxStayGold was homogeneously distributed in the cytoplasmic compartment. After NEBD began in late prophase, all the condensing chromosomes became green fluorescent in a concerted manner within the nucleus. The green fluorescence was highly concentrated in the centromeric region; such uneven distribution of the green/far-red ratio in each chromatid was invariable subsequently. Thus, our fast continuous sustainable imaging revealed that CAP-H molecules associate in unison with mitotic chromosomes. This process being limited by reaction rather than diffusion may corroborate the previous finding that the association occurs in a single kinetic binding step^17^.

In prometaphase, the CAP-H-td5oxStayGold signals clearly delineated two closely associated sister chromatids. The bright and photostable labeling enabled continuous volumetric imaging (Extended Data Fig. 6b and Supplementary Video 4), which revealed that fully condensed chromosomes swayed constantly in a unified body, likely because of their attachment to spindle microtubules at this stage. We used the CRISPR-Cas9 technique to generate HCT116 cells expressing CAP-H-mClover3 (Supplementary Fig. 15) and found the labeling vulnerable to the same illumination (Extended Data Fig. 6b and Supplementary Video 4).

### Golgi membrane dynamics

As discussed earlier regarding the OSER assay mechanism, fluorescent membrane labeling, in principle, requires monovalent fusion of an FP to a membrane-resident protein. To visualize the Golgi apparatus, we previously fused StayGold through c4 to the N terminus of a region (amino acids 3,131–3,259) of human giantin^1,20^, the gigantic Golgi matrix protein. This region, which we refer to as GianCreg (giantin C-terminal region), consists of a short cytoplasmic domain, a membrane-spanning domain and a short luminal C-terminal domain and is suggested to form a dimer via a disulfide bond in the lumen^21^. Accordingly, we do not consider obligate dimer formation of StayGold to be a serious problem in the labeling approach; however, as it is not clear how the monomer/dimer equilibrium of giantin is regulated, we substituted td5StayGold to create td5StayGold(c4)=GianCreg in this study.

**Fig. 5:**
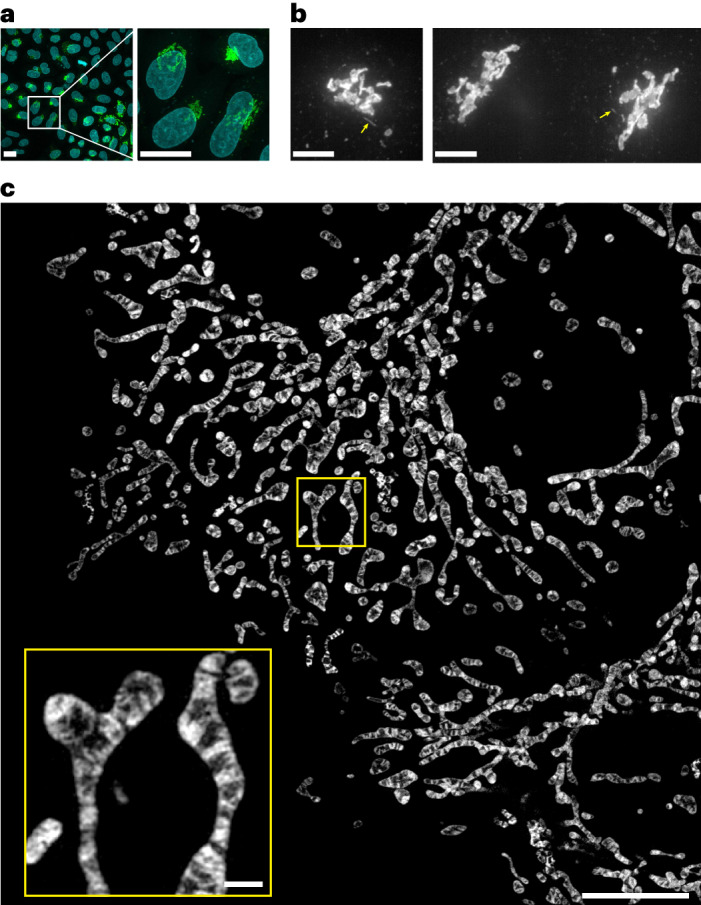
Visualization of td5StayGold-harboring Golgi membranes and mStayGold-harboring inner mitochondrial membranes. **a**, Confocal images of td5StayGold(c4)=GianCreg (green) and DAPI (cyan) in fixed HeLa cells. A MIP image (20 slices, 1.0-μm *z* step). Low (left) and high (right) magnifications. Scale bars, 20 μm. **b**, Volumetric and continuous imaging of HeLa cells expressing td5StayGold(c4)=GianCreg by SDSRM (SpinSR10) revealed the occurrence of fast-moving tubular structures that emerged from the Golgi apparatus (yellow arrows). Two independent experiments (left and right). Scale bars, 5 μm. See Supplementary Video 5. Similar results were obtained from 17 other independent cultured cell samples. **c**, A MIP of HeLa cells expressing COX8a=mStayGold. Cells were 3D scanned with a *z* step size of 0.11 μm over an axial range of 2.08 μm by lattice SIM (Elyra 7). SIM^2^ was used for image reconstruction. This MIP corresponds to the image at *t* = 126 s in Supplementary Video 9. Scale bars, 10 μm, 1 μm (inset). Representative of *n* = 3 independent samples.

We transfected cultured HeLa cells transiently with the constructed complementary DNA. The Golgi stack comprising several flattened cisternae and many vesicular structures were highlighted against the 4′,6-diamidino-2-phenylindole (DAPI)-stained nucleus in each transfected cell (Fig. 5a). Continuous, cell-wide observation using SDSRM (SpinSR10) enabled us to track movements of the Golgi apparatus and its membranous derivatives, including long tubular structures emerging from the cisternae (Fig. 5b and Supplementary Video 5). The Golgi-derived tubules exhibited irregular dynamics^22,23^ but mostly detached and moved away from the Golgi stack, similar to the dynamics of tubular post-Golgi carriers^24,25^. We subsequently attempted to image the microtubule network that should guide Golgi-derived vesicles throughout the cell. We noticed substantial photobleaching of far-red chemical dyes for microtubules, such as SiR-Tubulin, under our imaging conditions and therefore transfected td8ox2StayGold(c4)=β-tubulin into cells in addition to td5StayGold(c4)=GianCreg to achieve eccentric dual-target imaging in a single channel. We tracked all the labeled vesicles, including long tubular ones, moving along microtubules over extended periods (Supplementary Video 6 and Supplementary Fig. 16).

**Extended Data Fig. 7:**
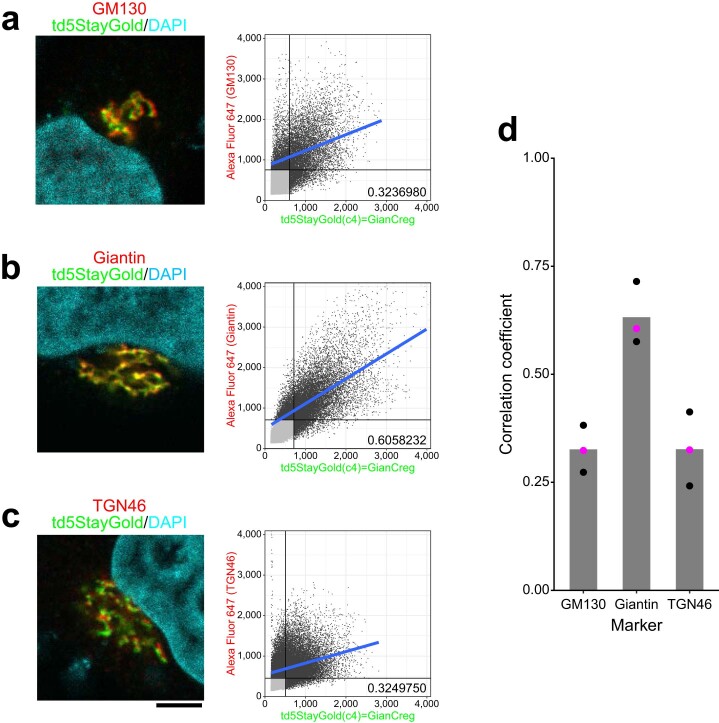
Immunocytochemistry for localizing td5StayGold(c4)=GianCreg within the Golgi complex. HeLa cells stably expressing td5StayGold(c4)=GianCreg were chemically fixed and immunostained for three Golgi markers: GM130 (*cis*), Giantin (medial), and TGN46 (*trans*-Golgi network). Their immunosignals (Alexa Fluor 647) were imaged with td5StayGold fluorescence three-dimensionally with a *z*-step of 0.52 µm using a confocal microscope. For a scan format of 512 × 512 and a zoom factor of 4×, the pixel size was 0.104 µm, which was sufficiently small to make good use of the resolving power of the high–numerical-aperture (NA 1.2) objective lens. **a–c**, Analysis of colocalization between td5StayGold(c4)=GianCreg and GM130 (**a**), Giantin (**b**), or TGN46 (**c**) in a single experiment. *left*, Representative single-slice images. Nuclei were stained with DAPI. Scale bar, 5 µm. *right*, Pearson correlation plots based on 26 (**a** and **b**) and 27 (**c**) *z*-slices, showing the correlation coefficients: 0.324 (**a**), 0.606 (**b**), and 0.325 (**c**). **d**, The experiment was repeated three times. Individual correlation coefficients are plotted; the data plots derived from **a–c** are highlighted in magenta). The mean values are shown by gray bars. Source data

We observed similar fluorescence patterns of the Golgi apparatus in HeLa cells that stably expressed td5StayGold(c4)=GianCreg. These cell samples were fixed and subjected to immunocytochemical studies using various Golgi markers for identification of the fluorescently labeled components. We observed the most significant overlap with giantin (medial)^21^ and partial overlap with GM130 (*cis*)^26^ and TGN46 (*trans*-Golgi network)^27^ signals (Extended Data Fig. 7). These results suggested that td5StayGold was most concentrated in the medial Golgi but distributed throughout the Golgi membrane, possibly due to the N-terminal truncation and/or over expression.

### mStayGold targeting filamentous actin

**Extended Data Fig. 8:**
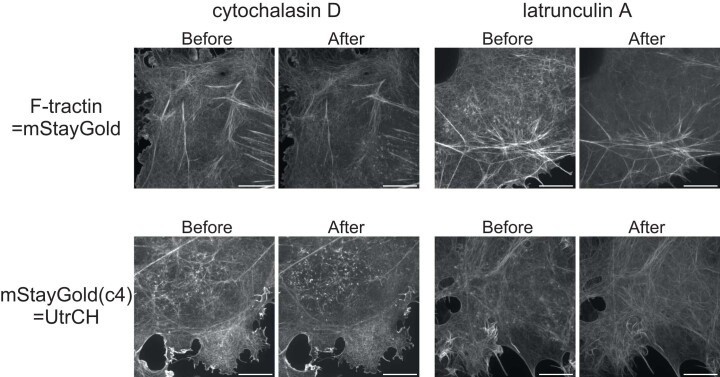
Effects of drugs on F-actin organization. COS-7 cells expressing F-tractin=mStayGold (top) or mStayGld(c4)=UtrCH (bottom) were treated with cytochalasin D (left) or latrunculin A (right) during continuous imaging by SDSRM (SpinSR10) (Supplementary Video 8). UtrCH: utrophin calponin homology domain. Single confocal images sectioned nearest the cell bottom were acquired. A pair of representative images (Before and After) are shown for each experiment. Scale bars, 10 µm. Punctate structures accumulated on the plasma membrane mostly below the nucleus after the application of cytochalasin D was applied. By contrast, they disappeared after the application of latrunculin A was applied. The images shown are representative of *n* = 12 independent experiments for cytochalasin D/F-tractin=mStayGold, *n* = 7 independent experiments for latrunculin A/F-tractin=mStayGold, *n* = 7 independent experiments for cytochalasin D/mStayGold(c4)=UtrCH, *n* = 4 independent experiments for latrunculin A/mStayGold(c4)=UtrCH.

Size-wise, FP monomers should be more suitable than FP td constructs for labeling protein networks and complexes that are susceptible to steric hindrance. To study filamentous actin (F-actin) dynamics in live cells, we tagged actin-binding domains^28^ with mStayGold. First, we simply co-transfected Lifeact-mStayGold into Vero cells with F-tractin-mScarlet-I and confirmed their colocalization on stress fibers, actin bundles in filopodia, actin networks in lamellipodia and cortical actin networks underlining the plasma membrane (Supplementary Fig. 17). We then used SDSRM (SpinSR10) to continuously image COS-7 cells expressing F-tractin=mStayGold or mStayGold(c4)=UtrCH in single confocal slices sectioned nearest the cell bottom (Supplementary Fig. 18). Whereas stress fibers tended to be static, filopodia and lamellipodia were constantly moving. Cortical networks spread like cobwebs and some loose wires showed active fluctuations (Supplementary Video 7). Small ordered architectures came out to ‘surf’ on the cortical networks actively. They were mostly asterisk-like structures that could be categorized as actin stars and asters^29,30^. Occasionally they swirled about to form rings that have been speculated to reflect endocytic machineries^25^ or actin vortices^29^. These punctate structures disappeared quickly after the application of latrunculin A, a drug that sequesters monomeric actin; they were stabilized on the plasma membrane by the application of cytochalasin D, a drug that caps filament plus ends (Extended Data Fig. 8 and Supplementary Video 8).

### mStayGold targeting inner mitochondrial membranes

Imaging the inner mitochondrial membrane (IMM) live is important for understanding the development of many diseases but remains challenging^31,32^. To date, IMM-selective organic dyes or SNAP-tagged IMM-resident proteins have been used mainly in combination with stimulated emission depletion (STED) microscopy^33–35^; however, the number of images acquired has been limited, likely because of substantial photobleaching of the dyes. The development of photostable chemical fluorophores that stain IMMs, such as MitoPB Yellow^36^ and MitoESq-635 (ref. ^37^), greatly increased the spatial resolution of cristae imaging by STED microscopy; however, the extremely strong laser light brought a situation where stained mitochondria were time-lapse imaged with long time intervals (>50 s)^37^ or were otherwise photodegraded rapidly (<1 min)^36^. On the other hand, state-of-the-art deconvolution^38,39^ or generative artificial intelligence methods^40^ greatly reduced the excitation light of SIM, enabling long-duration high-spatiotemporal resolution imaging of mitochondrial cristae; however, the computational approaches assume a priori knowledge and may miss rare but important changes in IMM structures.

**Extended Data Fig. 9:**
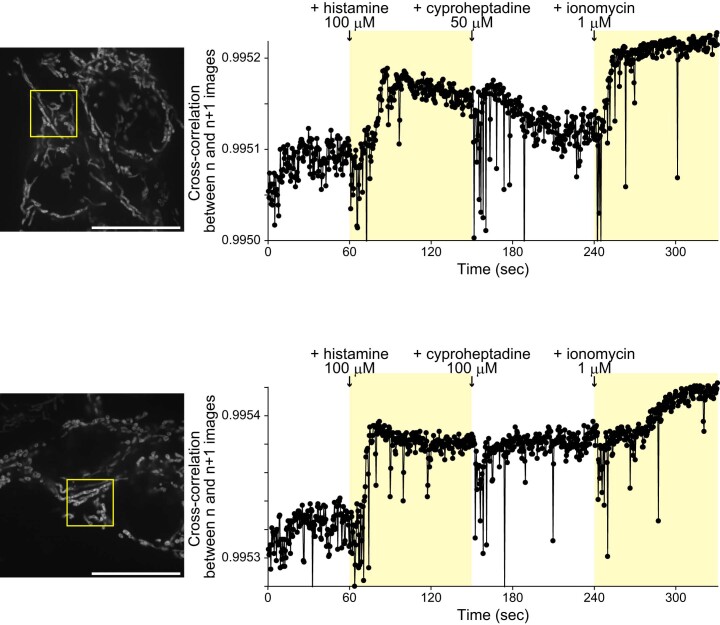
Agonist-, antagonist-, and Ca^2+^ ionophore-induced longitudinal changes in IMM structures revealed by fast, sustained, wide imaging. HeLa cells expressing COX8a=mStayGold were continuously imaged by SDSRM (SpinSR10) at a temporal resolution of 2.5 frames per second. Two representative experimental data are shown. Histamine, cyproheptadine, and ionomycin were applied at 1 min, 2.5 min, and 4 min, respectively. The time zones of Ca^2+^ mobilization are shaded. IMM rearrangements were quantified by normalized cross-correlation (https://typeset.io/papers/fast-normalized-cross-correlation-1u76lu073u). Normalized cross-correlation was calculated between *n* and *n* + 1 images in individual pixels. Calculated values were averaged in each image and plotted as a function of time. Regions indicated by yellow boxes are zoomed-in for movie presentation (Supplementary Video 12). Scale bars, 10 µm. Shown are two representatives of *n* = 12 independent experiments (transfections) that observed histamine- and ionomycin-induced decreases in the mobility of IMM structures. Source data

To aim for sustained but simple live imaging of IMMs, we fused mStayGold to the C terminus of subunit CoxVIIIa of cytochrome *c* oxidase. After the construct (COX8a=mStayGold) was transfected into HeLa cells, substantial cell-to-cell variation in the expression level was observed. Among transfectants, we chose cells with modestly labeled mitochondria for continuous observations (Supplementary Note 5). We first used the conventional lattice SIM technique to perform volumetric imaging and observed cristae membrane dynamics in a cell-wide manner (Fig. 5c and Supplementary Video 9). We next used SDSRM (SpinSR10) to perform a high-speed imaging at one *z* position at a temporal resolution of 2–4 frames s^−1^ for 5–6 min. We noticed a variety of behaviors of mitochondria and their interior structures. Some were stable (Supplementary Video 10), whereas others were extremely dynamic and complicated (Supplementary Video 11). The observation period was sufficiently long for successive administration of three drugs; histamine to initiate physiological Ca^2+^ mobilization, an antihistamine to shut it down and ionomycin to induce Ca^2+^ mobilization again (Supplementary Video 12). We found that movement of individual mitochondria was attenuated upon Ca^2+^ mobilization but that remodeling of the IMMs was constantly active (Extended Data Fig. 9). Further studies that adopt genetical or pharmacological approaches will be needed to elucidate the power source of the cristae movements.

### Photostability with strong excitation light

**Extended Data Fig. 10:**
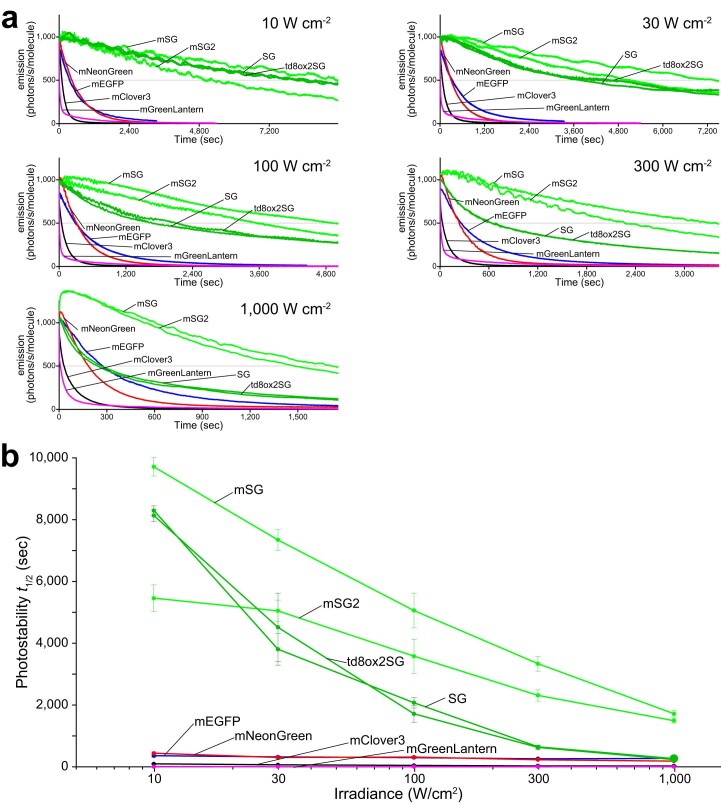
Photostability of StayGold, mStayGold, mStayGold2, and reference green-emitting FPs in live cells under continuous WF illumination with high irradiances. FPs were expressed as fusions to histone 2B (H2B) in HeLa cells and photobleached. Illumination intensities: 10, 30, 100, 300, and 1,000 W cm^−2^. StayGold is abbreviated as SG. **a**, Plot of intensity vs. normalized total exposure time, with an initial emission rate of 1,000 photons s^−1^ molecule^−1^. The curves shown are representative of three repetitions (*n* = 3 independent experiments). A photoactivation component was observed for both mStayGold and mStayGold2 as illumination intensity was increased. Signal fluctuation was due to some movements of intranuclear structures such as nucleoli. **b**, The statistical values of *t*_1/2_ (time for photobleaching from an initial emission rate of 1,000 photons s^−1^ molecule^−1^ down to 500) are plotted against the irradiances. Data points are shown as means ± s.d. (*n* = 3 independent experiments). The values are also shown in Supplementary Table 3. **a, b**, Lines are colored as follows. StayGold and td8ox2StayGold, dark green; mStayGold and mStayGoldG2, green; mEGFP, blue; mNeonGreen, red; mClover3, black; mGreenLantern, magenta. Source data

Unattenuated illumination from an arc lamp or LED lamp in conventional widefield (WF) microscopy using an objective with the highest numerical aperture (NA) provides an irradiance of up to around 10 W cm^−2^ through a common excitation bandpass filter. In our previous study, we demonstrated the superior photostability of StayGold relative to that of EGFP across the full range of light intensities (<10 W cm^−2^) of WF illumination^1^. It must be added that the typical irradiance of WF illumination in time-lapse imaging experiments with sub-second exposure times is <0.5 W cm^−2^ (refs. ^15,20^), whereas the irradiance in super-resolution SIM experiments that observe the fast dynamics of fine subcellular structures requires relatively strong excitation light (1–10 W cm^−2^). By contrast, single molecule tracking (SMT) experiments use stronger excitation light (10–1,000 W cm^−2^) depending on the spatiotemporal resolution^41^. Because the molecular fusion to mStayGold is expected to be useful in SMT, we performed photostability experiments with irradiance values of 10, 30, 100, 300 and 1,000 W cm^−2^ by using a laser-based WF microscopy system^42^. Live cells expressing H2B-FPs were continuously illuminated. The normalized photobleaching curves (Extended Data Fig. 10) and the calculated *t*_1/2_ values (Supplementary Table 3) indicate that as the irradiance was elevated to 1,000 W cm^−2^, the outstanding photostability observed at 10 W cm^−2^ was attenuated greatly for StayGold and td8ox2StayGold but only modestly for mStayGold and mStayGold2. Accordingly, mStayGold and mStayGold2 were still more than one order of magnitude more photostable at >100 W cm^−2^ than any of the other reference green-emitting FPs and would therefore meet the expectations of researchers who adopt SMT approaches.

The high photostability of mStayGold and mStayGold2 with very high irradiances has encouraged us to examine their performance in single-beam LSCM, which produces an intermittent and instantaneously strong illumination. It has been argued that the photobleaching efficiency of FPs under focused laser illumination is dependent nonlinearly on a variety of factors and is thus hardly evaluable^14,15^. As StayGold was suggested to be somehow vulnerable to light from single-beam LSCM^1^, its photobleaching behavior tends to vary with the setting. As just one setting suitable for conventional high-resolution imaging, we employed an Evident FV3000 system equipped with a ×40 objective with NA 0.95 and a 488-nm laser (Supplementary Note 6). With a laser power that provided very bright signals to all the tested H2B fusions, mStayGold and mStayGold2 produced two times more sustained fluorescence than StayGold and td8ox2StayGold, whereas the four reference green-emitting FPs photobleached rather quickly (Supplementary Fig. 19 and Supplementary Note 7).

## Discussion

To accomplish the generation of td variants of StayGold, in the present study, we preserved the dimer interface and optimized both the linker and the adaptor for tandemization to maximize the dispersibility, which was quantitated by OSER and Fluoppi assays. As shown in Fig. 3b, a td construct doubled the fluorescence brightness per unit of host protein compared to a regular monomer, thereby facilitating the observation of low-copy-number targets such as the endogenous condensin I subunit (Fig. 4 and Extended Data Fig. 6); however, the doubled size of a td construct might give rise to steric hindrance. In our experience, for example, td8ox2StayGold did not fuse to COX8a for successful labeling of the IMM.

Although we found that the crystal structure of StayGold has 1.56 Å resolution, we could not fully understand the structural basis of the high photostability of this FP and hope that future photophysical studies will provide straightforward clues. This study focuses on a directed evolution of the dimeric StayGold toward useful monomers. Similar to previous FP monomerization studies^2^, we used the crystal structure of this FP to identify mutational hotspots in the dimer interface (Fig. 1). We draw a parallel between disrupting interactions at the dimer interface of StayGold without losing its high photostability and brightness being an immense challenge and the difficulty of expanding in all directions of a triangle that has photostability, brightness and monomericity/dispersibility of an FP at the three vertices.

Because the high-throughput screening for photostable FPs in living cells is an ambitious task^43,44^, we adopted a low-throughput approach in the present study; thus, there is still room for further improvements in photophysical and dispersive properties of the monomers. Nevertheless, the possibility of using StayGold monomers as valuable alternatives to popular bright monomeric green-emitting FPs, such as mEGFP and mNeonGreen, for molecular fusion and membrane-targeting applications warrants investigation (Supplementary Fig. 20). Moreover, we are aware that mStayGold is not only a useful monovalent tag that retains the high photostability and brightness of StayGold but also an excellent evolver that would form a better expanded triangle than we expected initially. After many rounds of rational and random mutagenesis, mStayGold has evolved in such a way as to gain higher resistance to strong excitation light in the range of 10 to 1,000 W cm^−2^ (Extended Data Fig. 10) and faster maturation than StayGold (Fig. 3 and Supplementary Fig. 9). Another monomer named mStayGold2 had the same photostability as mStayGold but was slightly dimmer than StayGold, thus eventually underscoring mStayGold’s outstanding performance.

## Methods

### Protein purification

Recombinant proteins with a polyhistidine tag at the N terminus were expressed in *Escherichia* *coli* (JM109 (DE3)). Transformed *E.* *coli* were incubated in a Luria–Bertani (LB) medium containing 0.1 mg ml^−1^ ampicillin at room temperature (RT) with gentle shaking for several days. Protein purification by Ni^2+^ affinity chromatography was performed as described previously^47^.

### In vitro spectroscopy

Absorption spectra were acquired using a spectrophotometer (U-2910, Hitachi). Fluorescence excitation and emission spectra were acquired using a fluorescence spectrophotometer (F-2500, Hitachi). Absolute fluorescence quantum yields were measured using an absolute photoluminescence quantum yield spectrometer (C9920-02, Hamamatsu Photonics). The solution for spectroscopy contained 50 mM HEPES (KOH), pH 7.4, and 150 mM KCl. Protein concentrations were measured using a Protein Assay Dye Reagent Concentrate kit (5000006, Bio-Rad) with bovine serum albumin (BSA) as the standard.

### pH titrations

Measurement was performed at RT (25 °C) immediately after pH adjustment. Fluorescence was measured at the protein concentration of 200 nM using an F-2500 fluorescence spectrophotometer (Hitachi). The following buffers were used to adjust pH:

pH 3, 50 mM glycine-HCl buffer

pH 4–5, 100 mM CH_3_COONa-CH_3_COOH buffer

pH 6, 100 mM MES (NaOH) buffer

pH 7–8, 100 mM HEPES (NaOH) buffer

pH 9–10, 100 mM glycine-NaOH buffer

pH 11, 100 mM Na_2_HPO_4_-NaOH buffer

See Supplementary Fig. 11.

### Pseudonative SDS–PAGE analysis

Non-heated protein samples were separated on 10% polyacrylamide gels as described previously^48^. The gel on a UV–VIS transilluminator was photographed by iPad through a filter for GFP observation. Photoshop CS5 v.12.1 was used to crop the original photo.

### Gene construction for bicistronic expression in mammalian cells

The T2A^49^ gene was synthesized with 5′-*Hin*dIII and 3′-*Eco*RI sites and the restricted product was cloned into the *Hin*dIII/*Eco*RI sites of pBlueScript (pBS) to generate pBS/T2A. The mCherry gene was amplified using primers containing 5′-*Xho*I and 3′-*Hin*dIII sites and the restricted product was cloned in frame into the *Xho*I/*Hin*dIII sites of pBS/T2A to generate pBS/mCherry-T2A. The green-emitting FP (EGFP, mGreenLantern, StayGold, td5StayGold, td5oxStayGold, td8oxStayGold, td8ox2StayGold, QC2-6 FIQ or QC2-6(PT)) gene was amplified using primers containing 5′-*Bam*HI and 3′-*Xba*I sites and the restricted product was cloned in frame into the *Bam*HI/*Xba*I sites of pBS/mCherry-T2A to generate pBS/mCherry-T2A-green-emitting FP. Last, *Xho*I/*Xba*I fragments encoding mCherry-T2A-green-emitting FP were subcloned into pCSII-EF to generate pCSII-EF/mCherry-T2A-green-emitting FP plasmids.

### Cellular brightness assay

HeLa cells were seeded into 24-well glass-bottom plates (5826-024, IWAKI) and maintained in growth medium (Dulbecco’s modified Eagle medium (DMEM) low glucose, supplemented with 10% fetal bovine serum (FBS)). On the following day, cells were transfected with 0.5 μg pCSII-EF/mCherry-T2A green-emitting FP per well using 1 µl Lipofectamine 2000 (52887, Thermo Fisher). Forty-eight hours after transfection, cells were imaged on an inverted microscope (IX-83, Evident) equipped with an LED light bulb (X-Cite XYLIS, Excelitas Technologies), an objective lens (UPlanXApo ×4/0.16 NA, Evident) and a scientific CMOS camera (ORCA-Fusion, Hamamatsu Photonics). Green-emitting FPs were observed using a filter cube (U-FBNA, Evident), which is composed of an excitation filter (470–495 nm), a dichroic mirror (505LP) and an emission filter (510–550 nm). Thus, the central wavelength of the excitation passband was approximately 483 nm. mCherry was observed using a filter cube (U-FMCHE, Evident), which is composed of an excitation filter (565–585 nm), a dichroic mirror (595LP) and an emission filter (600–690 nm). The green-emitting FP fluorescence was corrected for the mCherry fluorescence and spectral throughput (Supplementary Fig. 7). The value was normalized to that of StayGold (Supplementary Table 2 and Fig. 3b).

### FP maturation in mammalian cells

HeLa cells were seeded into six-well plates (353046, CORNING) and maintained in growth medium (DMEM low glucose, supplemented with 10% FBS). On the following day, cells were transfected with 1 µg pCSII-EF/mCherry-T2A-green-emitting FP per well using 2 µl Lipofectamine 2000 (52887, Thermo Fisher). After a 1-h incubation with the transfection complexes, the medium was replaced with fresh phenol-red-free DMEM (044-33555, Fuji Film) supplemented with 10% FBS and GlutaMax (35050061, Thermo Fisher). One hour after the removal of the transfection complexes, cells were subjected to long-term, time-lapse imaging using a fully automated imaging system (SARTORIUS, Incucyte SX5) that was maintained at 37 °C in a 5% CO_2_ environment in an incubator (Thermo Fisher, Forma Steri-Cycle i250). Fluorescence and phase-contrast images (four images per well per channel) were acquired every 30 min using a ×10 objective lens and the G/O/NIR Filter Set. Green-emitting FPs were observed using the G channel (excitation, 453–485 nm; emission, 494–533 nm). mCherry was observed using the O channel (excitation, 546–568 nm; emission, 576–639 nm). FP signals were defined as pixels having signal values exceeding five times the s.d. above the mean fluorescence intensity of the first images. Because the fluorescence development of most of the green-emitting FPs preceded that of mCherry, the green:red ratios increased abruptly in the early phase. Therefore, each signal of a green-emitting FP was divided by the respective mCherry signal at 48 h. Last, the ratio value was corrected for the spectral throughputs of the green-emitting FPs (Supplementary Fig. 8 and Fig. 3c).

### FP maturation in bacterial cells

A homemade fluorescence analyzing system consisting of a Xenon light source MAX-302 (Asahi Spectra), an excitation filter (465–495 nm) (480AF30, Omega Optical), an emission filter (530–550 nm) (PB0540/020, Asahi Spectra) and a sCMOS camera ZYLA-5.5-USB3 (Andor) was used for time-lapse imaging of transformed *E.* *coli* colonies that expressed SG, mSG or mSG2. The whole system was controlled by MetaMorph software (Molecular Devices). Multiple colonies were made for each FP by spotting 1.5-μl drops of transformed competent JM109(DE3) cell suspension on an LB agar plate with 100 µg ml^−1^ ampicillin. After a 2-h incubation at 37 °C, the plate was placed in a stage-top incubation chamber (Tokai Hit) kept at 37 °C and time-lapse imaging was immediately started (Supplementary Fig. 9a). To evaluate the oxygen-dependent chromophore maturation, bacterial colonies on LB agar plates with 100 µg ml^−1^ ampicillin were grown in an anaerobic 2.5-l rectangular jar (Mitsubishi Gas Chemical) with an O_2_-absorbing agent AnaeroPack (Mitsubishi Gas Chemical) overnight at 37 °C. Immediately after exposure to air, time-lapse imaging was started on a 37 °C preheated plate (Tokai Hit) (Supplementary Fig. 9b and Supplementary Video 1). Images were analyzed using ImageJ (National Institutes of Health). The green-emitting FP fluorescence was corrected for the spectral throughput (Supplementary Fig. 9c).

### Spectral throughput calculation

For the calculation of relative excitation efficiency (Ex.), the ratio of the excitation intensity relative to the maximum was averaged in the bandpass. Relative emission detection efficiency (Em.) was calculated as the ratio of the emission passing the bandpass relative to the entire integrated emission (Supplementary Figs. 7–9c).

### OSER assay

The cDNA fragment encoding CytERM^4^ was synthesized according to the sequence information of Emerald-CytERM-N-17 (Addgene, #56290) with 5′-*Hin*dIII and 3′-*Bam*HI sites. As the CytERM gene has *Bam*HI, *Eco*RI and *Hin*dIII sites internally, all these sites were eliminated in the synthesis. The FP gene was amplified using primers containing 5′-*Bam*HI and 3′-*Xho*I sites. The restricted products were cloned into the *Hin*dIII/*Xho*I sites of pcDNA3 to generate pcDNA3/CytERM-FP. Twenty hours after transfection, HeLa cells on a standard 35-mm glass-bottom dish were incubated in Hanks’ Balanced Salt Solution (HBSS; 14025, Thermo Fisher Scientific) containing 15 mM HEPES-NaOH (pH 7.4) and imaged on an inverted microscope (IX-83, Evident) equipped with a ×20 objective lens (UPlanXApo ×20/0.8 NA, Evident) and a camera (ORCA-Fusion, Hamamatsu Photonics). At an *xy* position, nine images were serially acquired with a *z* step size of 0.59 µm, from which an in-focus image was mathematically generated by the extended focus imaging function of the cellSens Dimension (Evident) software (v.3.2). A logarithmic transformation was applied to all image data that had a wide range of fluorescence intensity distributions. The number of transfected cells showing whorl structures was counted. In addition, the number of transfected cells avoiding whorl formation was counted. Three independent experiments were carried out for each construct (Extended Data Fig. 1).

### Fluoppi assay

The FP gene was amplified using primers containing 5′-*Bam*HI and 3′-*Eco*RI sites and the restricted product was cloned into the *Bam*HI/*Eco*RI sites of pAsh-MCL (Medical Biological Laboratory) to generate a plasmid DNA for expression of PB1-FP. Also, the FP gene was amplified using primers containing 5′-*Bam*HI and 3′-*Xho*I sites and the restricted product was cloned into the *Bam*HI/*Xho*I sites of pAsh-MNL (Medical Biological Laboratory) to generate plasmid DNA for expression of FP-PB1. Twenty-four hours after transfection, HeLa cells on a standard 35-mm glass-bottom dish or a 24-well glass-bottom plate (5826-024, IWAKI) were incubated in HBSS (14025, Thermo Fisher Scientific) containing 10 mM HEPES-NaOH (pH 7.4) and imaged on an inverted microscope (IX-83, Evident) equipped with a ×20 objective lens (UPlanXApo ×20/0.8 NA, Evident) and a camera (ORCA-Fusion, Hamamatsu Photonics). The mirror units used for imaging green-, yellow- and red-emitting FPs were U-FBNA, U-FYFP and U-FGNA (Evident), respectively. A logarithmic transformation was applied to all image data that had a wide range of fluorescence intensity distributions (Extended Data Figs. 2 and 5).

### Expression and purification for crystallography

StayGold, in a pET-47b(+) vector (Novagen) carrying ampicillin resistance and an HRV 3C-cleavable N-terminal polyhistidine tag, was expressed in *E.* *coli* (BL21(DE3)). Transformed *E.* *coli* was incubated at 25 °C in an LB medium containing 20 μg ml^−1^ kanamycin with gentle shaking (63 r.p.m.) for 5 d. Protein purification by Co^2+^ affinity chromatography was performed using TALON resins (Clontech). Cleavage of the polyhistidine tag was performed during dialysis into 50 mM Tris-HCl (pH 7.5), 0.3 M NaCl and 1 mM dithiothreitol using HRV 3 C protease at 4 °C overnight. The sample was loaded onto TALON resins and the unbound fraction was applied to a HiPrep 16/60 Sephacryl S300 HR column (cytiva) equilibrated with 20 mM HEPES-NaOH (pH 7.5) and 0.15 M NaCl for preparative separation of StayGold. Finally, the untagged product was concentrated to 8.3 mg ml^−1^ using Amicon Ultra (3,000 MW cutoff) (Merck).

### Crystallization and X-ray data collection

Crystals of StayGold were grown at 20 °C using the sitting-drop vapor diffusion method by mixing 0.1 μl protein solution (8.3 mg ml^−1^ in 20 mM HEPES-NaOH (pH 7.5) and 0.15 M NaCl) with 0.1 μl reservoir solution I (25% (w/v) PEG3350, 0.2 M MgCl_2_ and 0.1 M Tris-HCl, pH 8.5) or reservoir solution II (20% (w/v) PEG4000, 20% (v/v) 2-propanol and 0.1 M sodium citrate, pH 5.6). The mixture was sealed over a well containing 50 μl reservoir I or reservoir II solution, respectively. Individual crystals were soaked in 1 ml reservoir I or reservoir II solution containing 250 mg trehalose, scooped using a nylon loop and flush-cooled in liquid nitrogen. The diffraction data were collected at 100 K using the BL26B2 beam line at the SPring-8 and were processed using the DIALS program^50^.

### Structure determination and refinement

The structure of StayGold was determined by the molecular replacement technique with a model of GFP (PDB, 2Q57) as a search model using phenix.phaser^51^. The model was refined using phenix.refine^52^ and repeatedly corrected using Coot^53^. Refinement statistics of structures are summarized in Supplementary Table 1. Structural figures were prepared using PyMOL.

### Gene construction (nuclear targeting)

The mouse H2B gene (Fantom3) was amplified using primers containing 5′-*Xho*I and 3′-*Hin*dIII sites and the restricted product was cloned into the *Xho*I/*Hin*dIII sites of pBS Coupler 1 (ref. ^54^) to generate pBS Coupler 1/H2B. In addition, the green-emitting FP (EGFP, mEGFP, mClover3, mNeonGreen, mGreenLantern, StayGold, tdStayGold, td5StayGold, td5oxStayGold, td8oxStayGold, td8ox2StayGold, QC2-6 FIQ or QC2-6(PT)) gene was amplified using primers containing 5′-*Bam*HI and 3′-*Xba*I sites and the restricted product was cloned in frame into the *Bam*HI/*Xba*I sites of pBS Coupler 1/H2B=FP. Last, *Xho*I/*Xba*I fragments encoding H2B=FPs were subcloned into pCSII-EF for transfection.

### WF photobleaching

Living cells on 35-mm glass-bottom dishes were incubated in HBSS containing 15 mM HEPES-NaOH (pH 7.4) and imaged on an inverted microscope (IX-83, Evident) equipped with an LED light bulb (X-Cite XYLIS, Excelitas Technologies), a ×60 objective lens (UPlanSApo ×60/1.35 NA) and a scientific CMOS camera (ORCA-Fusion, Hamamatsu Photonics). The data were analyzed using Excel (2019). The fluorescence intensity at *t* = 0 was normalized to 1,000 photons s^−1^ per molecule and the time axis was adjusted according to the standard method^15^. The power of excitation light (W) above the objective at the focal plane was measured using a microscope slide power meter sensor (S170C; Thorlabs) and an optical power and energy meter (PM100D; Thorlabs). The power was divided by the area of the illumination field (cm^2^) to obtain the irradiance. In all cases of WF microscopy, the illuminator (collimator lens) was adjusted to achieve Köhler illumination. A color acrylic plate (Tokyu Hands) was placed at the focal plane to evaluate illumination uniformity on a CCD (CMOS) image (Fig. 2 and Supplementary Fig. 6).

### WF photobleaching with high irradiances

Living cells on 35-mm glass-bottom dishes were incubated in HBSS containing 15 mM HEPES-NaOH (pH 7.4) and imaged on an inverted microscope (Eclipse Ti-E, Nikon) equipped with a 200-mW 491-nm laser (Calypso, Cobolt), ×100 objective lens (CFI Apo TIRF ×100/1.49 NA) and a high-speed CMOS camera (based on SA-1, Photron) coupled to an image intensifier (V8070U-74, Hamamatsu) by an optical-fiber bundle^42^. To perform image acquisitions within the full-well capacity of the CMOS sensor, the camera was operated at frame rates of 60, 250, 1,000, 3,000 and 10,000 frames s^−1^ at illumination intensities of 10, 30, 100, 300 and 1,000 W cm^−2^, respectively. The excitation laser illuminated a circular (two-dimensional Gaussian) area with a 28.5-µm radius (standard deviation) on the sample plane. Since only the central part of the illumination area was used for imaging and the intensity was reduced by less than 11% from the center to the horizontal/vertical side end of the field of view (27.1 × 27.1 µm), the irradiance was obtained by the laser density at the center (= *P*/2πσ^2^) based on the laser power measured after the objective lens (*P*) and the standard deviation of the Gaussian profile (σ)^55^. See Extended Data Fig. 10.

### Single-beam LSCM photobleaching

Living cells on 35-mm glass-bottom dishes were incubated in HBSS (14025076, Thermo Fisher Scientific) and imaged using an inverted LSCM system (FV3000; Evident) equipped with a ×40 objective lens (UPlanSApo ×40/0.95 NA). Green-emitting FPs were excited by a 488-nm diode laser and fluorescence was detected within the wavelength range of 500–600 nm. The power of excitation light (W) above the objective at the focal plane was measured using a microscope slide power meter sensor (S170C; Thorlabs) and an optical power and energy meter (PM100D; Thorlabs). The power was divided by the area of the scanned field (cm^2^) to obtain the irradiance (Supplementary Fig. 19 and Supplementary Note 6).

### Fluorescence lifetime measurements

HeLa cells were transfected with 0.5 µg of pcDNA3/mStayGold, pcDNA3/mStayGold2 or pCSII/StayGold using 1 µl Lipofectamine 2000 (52887, Thermo Fisher). Two days after transfection, the medium was exchanged with HBSS, 14025, Thermo Fisher Scientific) containing 15 mM HEPES-NaOH (pH 7.4). Cells were imaged on an inverted laser-scanning confocal microscope (TCS SP8 STED ONE, Leica Microsystems) equipped with a ×20 objective lens (HC PL APO CS2 ×20/0.75 DRY, Leica). The FPs were excited at 488 nm by a white light laser (frequency, 80 MHz) and the emitted fluorescence (510–600 nm) was detected with the HyD SMD4 detector. The lifetime was analyzed using LAS X FLIM/FCS software, v.3.5.5 (Leica Microsystems).

### Gene construction for genome editing

pMT690-2 is a plasmid that contains a 1,231-bp genomic fragment around the termination codon of *NCAPH*^56^. A series of ‘cassette constructs’ encoding FP tags plus selection markers^57^ were provided by M.T. Kanemaki at the National Institute of Genetics in Mishima, Japan. They can be used for generating FP knock-in cells at the C-terminal end of any protein of interest via homology-directed repair. Among the constructs, pMK281 (mCherry2-Hygro) and pMK278 (mClover3-Hygro) were selected in the present study. Also, the mCherry2 gene in pMK281 was replaced with the td5oxStayGold gene to generate a new cassette construct, pMT892 (td5oxStayGold-Hygro). The td5oxStayGold-Hygro and mClover3-Hygro cassette genes were amplified using pMK892 and pMT278 as templates and the PCR products were inserted in frame via Gibson assembly (NEB) at a site immediately upstream of the termination codon of *NCAPH* in pMT690-2 to generate pMT897 and pMT899, respectively. In both constructs, the C terminus of CAP-H was linked to FP via a linker amino acid tract GSGAAS.

### Genome-edited cell lines

pMT691, a derivative of pX330 (Addgene plasmid #42230), can be used for cleaving the genome with Cas9 near the termination codon of *NCAPH*^56^. HCT116 cells were co-transfected with pMT691 and pMT897 using FuGene HD (Promega) and were then cultured in the presence of 100 μg ml^−1^ hygromycin B (Nacalai Tesque) for selection of cell clones in which CAP-H was endogenously tagged at the C terminus with td5oxStayGold. Single cell colonies growing normally and exhibiting green fluorescence in the cytoplasm in interphase cells and on chromosomes in mitotic phase cells were picked up. Among them was a cell clone designated as #897, which was further characterized for the tagging (CAP-H-td5oxStayGold). Likewise, cotransfection of HCT116 cells with pMT691 and pMT899 resulted in the generation of a cell line #899 carrying CAP-H-mClover3. Cells were cultured at 37 °C with 5% CO_2_ in DMEM supplemented with 10% FBS.

### Validation of FP integration in knock-in cell lines

First, junction PCR was performed using a forward primer 5′ outside of the left homology arm (P758, 5′-GTTAATCTCTTACTGTGCCT-3′) and a reverse primer 3′ outside of the right homology arm (P759, 5′-TCTCTTCCATTCTCCTCCGA-3′). Second, western blotting analysis was performed using a rabbit polyclonal anti-CAP-H antibody (Proteintech, 11515-1-AP, 1:1,000 dilution) and a mouse monoclonal anti-β-actin antibody (Sigma-Aldrich, A1978 clone AC-15, 1:5,000 dilution). Photoshop v.22.5.8 was used to crop original pictures and for contrast adjustment (Supplementary Fig. 15a,b).

### Gene construction (Golgi targeting)

The td5StayGold(c4) gene was amplified using primers containing 5′-*Kpn*I and 3′-*Eco*RI sites. The restricted product was substituted for the StayGold(c4) gene in pcDNA3/StayGold(c4)-20aa-Giantin^1^, which is identical to pcDNA3/StayGold(c4)=GianCreg. GianCreg is a C-terminal domain that contains amino acids 3,131–3,259 of human giantin. The resultant plasmid was pcDNA3/td5StayGold(c4)=GianCreg.

### Gene construction (N-terminal targeting of β-tubulin)

The td8ox2StayGold(c4) gene was amplified using primers containing 5′-*Bam*HI and 3′-*Eco*RI sites. Also, the β-tubulin gene was amplified using primers containing 5′-*Hin*dIII and 3′-*Xho*I sites. The two restricted products were sequentially cloned into the *Bam*HI/*Eco*RI and *Hin*dIII/*Xho*I sites of pBS Coupler 4 (ref. ^54^). Finally, the *Bam*HI/*Xho*I fragment was cloned into pcDNA3 to generate pcDNA3/td8ox2StayGold(c4)=β-tubulin. The mStayGold(c4) gene was amplified using primers containing 5′-*Bam*HI and 3′-*Eco*RI sites. The restricted product was cloned into the *Bam*HI/*Eco*RI sites of pBS Coupler 4 (ref. ^54^). Also, the β-tubulin gene was cloned into the *Hin*dIII/*Xho*I sites. Finally, the *Bam*HI/*Xho*I fragment was cloned into pcDNA3 to generate pcDNA3/mStayGold(c4)=β-tubulin.

### Gene construction (C-terminal targeting of β-tubulin)

The mStayGold gene was amplified using primers containing 5′-*Hin*dIII and 3′-*Xho*I sites and the β-tubulin gene was amplified using primers containing 5′-*Not*I and 3′-*Spe*I sites. The two restricted products were sequentially cloned into the *Hin*dIII/*Xho*I and *Not*I/*Spe*I sites of pBS Coupler 4 (ref. ^54^). The resultant plasmid provided a *Not*I/*Xho*I fragment, which was cloned into pcDNA3 to generate pcDNA3/β-tubulin=mStayGold.

### Stable transformants

Replication-defective, self-inactivating lentiviral vectors were used^1^. The pCSII-EF-MCS vector encoding td5StayGold(c4)=GianCreg or COX8a=mStayGold was co-transfected with the packaging plasmid (pCAG-HIVgp) and the VSV-G-/Rev-expressing plasmid (pCMV-VSV-G-RSV-Rev) into 293T cells. High-titer viral solutions were prepared and used for transduction into HeLa cells (multiplicity of infection of 1–10). Most (>95%) of the resultant cells uniformly exhibited green fluorescence and were used as stable transformants.

### Immunocytochemistry of the Golgi apparatus

After being washed in phosphate-buffered saline (PBS) three times, cells stably expressing td5StayGold(c4)=GianCreg were chemically fixed (see below) and then incubated in blocking solution (PBS containing 3% BSA and 0.1% Triton X-100) for 60 min at RT. The cells were then reacted with primary antibodies (Abs) in blocking solution at RT for 60 min. After being washed in PBS-T (PBS containing 0.1% Triton X-100) three times, the cells were reacted with secondary Abs in blocking solution at RT for 60 min. After the cells were washed in PBS-T three times, nuclear staining was performed using DAPI (Fuji Film, 340-07971, 1:1,000 dilution) at RT in PBS for 5 min. The fixation conditions and used Abs are as follows.

<GM130>

Fixation: 4% paraformaldehyde (PFA)/PBS at RT for 5 min.

Primary Ab: anti-GM130 Ab (MBL, PM061), 1:250 dilution.

Secondary Ab: donkey anti-rabbit IgG (H + L) highly cross-adsorbed secondary Ab, Alexa Fluor 647-conjugated (Thermo Fisher, A-31573), 1:500 dilution.

<Giantin>

Fixation: 4% PFA/PBS at RT for 5 min.

Primary Ab: anti-giantin Ab (PROTEINTECH, 22270-1-AP), 1:250 dilution.

Secondary Ab: donkey anti-rabbit IgG (H + L) highly cross-adsorbed secondary Ab, Alexa Fluor 647-conjugated (Thermo Fisher, A-31573), 1:500 dilution.

<TGN46>

Fixation: 4% PFA + 0.05% glutaraldehyde/PBS at RT for 5 min.

Primary Ab: anti-TGN46 Ab (Sigma-Aldrich, SAB4200355), 1:100 dilution.

Secondary Ab: donkey anti-mouse IgG (H + L) highly cross-adsorbed secondary Ab, Alexa Fluor 647-conjugated (Thermo Fisher, A-31571), 1:500 dilution.

Cell samples were imaged using an inverted LSCM system (FV3000, Evident) equipped with a ×60 water objective lens (UPlanSApo ×60/1.2 NA). The size of the confocal aperture was 1 Airy disk. For a zoom factor of 4× and a pixel array size of 512 × 512, the size of each pixel was calculated to be 0.104 μm. Confocal images were acquired every 0.52 μm along the *z* axis to create *z* stacks (25–27 slices) that covered the entire Golgi apparatus.

td5StayGold, Alexa 647 and DAPI were excited at 488 nm, 640 nm and 405 nm, respectively, using a dichroic mirror (DM405/488/561/640). Their fluorescence signals were acquired sequentially in each scanning line. A scatter-plot was generated between td5StayGold(c4)=GianCreg and GM130, giantin or TGN46 in each *z* slice and the plots across the *z* range were merged. On the basis of the Otsu method^58^, threshold values were automatically optimized for the fluorescence of td5StayGold and Alexa Fluor 647 using Fiji (fiji.sc). After exclusion of data points below the thresholds in both colors and data points showing signal saturation, colocalization was quantified by correlation analysis. The Pearson correlation coefficient (*r*) was determined using R (www.r-project.org). Three independent experiments (different immunostaining experiments) were carried out for each combination (Extended Data Fig. 7).

### Cytochemistry of the Golgi apparatus

HeLa cells were fixed 2 d after transfection with cDNA of td5StayGold(c4)=GianCreg. Cell samples were imaged using an inverted LSCM system (FV3000, Evident) equipped with a ×60 water objective lens (UPlanSApo ×60/1.2 NA). The size of the confocal aperture was 1 Airy disk. For a zoom factor of 1× and a pixel array size of 2,048 × 2,048, the size of each pixel was calculated to be 0.104 μm. Confocal images were acquired every 1.0 μm along the *z* axis to create *z* stacks (20 slices) that covered the entire Golgi apparatus. td5StayGold and DAPI were excited at 488 and 405 nm, respectively, using a dichroic mirror (DM405/488/561/640) (Fig. 5a).

### Colocalization of Lifeact-mStayGold and F-tractin-mScarlet-I

Vero cells were co-transfected with the two plasmids. Cells were imaged live using an inverted LSCM system (TCS SP8, Leica) equipped with a ×93 objective lens (HC PL APO ×93/1.30 GLYC motCORR objective lens). The pinhole size was 132 nm (back-projected size) and the size of each pixel was calculated to be 30 nm (Supplementary Fig. 17).

### Gene construction (inner mitochondrial membrane targeting)

The mouse COX8a cDNA was amplified using primers containing 5′-*Bam*HI and 3′-*Eco*RI sites and the restricted product was cloned into the *Bam*HI/*Eco*RI sites of pBS Coupler 4 (ref. ^54^) to generate pBS Coupler 4/COX8a. The mStayGold gene was amplified using primers containing 5′-*Hin*dIII and 3v-*Xho*I sites and the restricted product was cloned in frame into the *Hin*dIII/*Xho*I sites of pBS Coupler 4/COX8a to generate pBS Coupler 4/COX8a=mStayGold. Last, the *BamH*I/*Xho*I fragment encoding COX8a=mStayGold was subcloned into pcDNA3 for transfection.

### Gene construction (F-actin targeting, F-tractin)

The rat F-tractin cDNA that corresponds to an N-terminal domain consisting of 41 amino acids was amplified using primers containing 5′-*Kpn*I and 3′-*Xho*I sites and the restricted product was cloned into the *Kpn*I/*Xho*I sites of pBS Coupler 1 (ref. ^54^) to generate pBS Coupler 1/F-tractin. The mStayGold gene was amplified using primers containing 5′-*Bam*HI and 3′-*Not*I sites and the restricted product was cloned in frame into the *Bam*HI/*Not*I sites of pBS Coupler 1/F-tractin to generate pBS Coupler 1/F-tractin=mStayGold. Last, the *Kpn*I/*Not*I fragment encoding F-tractin=mStayGold was subcloned into pcDNA3 for transfection.

### Gene construction (F-actin targeting, utrophin)

The human utrophin cDNA that corresponds to an N-terminal domain consisting of 261 amino acids was amplified using primers containing 5′-*Bam*HI and 3′-*Not*I sites and the restricted product was cloned into the *Bam*HI/*Not*I sites of pBS Coupler 1 (ref. ^54^) to generate pBS Coupler 1/UtrCH. The mStayGold(c4) gene was amplified using primers containing 5′-*Kpn*I and 3′-*Xho*I sites and the restricted product was cloned in frame into the *Kpn*I/*Xho*I sites of pBS Coupler 1/UtrCH to generate pBS Coupler 1/mStayGold(c4)=UtrCH. Last, the *Kpn*I/*Not*I fragment encoding mStayGold(c4)=UtrCH was subcloned into pcDNA3 for transfection.

### Gene construction (F-actin targeting, Lifeact)

The mStayGold gene was amplified using primers containing 5′-*Bam*HI and 3′-*Not*I sites and the restricted product was substituted for the mCherry gene at the *Bam*HI/*Not*I sites of mCherry-Lifeact-7 (#54491, Addgene).

### Lattice SIM for live imaging

Super-resolution 3D SIM images were acquired continuously on a ZEISS Elyra 7 equipped with a PlanApo ×63/1.46 NA oil immersion objective at 37 °C. The Leap mode for lattice SIM was used to increase the temporal resolution of volumetric imaging. Image analysis was carried out with ZEN 2014 (v.9.1) (Fig. 5c, Supplementary Fig. 16 and Supplementary Video 9).

### SpinSR10

Living cells on 35-mm glass-bottom dishes in HBSS containing 15 mM HEPES-NaOH (pH 7.4) were imaged using a SpinSR10 imaging system (Evident) built on an Evident inverted microscope (IX83P2ZF) equipped with an ORCA-Flash 4.0 V3 camera (Hamamatsu Photonics), a motorized stage (IX3-SSU) and a ×100 oil objective lens (UPLAPO ×100 OHR, NA 1.50). With the SoRa spinning disk, the optical resolution in an *xy* plane at 488 nm excitation is approximately 160 nm. The total magnification of the system was considered to determine the best sampling interval of the camera (pixel binning).

#### Spinning-disk super-resolution microscopy

A ×3.2 magnification changer was used for observing the Golgi apparatus (Fig. 5b and Supplementary Videos 5 and 6), cytoskeletons (Extended Data Fig. 8, Supplementary Fig. 18 and Supplementary Videos 7 and 8) and IMM (Extended Data Fig. 9, Supplementary Fig. 20a and Supplementary Videos 10–12). Among these, the following figures and videos were processed with deconvolution using a commercial algorithm ‘Olympus Super Resolution’ to achieve super-resolution imaging (Extended Data Figs. 8 and 9; Supplementary Figs. 18 and 20b–d and Supplementary Videos 7, 8 and 10–12).

#### Spinning-disk laser-scanning confocal microscopy

The ×3.2 magnification changer was not used for confocal imaging of condensin I (Fig. 4, Extended Data Fig. 6 and Supplementary Videos 2–4).

When single-plane images were acquired rapidly, the autofocus function of a *z*-drift compensator (IX3-ZDC2, Evident) was set continuously active. Image acquisition and analysis were carried out using the Evident cellSens software (v.3.1.1).

### Analysis of rapid motion of IMM structures

Normalized cross-correlation was calculated between *n* and *n* + 1 images in individual pixels. The calculation was performed using a customized program (Extended Data Fig. 9).

### Reporting summary

Further information on research design is available in the Nature Portfolio Reporting Summary linked to this article.

## Online content

Any methods, additional references, Nature Portfolio reporting summaries, source data, extended data, supplementary information, acknowledgements, peer review information; details of author contributions and competing interests; and statements of data and code availability are available at 10.1038/s41592-023-02085-6.

### Supplementary information

Supplementary Information Supplementary Notes 1–8, Figs. 1–20, Tables 1–3, Video Captions 1–12 and References.

Reporting Summary

Supplementary Video 1 **Visualizing the oxygen-dependent development of fluorescence from colonies of JM109(DE3) cells expressing mSG, mSG2 or SG, which were fully grown under a strict anaerobic condition on the agar plate**. See Supplementary Fig. 9b.

Supplementary Video 2 **Visualization of chromosome targeting of td5oxStayGold-tagged condensin I at low copy number expressed via a genome-editing technique**. After release from cell cycle arrest, genome-edited HCT116 cells (#897) were imaged for CAP-H-td5oxStayGold (at 488 nm excitation) and SiR-DNA-labeled chromosomes (at 637 nm excitation) using spinning-disk LSCM (SpinSR10) at the indicated times (hour: min). Every 1 min, 3D scanning was executed with a *z*-step size of 1 µm over an axial range of 13 µm, and the green and far-red fluorescence images were merged. MIP images are shown. This video (6.20 MB) has been generated via considerable compression of the original large-volume video data (1.52 GB). Compression was made using TMPGEnc. See Fig. 4. Shown is a representative of *n* = 3 independent experiments.

Supplementary Video 3 **High-speed visualization of chromosome targeting of td5oxStayGold-tagged condensin I in genome-edited HCT116 cells**. After release from cell cycle arrest, genome-edited HCT116 cells (#897) were imaged at a single *z* position for observing CAP-H-td5oxStayGold (488 nm excitation) and for SiR-DNA-labeled chromosomes (637 nm excitation) by spinning-disk LSCM (SpinSR10) at one frame s^−1^. Merged images at the indicated times (min: s). This video (9.05 MB) has been generated via considerable compression of the original large-volume video data (7.03 GB). Compression was made using FFmpeg. See Extended Data Fig. 6a.

Supplementary Video 4 **Photostability comparison between CAP-H-mClover3 and CAP-H-td5oxStayGold under the same optical conditions**. Genome-edited HCT116 cells (#899, left versus #897, right) during prometaphase were volume (*z* step, 0.25 µm; *z* range, 2.5 µm) imaged by spinning-disk LSCM (SpinSR10) with excitation at 488 nm continuously every 6.9 s over a total period of 278 s. MIP images are shown. This video (1.40 MB) has been generated via considerable compression of the original large-volume video data (12.4 MB). Compression was made using TMPGEnc. Elapsed times (min: s). See Extended Data Fig. 6b.

Supplementary Video 5 **Visualization of td5StayGold-harboring Golgi membranes**. Volumetric and continuous imaging of HeLa cells expressing td5StayGold(c4)=GianCreg in two independent experiments (top and bottom). Cells were volume (*z* step, 0.5 µm; *z* range, 2.5 µm) imaged by SDSRM (SpinSR10) continuously with an exposure time of 100 ms without using the *z*-drift compensator (IX3-ZDC2, Evident). MIP images are shown. This video (9.88 MB) has been generated via considerable compression of the original large-volume video data (122 MB). Compression was made using TMPGEnc. Elapsed times (min: s). See Fig. 5b.

Supplementary Video 6 **Visualization of the Golgi apparatus and microtubule network**. A COS-7 cell expressing td5StayGold(c4)=GianCreg and td8ox2StayGold(c4)=β-tubulin was volume (*z* step, 0.5 µm; *z* range, 1.5 µm) imaged by SDSRM (SpinSR10) continuously with an exposure time of 200 ms without using the *z*-drift compensator (IX3-ZDC2, Evident). MIP images are shown. This video (9.43 MB) has been generated via considerable compression of the original large-volume video data (100 MB). Compression was made using TMPGEnc. Elapsed times (min: s).

Supplementary Video 7 **Visualizing F-actin dynamics by continuous, sustainable, cell-wide imaging**. COS-7 cells expressing F-tractin=mStayGold (left) or mStayGold(c4)=UtrCH by imaged by SDSRM (SpinSR10) at a single *z* position at 3.18 frames s^−1^ for 5.24 min. Elapsed times (min: s). This video (9.05 MB) has been generated via considerable compression of the original large-volume video data (7.03 GB). Compression was made using TMPGEnc. See Supplementary Fig. 18.

Supplementary Video 8 **Visualizing the effects of drugs on F-actin organization**. COS-7 cells expressing F-tractin=mStayGold (top) or mStayGld(c4)=UtrCH (bottom) were imaged by SDSRM (SpinSR10) at a single *z* position at 2.41 frames s^−1^ for 13.82 min. Cells were treated with 1 µM cytochalasin D (left) or 2 µM latrunculin A (right). Elapsed times (min: s). This video (9.38 MB) has been generated via considerable compression of the original large-volume video data (4.29 GB). Compression was made using FFmpeg. See Extended Data Fig. 8.

Supplementary Video 9 **Visualizing the inner mitochondrial membrane dynamics by sustainable, cell-wide volumetric SIM imaging**. HeLa cells expressing COX8a=mStayGold were 3D scanned continuously with a *z*-step size of 0.11 µm over an axial range of 2.08 µm by lattice SIM (Elyra 7) at 37 °C. The total number of acquired volumes was 47. SIM^2^ was used for image reconstruction. This video (6.69 MB) has been generated via considerable compression of the original large-volume video data (47 MB). Compression was made using TMPGEnc. Elapsed times (min: s). See Fig. 5c.

Supplementary Video 10 **Visualizing the inner mitochondrial membrane dynamics by fast, sustainable imaging**. HeLa cells expressing COX8a=mStayGold were imaged by SDSRM (SpinSR10). Single-plane images were acquired continuously at 8.70 frames s^−1^ (exposure time: 100 ms). This video highlights stable mitochondria. The autofocus function of a *z*-drift compensator (IX3-ZDC2, Evident) was continuously active. The total number of acquired frames was 1,000. This video (6.71 MB) has been generated via considerable compression of the original large-volume video data (70.7 MB). Compression was made using TMPGEnc. Elapsed times (min: s). The stable IMM dynamics shown is a representative of *n* = 17 cells over *n* = 10 independent transfections.

Supplementary Video 11 **Visualizing the inner mitochondrial membrane dynamics by fast, sustainable imaging**. HeLa cells expressing COX8a=mStayGold were imaged by SDSRM (SpinSR10). Single-plane images were acquired continuously at 2.41 frames s^−1^ (exposure time: 400 ms). This video highlights a mobile mitochondrium. The autofocus function of a *z*-drift compensator (IX3-ZDC2, Evident) was continuously active. The total number of acquired frames was 500. This video (3.19 MB) has been generated via considerable compression of the original large-volume video data (31.4 MB). Compression was made using TMPGEnc. Elapsed times (min: s). The mobile IMM dynamics shown is a representative of *n* = 14 cells over *n* = 9 independent transfections.

Supplementary Video 12 **Agonist-, antagonist-, and Ca**^**2+**^ **ionophore-induced longitudinal changes in IMM structures revealed by fast, sustained, wide imaging**. HeLa cells expressing COX8a=mStayGold were imaged by SDSRM (SpinSR10) continuously at a temporal resolution of 2.5 frames s^−1^. Two representative experimental data are shown. Histamine, cyproheptadine and ionomycin were applied at 1 min, 2.5 min and 4 min, respectively. The autofocus function of a *z*-drift compensator (IX3-ZDC2, Evident) was continuously active. This video (8.9 MB) has been generated via considerable compression of the original large-volume video data (412 MB). Compression was made using TMPGEnc. Elapsed times (min: s). See Extended Data Fig. 9. Representatives of *n* = 12 independent experiments (transfections).

### Source data

Source Data Fig. 2 Numerical source data.

Source Data Fig. 3 Numerical source data.

Source Data Extended Data Fig. 1 Numerical source data.

Source Data Extended Data Fig. 2 Numerical source data.

Source Data Extended Data Fig. 5 Numerical source data.

Source Data Extended Data Fig. 7 Numerical source data.

Source Data Extended Data Fig. 9 Numerical source data.

Source Data Extended Data Fig. 10 Numerical source data.

## Data Availability

The accession numbers in the DDBJ/GenBank databases are LC756333 for mStayGold (QC2-6 FIQ), LC756334 for mStayGold2 (QC2-6(PT)), LC756335 for td5StayGold, LC756336 for td5oxStayGold and LC756337 for td8ox2StayGold. The entry IDs in the Protein Data Bank are 8ILK and 8ILL for atomic structures of StayGold crystallized at pH 8.5 and pH 5.6, respectively. All data generated in this study are available through the RIKEN Research Data and Copyrighted-work Management System (dmsgrdm.riken.jp/egdq4/). Plasmid DNAs containing mStayGold or tdStayGold variants are available from the RIKEN Bio-Resource Center (en.brc.riken.jp) under a material transfer agreement with RIKEN. Source data are provided with this paper.

## Code availability

The custom-made software source code for ‘quantification of rapid motion of IMM structures’ (Extended Data Fig. 9) is available through the R2DMS (dmsgrdm.riken.jp/egdq4/).

## References

[1] Hirano M (2022). A highly photostable and bright green fluorscent protein. Nat. Biotechnol..

[2] Campbell RE (2002). A monomeric red fluorescent protein. Proc. Natl Acad. Sci. USA.

[3] Watanabe T (2017). Genetic visualization of protein interactions harnessing liquid phase transitions. Sci. Rep..

[4] Costantini LM, Fossati M, Francolini M, Snapp EK (2012). Assessing the tendency of fluorescent proteins to oligomerize under physiological conditions. Traffic.

[5] Costantini LM (2015). A palette of fluorescent proteins optimized for diverse cellular environments. Nat. Commun..

[6] Cranfill PJ (2016). Quantitative assessment of fluorescent proteins. Nat. Methods.

[7] Gustafsson MG (2000). Surpassing the lateral resolution limit by a factor of two using structured illumination microscopy. J. Microsc..

[8] Hayashi S, Okada Y (2015). Ultrafast superresolution fluorescence imaging with spinning disk confocal microscope optics. Mol. Biol. Cell.

[9] Zacharias DA, Violin JD, Newton AC, Tsien RY (2002). Partitioning of lipid-modified monomeric GFPs into membrane microdomains of live cells. Science.

[10] Bajar BT (2016). Improving brightness and photostability of green and red fluorescent proteins for live cell imaging and FRET reporting. Sci. Rep..

[11] Shaner NC (2013). A bright monomeric green fluorescent protein derived from *Branchiostoma lanceolatum*. Nat. Methods.

[12] Campbell BC (2020). mGreenLantern: a bright monomeric fluorescent protein with rapid expression and cell filling properties for neuronal imaging. Proc. Natl Acad. Sci. USA.

[13] Shaner NC (2004). Improved monomeric red, orange and yellow fluorescent proteins derived from *Discosoma* sp. red fluorescent protein. Nat. Biotechnol..

[14] Shaner NC (2014). Fluorescent proteins for quantitative microscopy: important properties and practical evaluation. Methods Cell Biol..

[15] Shaner NC (2008). Improving the photostability of bright monomeric orange and red fluorescent proteins. Nat. Methods.

[16] Batty P, Gerlich DW (2019). Mitotic chromosome mechanics: how cells segregate their genome. Trends Cell Biol..

[17] Gerlich D, Hirota T, Koch B, Peters J-M, Ellenberg J (2006). Condensin I stabilizes chromosomes mechanically through a dynamic interaction in live cells. Curr. Biol..

[18] Takagi M (2018). Ki-67 and condensins support the integrity of mitotic chromosomes through distinct mechanisms. J. Cell Sci..

[19] Koch B (2018). Generation and validation of homozygous fluorescent knock-in cells using CRISPR-Cas9 genome editing. Nat. Protoc..

[20] Bindels DS (2017). mScarlet: a bright monomeric red fluorescent protein for cellular imaging. Nat. Methods.

[21] Frisbie CP (2019). Post-ER stress biogenesis of Golgi is governed by Giantin. Cells.

[22] Bottanelli F (2017). A novel physiological role for ARF1 in the formation of bidirectional tubules from the Golgi. Mol. Biol. Cell.

[23] Weigel AV (2021). ER-to-Golgi protein delivery through an interwoven, tubular network extending from ER. Cell.

[24] Hirschberg K (1998). Kinetic analysis of secretory protein traffic and characterization of Golgi to plasma membrane transport intermediates in living cells. J. Cell Biol..

[25] Li D (2015). Extended-resolution structured illumination imaging of endocytic and cytoskeletal dynamics. Science.

[26] Tie HC, Ludwig A, Sandin S, Lu L (2018). The spatial separation of processing and transport functions to the interior and periphery of the Golgi stack. eLife.

[27] Luzio JP (1990). Identification, sequencing and expression of an integral membrane protein of the *trans*-Golgi network (TGN38). Biochem. J..

[28] Malek M, Plessner M, Grosse R (2017). Actin visualization at a glance. J. Cell Sci..

[29] Fritzsche M (2017). Self-organizing actin patterns shape membrane architecture but not cell mechanics. Nat. Commun..

[30] Xia S (2019). Nanoscale architecture of the cortical actin cytoskeleton in embryonic stem cells. Cell Rep..

[31] Kondadi AK, Anand R, Reichert AS (2020). Cristae membrane dynamics-A paradigm change. Trends Cell Biol..

[32] Ngo J, Osto C, Villalobos F, Shirihai OS (2021). Mitochondrial heterogeneity in metabolic diseases. Biology.

[33] Stephan T, Roesch A, Riedel D, Jakobs S (2019). Live-cell STED nanoscopy of mitochondrial cristae. Sci. Rep..

[34] Kondadi, A. K. et al. Cristae undergo continous cycles of membrane remodelling in a MICOS-dependent manner. *EMBO Rep.* **21**, e49776 (2020). 10.15252/embr.201949776 PMC7054676 32067344

[35] Stephan, T. et al. MICOS assembly controls mitochondrial inner membrane remodeling and crista junction redistribution to mediate cristae formation. *EMBO J.* **39**, e104105 (2020). 10.15252/embj.2019104105 PMC7361284 32567732

[36] Wang C (2019). A photostable fluorescent marker for the super-resolution live imaging of the dynamic structure of the mitochondrial cristae. Proc. Natl Acad. Sci. USA.

[37] Yang, X. et al. Mitochondrial dynamics quantitatively revealed by STED nanoscopy with an enhanced squaraine variant probe. *Nat. Commun.* **11**, 3699 (2020). 10.1038/s41467-020-17546-1 PMC7382495 32709877

[38] Huang, X. et al. Fast, long-term, super-resolution imaging with Hessian structured illumination microscopy. *Nat. Biotechnol.* **36**, 451–459 (2018). 10.1038/nbt.4115 29644998

[39] Zhao, W. et al. Sparse deconvolution improves the resolution of live-cell super-resolution fluorescence microscopy. *Nat. Biotechnol.* **40**, 606–617 (2022). 10.1038/s41587-021-01092-2 34782739

[40] Qiao, C. et al. Rationalized deep learning super-resolution microscopy for sustained live imaging of rapid cellular processes. *Nat. Biotechnol.* **41**, 367–377 (2023). 10.1038/s41587-022-01471-3 36203012

[41] Schermelleh L (2019). Super-resolution microscopy demystified. Nat. Cell Biol..

[42] Fujiwara TK (2023). Development of ultrafast camera-based single fluorescent-molecule imaging for cell biology. J. Cell Biol..

[43] Dean KM (2015). Microfluidics-based selection of red-fluorescent proteins with decreased rates of photobleaching. Integr. Biol..

[44] Dean KM (2015). High-speed multiparameter photophysical analyses of fluorophore libraries. Anal. Chem..

[45] Ward, W. W. in *Green Fluorescent Protein* (eds. Chalfie, M. & Kain, S. R.) Ch. 3 (Wiley-Liss, 1998).

[46] Tsien RY (1998). The green fluorescent protein. Ann. Rev. Biochem..

[47] Nagai T (2002). A variant of yellow fluorescent protein with fast and efficient maturation for cell-biological applications. Nat. Biotechnol..

[48] Karasawa S, Araki T, Yamamoto-Hino M, Miyawaki A (2003). A green-emitting fluorescent protein from Galaxeidae coral and its monomeric version for use in fluorescence labeling. J. Biol. Chem..

[49] Szymczak AL (2004). Correction of multi-gene deficiency in vivo using a single ‘self-cleaving’ 2A peptide-based retroviral vector. Nat. Biotechnol..

[50] Winter G (2018). DIALS: implementation and evaluation of a new integration package. Acta Cryst..

[51] McCoy AJ (2007). *Phaser* crystallographic software (2007). J. Appl. Cryst..

[52] Afonine PV (2012). Towards automated crystallographic structure refinement with phenix.refine. Acta Cryst..

[53] Emsley P, Lohkamp B, Scott WG, Cowtan K (2010). Features and development of Coot. Acta Cryst..

[54] Shimozono S, Miyawaki A (2008). Engineering FRET constructs using CFP and YFP. Methods Cell Biol..

[55] Lin Y (2015). Quantifying and optimizing single-molecule switching nanoscopy at high speeds. PLoS ONE.

[56] Takagi, M. et al. Ki-67 and condensins support the integrity of mitotic chromosomes through distinct mechanisms. *J. Cell Sci.* **131**, jcs212092 (2018). 10.1242/jcs.212092 29487178

[57] Yesbolatova A, Natsume T, Hayashi K, Kanemaki MT (2019). Generation of conditional auxin-inducible degron (AID) cells and tight T control of degron-fused proteins using the degradation inhibitor auxinole. Methods.

[58] Otsu N (1979). A threshold selection method from gray-level histogram. IEEE Trans. Syst. Man Cybern..

