# Supplementary material for: StayGold variants for molecular fusion and membrane-targeting applications
Source: Nat Methods. 2023 Nov 30;21(4):648–56. doi: 10.1038/s41592-023-02085-6 (PMC11009113; doi:10.1038/s41592-023-02085-6)
Supplement: Supplementary file 1 — Supplementary Notes 1–8, Figs. 1–20, Tables 1–3, Video Captions 1–12 and References. [file 41592_2023_2085_MOESM1_ESM.pdf]

nature methods

Article https://doi.org/10.1038/s41592-023-02085-6

StayGold variants for molecular fusion and membrane-targeting applications

In the format provided by the authors and unedited

Supplementary information

StayGold variants for molecular fusion and membrane targeting applications

Table of Contents

Supplementary Notes 1–8

Supplementary Figures 1–20

Supplementary Tables 1–3

Supplementary Video Captions 1–12

Supplementary References

Supplementary Note 1
For example, mEGFP scored 89.8%, which was indeed higher than the score of its parent EGFP (83.4 %). A larger difference was observed between mVenus and Venus, which scored 92.8% and 55.9%, respectively. Among three mFPs that have been characterized to be very bright, mClover3 gave a high score (87.0%), whereas mNeonGreen and mGreenLantern showed relatively modest scores (74.2% and 83.1%, respectively). Remarkably, tdTomato showed a low score (57.7%), consistent with that reported by Cranfill et al. for this tdFP (57.6%) (ref. 1).

A substantial number of whorls tend to appear in very bright cells in OSER assays even with mFPs; the ER membrane should have a capacity for exogenous protein accumulation, and high-level expression of CytERM-FP exceeding the capacity might produce whorl structures efficiently. In fact, the results suggested a positive correlation between the degree of whorl appearance and the expression level of CytERM-Venus or CytERM-tdTomato. Although such gross overexpression artifacts have been eliminated deliberately, a complicated issue of how the threshold should be set has emerged[1].

Supplementary Note 2
Because the dimerization might be responsible for the outstanding photostability of StayGold, we initially suspected that creating a highly photostable and bright variant that is truly monomeric would be difficult. In fact, we found that complete disruption of the interactions at the interface resulted in the generation of only light-sensitive products that were hardly recovered by subsequent random mutagenesis.

Supplementary Note 3
In most oxygen-dependent maturation experiments, FP-expressing *E. coli* cells are initially grown under anaerobic conditions[2–4]. Then, after unsealing the test tubes or flasks, FP-containing lysates are prepared by sonication and centrifugation in the atmosphere for use in subsequent fluorescence measurements. This conventional biochemical procedure takes at least several minutes. We noticed that such a long time lag prevented us from characterizing the very rapid maturation kinetics of mStayGold. In the present study, we directly monitored the development of fluorescence from intact bacterial colonies immediately after they were exposed to air, and found that the early rapid phase of mStayGold maturation was completed within 5 min; this monomer matured much faster and with a greater yield than StayGold or mStayGold2 (Supplementary Fig. 9b).

Supplementary Note 4

In all cases, it is advised that a flexible and durable linker be used for fusion to minimize both the folding interference of the two domains and proteolysis. For this purpose, we basically used a 'Coupler linker', a triple repeat of the amino acid linker Gly-Gly-Gly-Gly-Ser [(GGGGS)3], which is denoted as '=' in this paper[5].

Supplementary Note 5

To advance toward a real understanding of the spatiotemporal regulation of a biological event through live cell imaging, in general, careful consideration of the expression levels of FP-tagged proteins is required. Whereas the high photostability of StayGold variants is expected to allow for visualization with low-level expression, we discuss this point as follows. First, we believe that the HCT116 cell line expressing endogenous condensin I tagged with td5oxStayGold should provide a nearly perfect situation. Second, we reason that the C-terminal region of Giantin (GianCreg) tagged with td5StayGold can be adequately overexpressed for the purpose of labeling the whole Golgi apparatus. Third, we verified that microtubules and actin filaments labeled by StayGold variants behaved in the same manner as those labeled with common FPs. Fourth, because mitochondrial complex IV containing subunit COX8a was found to exist in free form as well as to be complexed with complexes I and III (ref. 6), we reason that COX8a tagged with mStayGold can be overexpressed to label the inner mitochondrial membrane. In all the cases with overexpression, we selected dim or moderately bright cells for observation. We also confirmed that all the stable transformants used for sustained imaging in this study—specifically, HCT116 cells expressing CAP-H-td5oxStayGold (Fig. 4), HeLa cells expressing td5StayGold(c4)=GianCreg (Extended Data Fig. 7), and HeLa cells expressing COX8a=mStayGold (Supplementary Fig. 20)—proliferated normally. It is thus assumed that the StayGold variants entered the biological systems with minimal perturbation.

Supplementary Note 6

In this case, the illumination point spread function was calculated to have an Airy disk diameter of 0.626 μm. In this experiment, we used a zoom factor of 2× and a pixel number of 1,024 × 1,024; the pixel size was calculated to be 0.156 μm, which was a quarter of the Airy disk diameter for ideal sampling.

Supplementary Note 7

Although we have encountered a potential limitation of the StayGold technology, i.e., its

preferential applicability to WF microscopy, structured illumination microscopy (SIM), and multi-beam (spinning-disk) LSCM (ref. 7), we hope that the application of mStayGold and mStayGold2 to single-beam LSCM will overcome this limitation.

Supplementary Note 8

The normalized fluorescence of mNeonGreen peaked at approximately 30 h and gradually decreased thereafter. This tendency was reproducibly observed and indicative of the degradation of the β-barrel of this FP inside the cell. As a result, the normalized fluorescence intensity of mNeonGreen was nearly half that of mStayGold at 48 h, consistent with the results of cellular brightness (Fig. 3b).

Supplementary Fig. 1

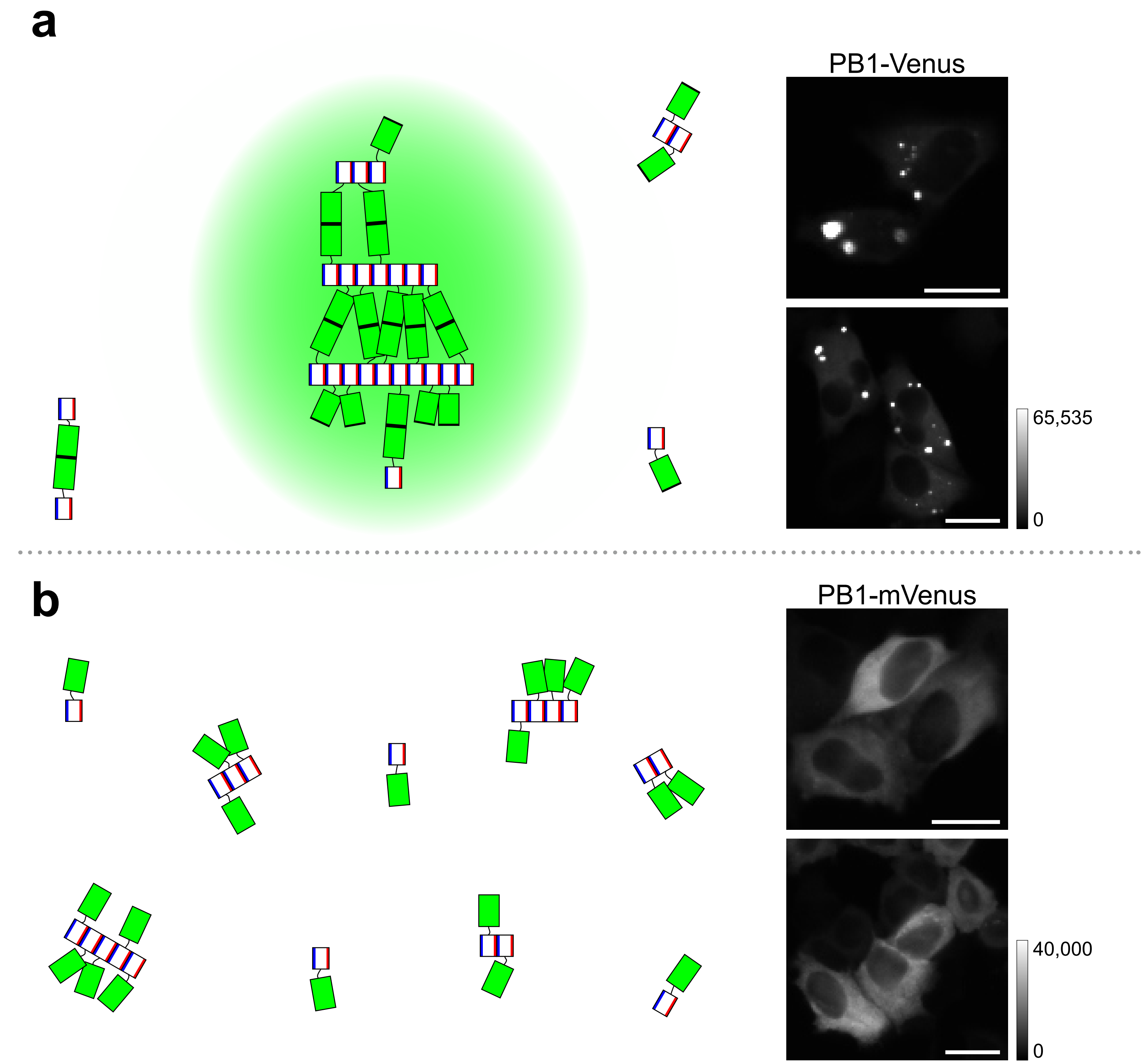

Supplementary Fig. 1 | Fluoppi assay using PB1 tag for assessment of FP monomericity.
The p62 PB1 domain is fused to a green-emitting FP (green), which is dimeric (**a**) or monomeric (**b**).
left, PB1 self-associates via an equilibrium in a front-to-back topology to form a high–molecular-weight homo-oligomer. The conserved acidic/hydrophobic and lysine/arginine residues of PB1 are indicated by red and blue bars, respectively.
a, Schematic showing that, as the FP homo-dimerizes, multiple PB1-FP molecules build crosslinks, resulting in the cytosolic formation of fluorescent puncta (green shading). The dimer interfaces of the FP are indicated by thick black bars.
b, Schematic showing that, due to the monomericity of the FP, PB1-FP and its oligomers diffuse freely throughout the cytosol.
right, WF images of cells with (**a**) and without (**b**) puncta; the images are representative of 3 repetitions (n = 3 independent transfections). Each gray scale indicates that the lowest and highest fluorescence intensities. Approximately 40% of PB1-Venus–expressing HeLa cells exhibited multiple punctate signals in the cytoplasmic compartment (**a**), whereas nearly 100% of the PB1-mVenus–expressing HeLa cells showed homogenous signal distribution (**b**) (see Extended Data Fig. 2). Scale bars, 20 µm.

Supplementary Fig. 2

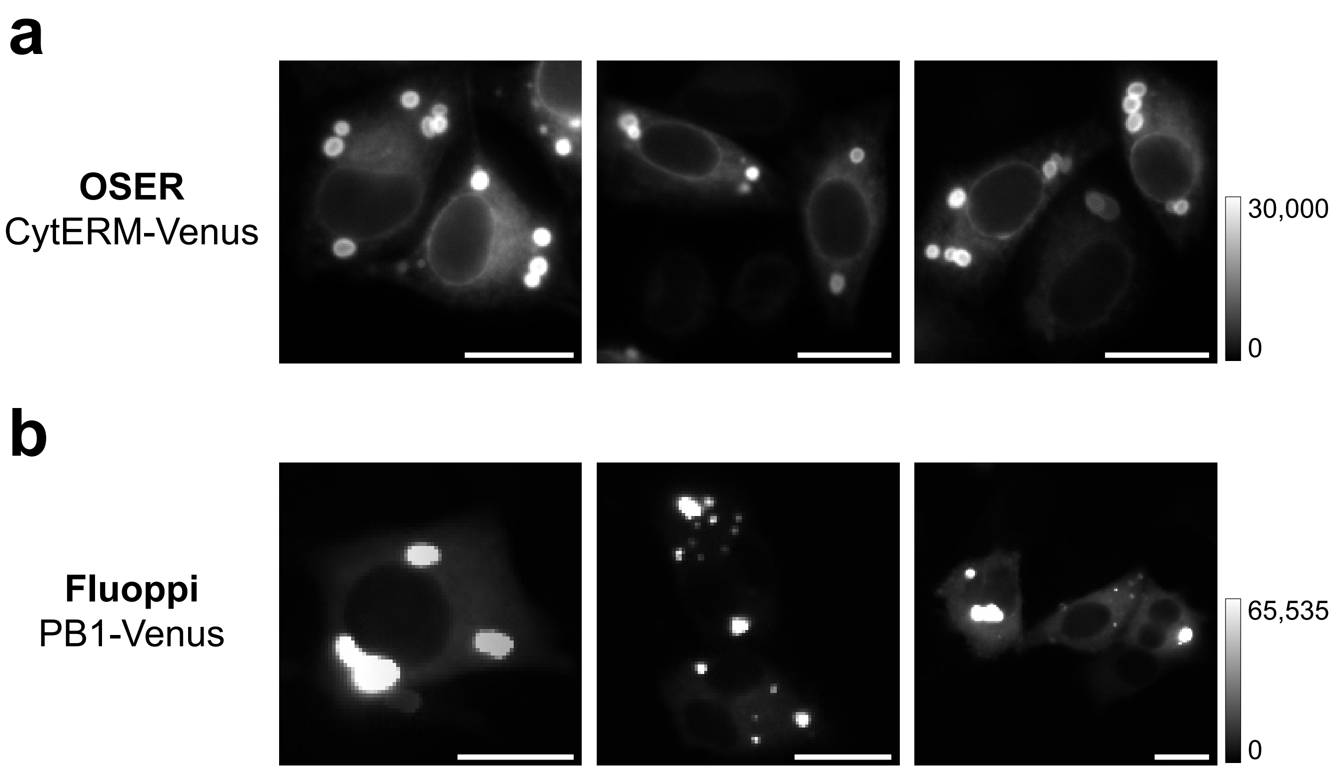

Supplementary Fig. 2 | Comparison between OSER and Fluoppi for the assessment of Venus monomericity/dispersibility.
a, OSER; three typical images of HeLa cells that showed whorls of CytERM-Venus signals.
b, Fluoppi; three typical images of HeLa cells that showed puncta of PB1-Venus signals. PB1-Venus appeared to yield more efficient signal concentration with less background than CytERM-Venus. Each gray scale indicates that the lowest and highest fluorescence intensities. Scale bars, 20 μm. Representative of *n* = 3 transfections for OSER and Fluoppi each.

Supplementary Fig. 3

```
SG              MAST------ ---PFKFQLK GTINGKSFTV EGEGEGNSHE GSHKGKYVCT SGKLPMSWAA LGTSFGYGMK YYTKYPSGLK NWFHEVMPEG FTYDRHIQYK GDGSIHAKHQ HFMKNGTYHN IVEFTGQDFK ENSPVLTGDM NVSLPNEVQH IPRDDGVECP VTLLYPLLSD KSKCVEAHQN TICKPLHNQP APDVPYHWIR KQYTQSKDDT EERDHICQSE TLEAHL
(n1)SG          MVSTGEELFT GVVPFKFQLK GTINGKSFTV EGEGEGNSHE GSHKGKYVCT SGKLPMSWAA LGTSFGYGMK YYTKYPSGLK NWFHEVMPEG FTYDRHIQYK GDGSIHAKHQ HFMKNGTYHN IVEFTGQDFK ENSPVLTGDM NVSLPNEVQH IPRDDGVECP VTLLYPLLSD KSKCVEAHQN TICKPLHNQP APDVPYHWIR KQYTQSKDDT EERDHICQSE TLEAHL
(n1)oxSG        MVSTGEELFT GVVPFKFQLK GTINGKSFTV EGEGEGNSHE GSHKGKYVCT SGKLPMSWAA LGTSFGYGMK YYTKYPSGLK NWFHEVMPEG FTYDRHIQYK GDGSIHAKHQ HFMKNGTYHN IVEFTGQDFK ENSPVLTGDM NVSLPNEVQH IPRDDGVECP VTLLYPLLSD KSKCVEAYQN TIIKPLHNQP APDVPYHWIR KQYTQSKDDT EERDHIIQSE TLEAHL
(n1)ox2SG       MVSTGEELFT GVVPFKFQLK GTINGKSFTV EGEGEGNSHE GSHKGKYVCT SGKLPMSWAA LGTSFGYGMK YYTKYPSGLK NWFHEVMPEG FTYDRHIQYK GDGSIHAKHQ HFMKNGTYHN IVEFTGQDFK ENSPVLTGDM NVSLPNEVQH IPRDDGVECP VTLLYPLLSD KSKCVEAFQN TIIKPLHNQP APDVPYHWIR KQYTQSKDDT EERDHIIQSE TLEAHL
```

monomer

```
QC2-6             MVSTGEELFT GVVPFKFQLK GTINGKSFTV EGEGEGNSHE GSHKGKYVCT SGKLPMSWAA LGTSFGYGMK YYTKYPSGLK NWFHEVMPEG FTYDRHIQYK GDGSIHAKHQ HFMKNGTYHN IVEFTGQDFK ENSPVLTGDM DVSLPNEVQH IPRDDGVECT VTLTYPLLSD ESKCVEAYQN TIIKPLHNQP APDVPYHWIR KQYTQSKDDT EERDHIIQSE TLEAHL
QC2-6 FIQ (mSG)     MVSTGEELFT GVVPFKFQLK GTINGKSFTV EGEGEGNSHE GSHKGKYVCT SGKLPMSWAA LGTSFGYGMK YYTKYPSGLK NWFHEVMPEG FTYDRHIQYK GDGSIHAKHQ HFMKNGTYHN IVEFTGQDFK ENSPVLTGDM DVSLPNEVQH IPIDDGVECT VTLQYPLLSD ESKCVEAYQN TIIKPLHNQP APDVPFHWIR KQYTQSKDDT EERDHIIQSE TLEAHL
QC2-6(PT) (mSG2)  MVSTGEELFT GVVPFKFQLK GTINGKSFTV EGEGEGNSHE GSHKGKYVCT SGKLPMSWAA LGTSFGYGMK YYTKYPSGLK NWFHEVMPEG FTYDRHIQYK GDGSIHAKHQ HFMKNGTYHN IVEFTGQDFK ENSPVLTGDM DVSLPNEVQH IPRDDGVECT VTLTYPLLSD ESKCVEAYQN TIIKPLHNQP APDVPYHWIR KQYTQSKDDT EERDHIIQSE TLEAHLYSRT KLE
```

tandem dimer

```
tdSG        MVSTGEELFT GVVPFKFQLK GTINGKSFTV EGEGEGNSHE GSHKGKYVCT SGKLPMSWAA LGTSFGYGMK YYTKYPSGLK NWFHEVMPEG FTYDRHIQYK GDGSIHAKHQ HFMKNGTYHN IVEFTGQDFK ENSPVLTGDM NVSLPNEVQH IPRDDGVECP VTLLYPLLSD KSKCVEAHQN TICKPLHNQP APDVPYHWIR KQYTQSKDDT EERDHICQSE TLEAHLPWHE PSASAV
tdoxSG      MVSTGEELFT GVVPFKFQLK GTINGKSFTV EGEGEGNSHE GSHKGKYVCT SGKLPMSWAA LGTSFGYGMK YYTKYPSGLK NWFHEVMPEG FTYDRHIQYK GDGSIHAKHQ HFMKNGTYHN IVEFTGQDFK ENSPVLTGDM NVSLPNEVQH IPRDDGVECP VTLLYPLLSD KSKCVEAYQN TIIKPLHNQP APDVPYHWIR KQYTQSKDDT EERDHIIQSE TLEAHLPWHE PSASAV
td2oxSG     MVSTGEELFT GVVPFKFQLK GTINGKSFTV EGEGEGNSHE GSHKGKYVCT SGKLPMSWAA LGTSFGYGMK YYTKYPSGLK NWFHEVMPEG FTYDRHIQYK GDGSIHAKHQ HFMKNGTYHN IVEFTGQDFK ENSPVLTGDM NVSLPNEVQH IPRDDGVECP VTLLYPLLSD KSKCVEAYQN TIIKPLHNQP APDVPYHWIR KQYTQSKDDT EERDHIIQSE TLEAHLPWHE PSASAV
td3oxSG     MVSTGEELFT GVVPFKFQLK GTINGKSFTV EGEGEGNSHE GSHKGKYVCT SGKLPMSWAA LGTSFGYGMK YYTKYPSGLK NWFHEVMPEG FTYDRHIQYK GDGSIHAKHQ HFMKNGTYHN IVEFTGQDFK ENSPVLTGDM NVSLPNEVQH IPRDDGVECP VTLLYPLLSD KSKCVEAYQN TIIKPLHNQP APDVPYHWIR KQYTQSKDDT EERDHIIQSE TLEAHLPWHE PSASAV
td4oxSG     MVSTGEELFT GVVPFKFQLK GTINGKSFTV EGEGEGNSHE GSHKGKYVCT SGKLPMSWAA LGTSFGYGMK YYTKYPSGLK NWFHEVMPEG FTYDRHIQYK GDGSIHAKHQ HFMKNGTYHN IVEFTGQDFK ENSPVLTGDM NVSLPNEVQH IPRDDGVECP VTLLYPLLSD KSKCVEAYQN TIIKPLHNQP APDVPYHWIR KQYTQSKDDT EERDHIIQSE TLEAHLPWHE PSASAV

td5SG       MVSTGEELFT GVVPFKFQLK GTINGKSFTV EGEGEGNSHE GSHKGKYVCT SGKLPMSWAA LGTSFGYGMK YYTKYPSGLK NWFHEVMPEG FTYDRHIQYK GDGSIHAKHQ HFMKNGTYHN IVEFTGQDFK ENSPVLTGDM NVSLPNEVQH IPRDDGVECP VTLLYPLLSD KSKCVEAHQN TICKPLHNQP APDVPYHWIR KQYTQSKDDT EERDHICQSE TLEAHLPWHE PSASAV
td5oxSG     MVSTGEELFT GVVPFKFQLK GTINGKSFTV EGEGEGNSHE GSHKGKYVCT SGKLPMSWAA LGTSFGYGMK YYTKYPSGLK NWFHEVMPEG FTYDRHIQYK GDGSIHAKHQ HFMKNGTYHN IVEFTGQDFK ENSPVLTGDM NVSLPNEVQH IPRDDGVECP VTLLYPLLSD KSKCVEAYQN TIIKPLHNQP APDVPYHWIR KQYTQSKDDT EERDHIIQSE TLEAHLPWHE PSASAV
td6oxSG     MVSTGEELFT GVVPFKFQLK GTINGKSFTV EGEGEGNSHE GSHKGKYVCT SGKLPMSWAA LGTSFGYGMK YYTKYPSGLK NWFHEVMPEG FTYDRHIQYK GDGSIHAKHQ HFMKNGTYHN IVEFTGQDFK ENSPVLTGDM NVSLPNEVQH IPRDDGVECP VTLLYPLLSD KSKCVEAYQN TIIKPLHNQP APDVPYHWIR KQYTQSKDDT EERDHIIQSE TLEAHLPWHE PSASAV
td7oxSG     MVSTGEELFT GVVPFKFQLK GTINGKSFTV EGEGEGNSHE GSHKGKYVCT SGKLPMSWAA LGTSFGYGMK YYTKYPSGLK NWFHEVMPEG FTYDRHIQYK GDGSIHAKHQ HFMKNGTYHN IVEFTGQDFK ENSPVLTGDM NVSLPNEVQH IPRDDGVECP VTLLYPLLSD KSKCVEAYQN TIIKPLHNQP APDVPYHWIR KQYTQSKDDT EERDHIIQSE TLEAHLPWHE PSASAV

td8oxSG     MVSTGEELFT GVVPFKFQLK GTINGKSFTV EGEGEGNSHE GSHKGKYVCT SGKLPMSWAA LGTSFGYGMK YYTKYPSGLK NWFHEVMPEG FTYDRHIQYK GDGSIHAKHQ HFMKNGTYHN IVEFTGQDFK ENSPVLTGDM NVSLPNEVQH IPRDDGVECP VTLLYPLLSD KSKCVEAYQN TIIKPLHNQP APDVPYHWIR KQYTQSKDDT EERDHIIQSE TLEAHLYSRT KLE
td8ox2SG    MVSTGEELFT GVVPFKFQLK GTINGKSFTV EGEGEGNSHE GSHKGKYVCT SGKLPMSWAA LGTSFGYGMK YYTKYPSGLK NWFHEVMPEG FTYDRHIQYK GDGSIHAKHQ HFMKNGTYHN IVEFTGQDFK ENSPVLTGDM NVSLPNEVQH IPRDDGVECP VTLLYPLLSD KSKCVEAFQN TIIKPLHNQP APDVPYHWIR KQYTQSKDDT EERDHIIQSE TLEAHLYSRT KLE


tdSG        EFSAGGSAGG SAGGSAGGSA GGSGSAGGSA GGSTSAGGSA GGSAGGSAGG SAGGSGSAGG SAGGSTSAGG SAGGSAGGSA GGSAGGSGSA GGSAGGSTSA GGSAGGSAGG SAGGSAGGKL
tdoxSG      EFSAGGSAGG SAGGSAGGSA GGSGSAGGSA GGSTSAGGSA GGSAGGSAGG SAGGSGSAGG SAGGSTSAGG SAGGSAGGSA GGSAGGSGSA GGSAGGSTSA GGSAGGSAGG SAGGSAGGKL
tdox2SG     EFSAGGSAGG SAGGSAGGSA GGSGSAGGSA GGSTSAKL
tdox3SG     EFSAGGSAGG SAGGSAGGSA GGSGSAGGSA GGSTSAGGSA GGSAGGSAGG SAGGSGSAGG SAGGSTSAKL
tdox4SG     EFSAGGSAGG SAGGSAGGSA GGSGSAGGSA GGSTSAGGSA GGSAGGSAGG SAGGSGSAGG SAGGSTSAGG SAGGSAGGSA GGSAGGSGSA GGSAGGSTSA G

td5SG       GHGTGSTGSG SSGTASSEDN NID
td5oxSG     GHGTGSTGSG SSGTASSEDN NID
td6oxSG     RMGSTSGSTK GQLID
td7oxSG     RMGSTSGSGK PGSGEGSTKG QLID

td8oxSG     GHGTGSTGSG SSGTASSEDN NID
td8ox2SG    GHGTGSTGSG SSGTASSEDN NID


tdSG        MVSTGEELFT GVVPFKFQLK GTINGKSFTV EGEGEGNSHE GSHKGKYVCT SGKLPMSWAA LGTSFGYGMK YYTKYPSGLK NWFHEVMPEG FTYDRHIQYK GDGSIHAKHQ HFMKNGTYHN IVEFTGQDFK ENSPVLTGDM NVSLPNEVQH IPRDDGVECP VTLLYPLLSD KSKCVEAHQN TICKPLHNQP APDVPYHWIR KQYTQSKDDT EERDHICQSE TLEAHL
tdoxSG      MVSTGEELFT GVVPFKFQLK GTINGKSFTV EGEGEGNSHE GSHKGKYVCT SGKLPMSWAA LGTSFGYGMK YYTKYPSGLK NWFHEVMPEG FTYDRHIQYK GDGSIHAKHQ HFMKNGTYHN IVEFTGQDFK ENSPVLTGDM NVSLPNEVQH IPRDDGVECP VTLLYPLLSD KSKCVEAYQN TIIKPLHNQP APDVPYHWIR KQYTQSKDDT EERDHIIQSE TLEAHL
tdox2SG     MVSTGEELFT GVVPFKFQLK GTINGKSFTV EGEGEGNSHE GSHKGKYVCT SGKLPMSWAA LGTSFGYGMK YYTKYPSGLK NWFHEVMPEG FTYDRHIQYK GDGSIHAKHQ HFMKNGTYHN IVEFTGQDFK ENSPVLTGDM NVSLPNEVQH IPRDDGVECP VTLLYPLLSD KSKCVEAYQN TIIKPLHNQP APDVPYHWIR KQYTQSKDDT EERDHIIQSE TLEAHL
tdox3SG     MVSTGEELFT GVVPFKFQLK GTINGKSFTV EGEGEGNSHE GSHKGKYVCT SGKLPMSWAA LGTSFGYGMK YYTKYPSGLK NWFHEVMPEG FTYDRHIQYK GDGSIHAKHQ HFMKNGTYHN IVEFTGQDFK ENSPVLTGDM NVSLPNEVQH IPRDDGVECP VTLLYPLLSD KSKCVEAYQN TIIKPLHNQP APDVPYHWIR KQYTQSKDDT EERDHIIQSE TLEAHL
tdox4SG     MVSTGEELFT GVVPFKFQLK GTINGKSFTV EGEGEGNSHE GSHKGKYVCT SGKLPMSWAA LGTsFGYGMK YYTKYPSGLK NWFHEVMPEG FTYDRHIQYK GDGSIHAKHQ HFMKNGTYHN IVEFTGQDFK ENSPVLTGDM NVSLPNEVQH IPRDDGVECP VTLLYPLLSD KSKCVEAYQN TIIKPLHNQP APDVPYHWIR KQYTQSKDDT EERDHIIQSE TLEAHL

td5SG       MVSTGEELFT GVVPFKFQLK GTINGKSFTV EGEGEGNSHE GSHKGKYVCT SGKLPMSWAA LGTSFGYGMK YYTKYPSGLK NWFHEVMPEG FTYDRHIQYK GDGSIHAKHQ HFMKNGTYHN IVEFTGQDFK ENSPVLTGDM NVSLPNEVQH IPRDDGVECP VTLLYPLLSD KSKCVEAHQN TICKPLHNQP APDVPYHWIR KQYTQSKDDT EERDHICQSE TLEAHL
td5oxSG     MVSTGEELFT GVVPFKFQLK GTINGKSFTV EGEGEGNSHE GSHKGKYVCT SGKLPMSWAA LGTSFGYGMK YYTKYPSGLK NWFHEVMPEG FTYDRHIQYK GDGSIHAKHQ HFMKNGTYHN IVEFTGQDFK ENSPVLTGDM NVSLPNEVQH IPRDDGVECP VTLLYPLLSD KSKCVEAYQN TIIKPLHNQP APDVPYHWIR KQYTQSKDDT EERDHIIQSE TLEAHL
td6oxSG     MVSTGEELFT GVVPFKFQLK GTINGKSFTV EGEGEGNSHE GSHKGKYVCT SGKLPMSWAA LGTSFGYGMK YYTKYPSGLK NWFHEVMPEG FTYDRHIQYK GDGSIHAKHQ HFMKNGTYHN IVEFTGQDFK ENSPVLTGDM NVSLPNEVQH IPRDDGVECP VTLLYPLLSD KSKCVEAYQN TIIKPLHNQP APDVPYHWIR KQYTQSKDDT EERDHIIQSE TLEAHL
td7oxSG     MVSTGEELFT GVVPFKFQLK GTINGKSFTV EGEGEGNSHE GSHKGKYVCT SGKLPMSWAA LGTSFGYGMK YYTKYPSGLK NWFHEVMPEG FTYDRHIQYK GDGSIHAKHQ HFMKNGTYHN IVEFTGQDFK ENSPVLTGDM NVSLPNEVQH IPRDDGVECP VTLLYPLLSD KSKCVEAYQN TIIKPLHNQP APDVPYHWIR KQYTQSKDDT EERDHIIQSE TLEAHL

td8oxSG     MVSTGEELFT GVVPFKFQLK GTINGKSFTV EGEGEGNSHE GSHKGKYVCT SGKLPMSWAA LGTSFGYGMK YYTKYPSGLK NWFHEVMPEG FTYDRHIQYK GDGSIHAKHQ HFMKNGTYHN IVEFTGQDFK ENSPVLTGDM NVSLPNEVQH IPRDDGVECP VTLLYPLLSD KSKCVEAYQN TIIKPLHNQP APDVPYHWIR KQYTQSKDDT EERDHIIQSE TLEAHL
td8ox2SG    MVSTGEELFT GVVPFKFQLK GTINGKSFTV EGEGEGNSHE GSHKGKYVCT SGKLPMSWAA LGTSFGYGMK YYTKYPSGLK NWFHEVMPEG FTYDRHIQYK GDGSIHAKHQ HFMKNGTYHN IVEFTGQDFK ENSPVLTGDM NVSLPNEVQH IPRDDGVECP VTLLYPLLSD KSKCVEAFQN TIIKPLHNQP APDVPYHWIR KQYTQSKDDT EERDHIIQSE TLEAHL
```

Supplementary Fig. 3 | Amino acid sequence alignment of StayGold and its variants.
Adaptors and linkers are shaded in the same colors as in Extended Data Fig. 3. StayGold is abbreviated as SG. Either (n1)SG or (n1)oxSG was used as the starting material. Their N-terminus had a mutation A2V relative to that of SG.

Supplementary Fig. 4

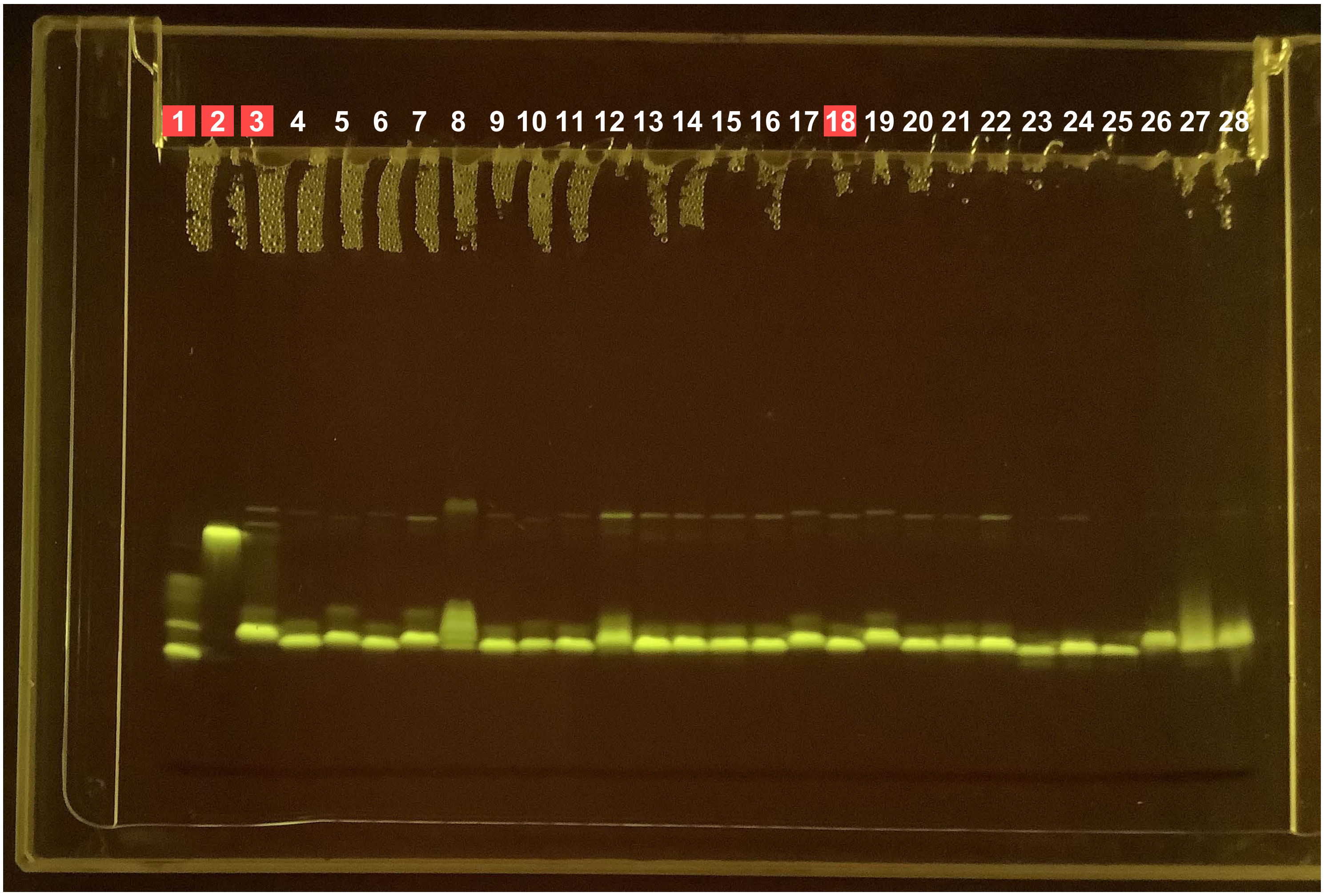

1) EGFP
2) StayGold
3) QC2-6
4) QC2-6 Y187F / R144I ran1
5) QC2-6 Y187F / R144I ran2
6) QC2-6 Y187F / R144I ran3
7) QC2-6 Y187F / R144I ran4
8) QC2-6 Y187F / R144I ran5
9) QC2-6 Y187F / R144I ran6
10) QC2-6 Y187F / R144I ran7
11) QC2-6 Y187F / R144I ran8
12) QC2-6 Y187F / R144I ran9
13) QC2-6 Y187F / R144I
14) QC2-6 Y187F / R144I / T155F
15) QC2-6 Y187F / R144I / T155H
16) QC2-6 Y187F / R144I / T155I
17) QC2-6 Y187F / R144I / T155K
18) QC2-6 Y187F / R144I / T155Q
19) QC2-6 Y187F / R144I / T155R
20) QC2-6 Y187F / R144I / T155V
21) QC2-6 Y187F / R144I / T155W
22) QC2-6 Y187F / R144I / T155Y
23) QC2-6 R144E
24) QC2-6 R144E / T155I
25) QC2-6 R144E / T155M
26) QC2-6 R144E / T155R
27) QC2-6 R144E / T155V
28) QC2-6 R144E / T155Y

Supplementary Fig. 4 | Pseudonative SDS-PAGE of purified bacterially expressed proteins. A panel (an uncropped photo of the gel) showing the final stage of the revolution reaching QC2-6 FIQ (lane 18). QC2-6 (lane 3) and its derivatives were analyzed in reference to EGFP (lane 1) and StayGold (lane 2). QC2-6 FIQ (= QC2-6 Y187F/R144I/T155Q) exhibited a slightly greater mobility than QC2-6. Lanes 4–12: nine bright colonies were selected from a mutant library on the basis of products of error-prone PCR on QC2-6 Y187F/R144I. ran: random mutagenesis. Shown is a representative of $n = 4$ independent experiments that detected different electrophoretic mobility between QC2-6 and QC2-6 FIQ.

Supplementary Fig. 5

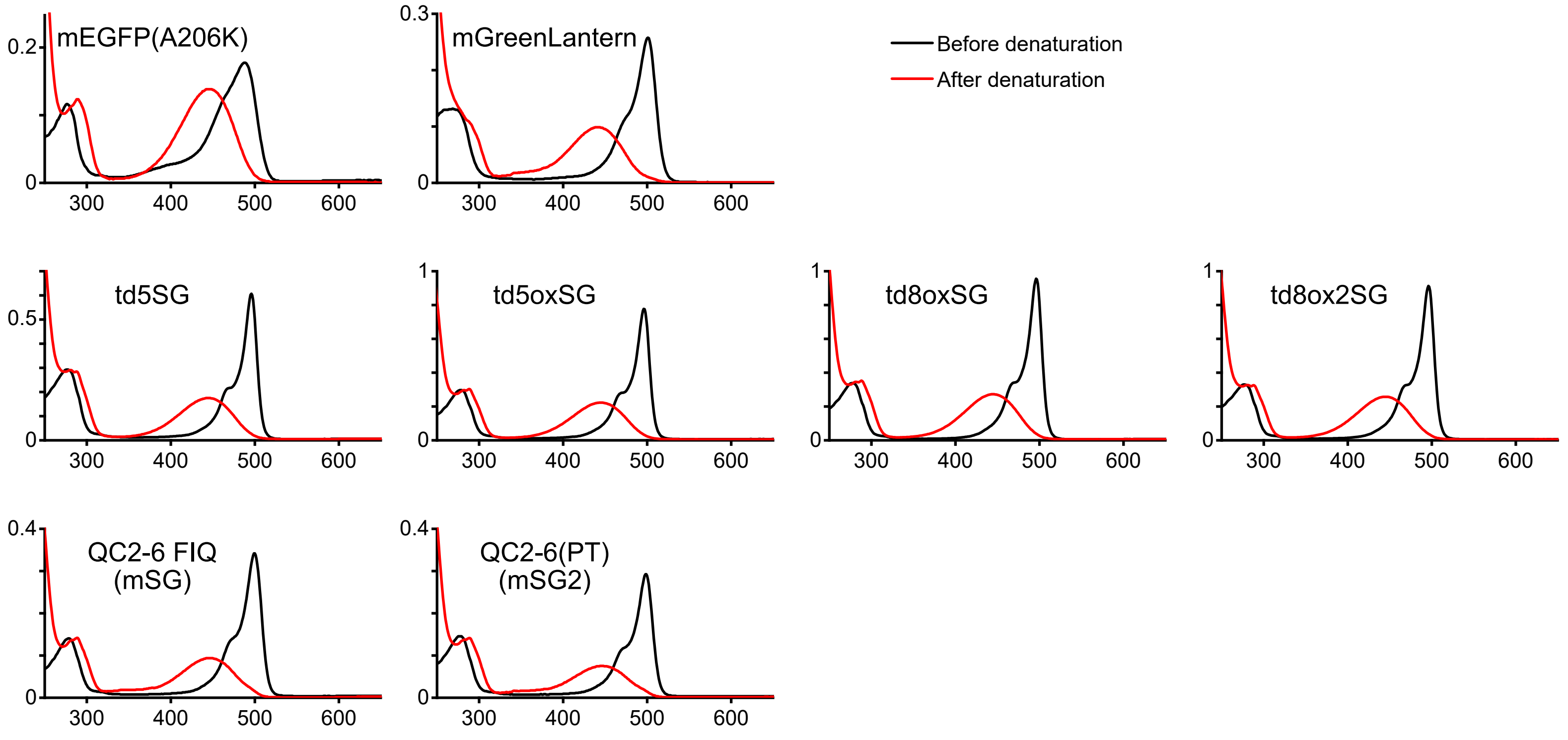

Supplementary Fig. 5 | Spectral assessment of inherent qualities of FP chromophores. Absorption spectra of FPs before (black line) and after (red line) denaturation with 0.1 M NaOH. The FP concentration was 10 μM, and the path length was 1 cm. All these FPs carry X-Tyr-Gly, a chromophore-forming tripeptide, and their alkali-denatured chromophores contain a dehydrotyrosine residue conjugated to the imidazolone group and absorb light maximally at 447 nm with a molar extinction coefficient of 44,000 M^{-1} cm^{-1}. This value was used for the determination of their absolute molar extinction coefficients (Table 1). StayGold is abbreviated as SG.

Supplementary Fig. 6

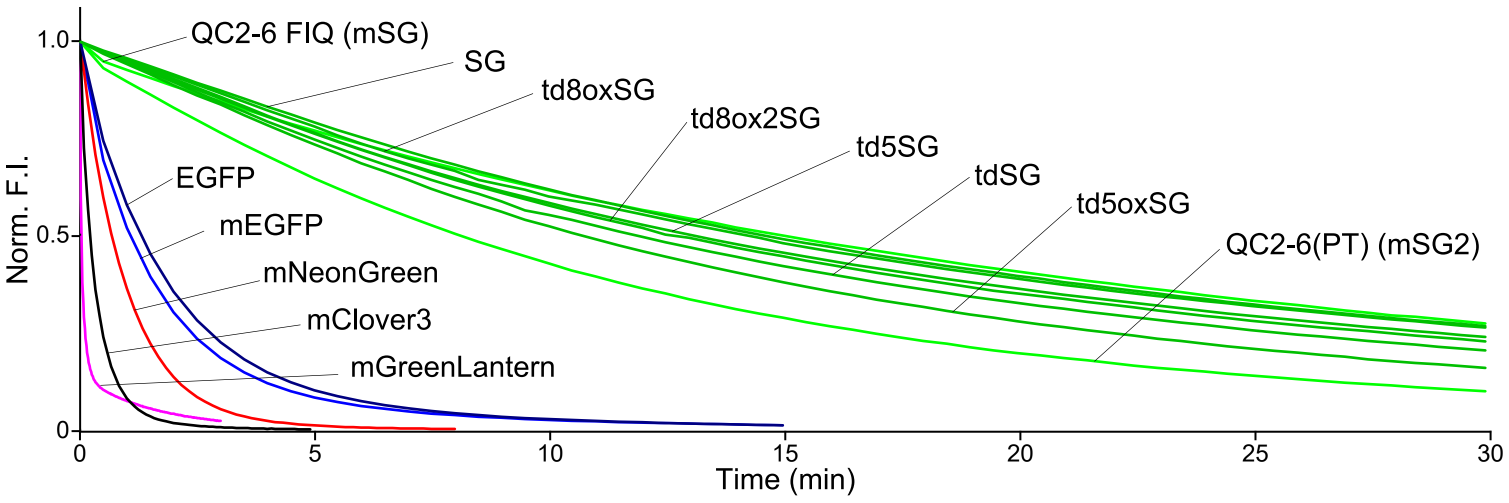

Supplementary Fig. 6 | Photostability of StayGold variants and reference green-emitting FPs in live cells.
Intensity-normalized curves. FPs were expressed as fusions to histone 2B (H2B) in HeLa cells. Illumination intensity, 8.66 W cm^{-2}. The curves shown are representative of three repetitions ($n = 3$ independent experiments). StayGold is abbreviated as SG. Curves are colored as follows. QC2-6 FIQ (mSG) and QC2-6(PT) (mSG2), green; SG and its tandem dimers, dark green; EGFP, dark blue; mEGFP, blue; mNeonGreen, red; mClover3, black; mGreenLantern, magenta. See Fig. 2.

Supplementary Fig. 7

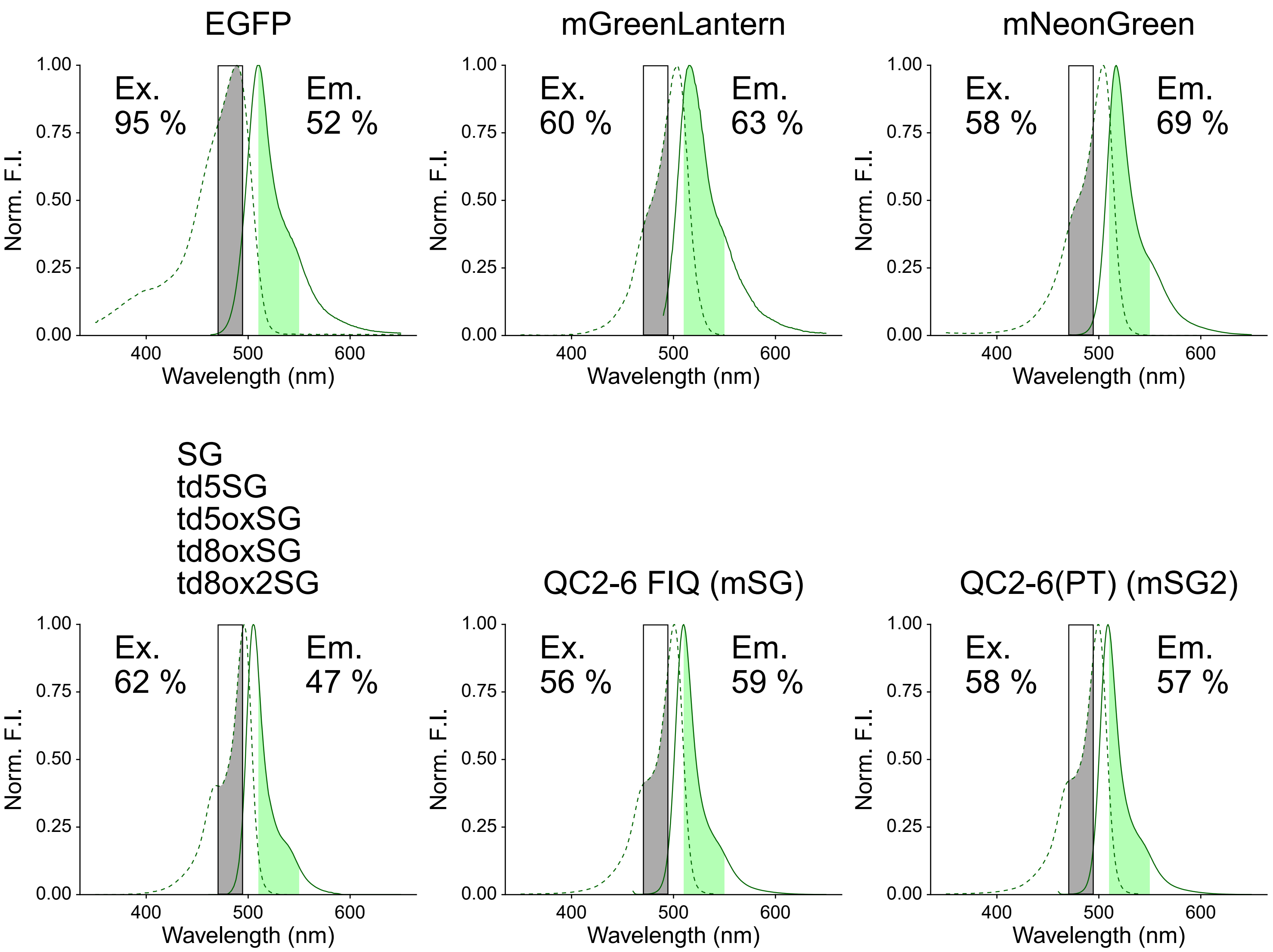

Supplementary Fig. 7 | Spectral throughputs of green-emitting FPs in the imaging system for determination of cellular brightness.

Normalized excitation (dotted line) and emission (solid line) spectra of individual FPs and transmissions occurring in the excitation (gray) and emission (light green) passbands. Relative excitation (Ex.) and emission detection (Em.) efficiencies are shown on the left and right sides of the spectra, respectively. Their products were used to calculate the correction factors (Supplementary Table 2).

StayGold is abbreviated as SG. See Fig. 3b.

Supplementary Fig. 8

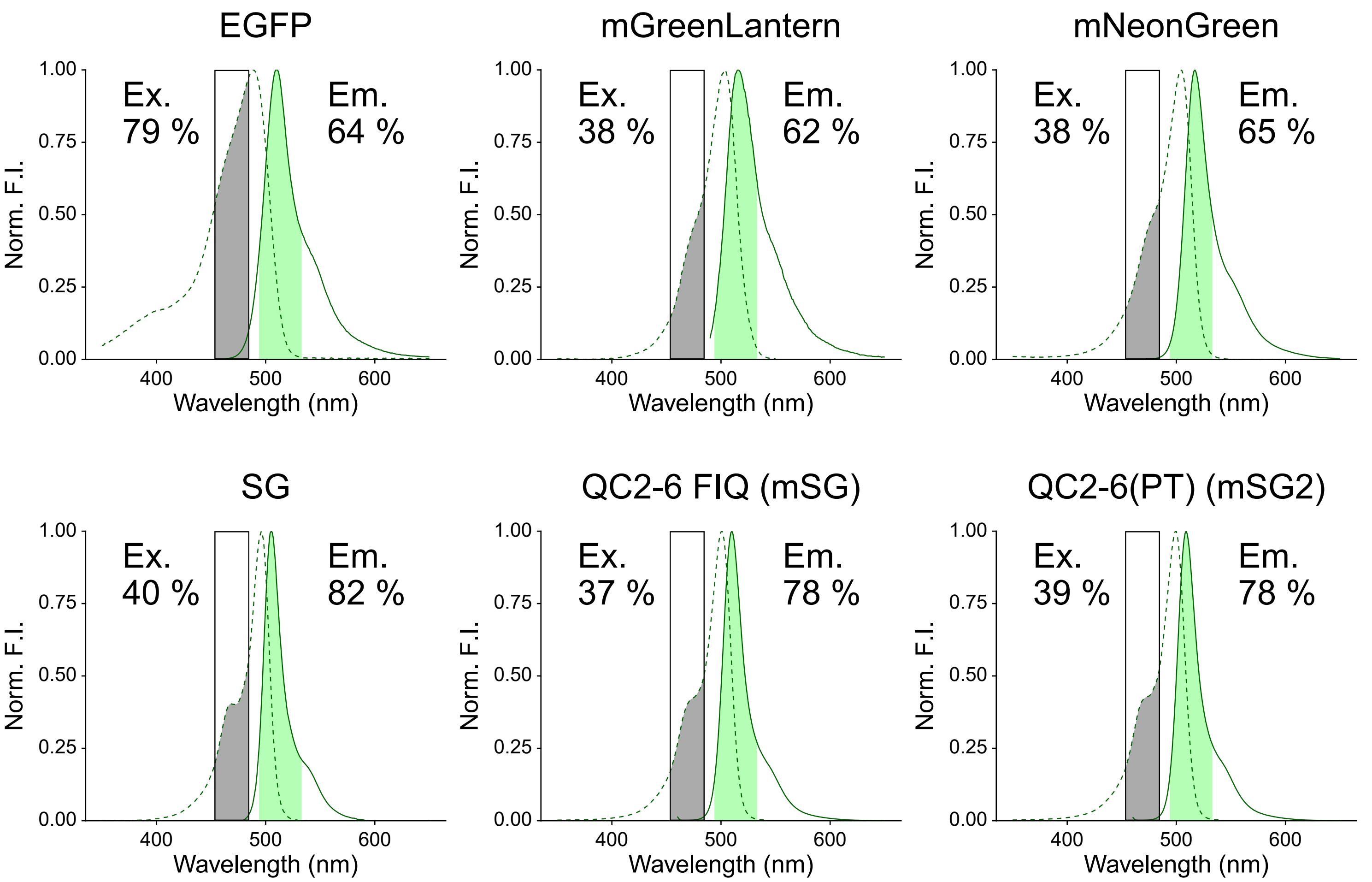

Supplementary Fig. 8 | Spectral throughputs of green-emitting FPs in the imaging system for observation of chromophore maturation.

Normalized excitation (dotted line) and emission (solid line) spectra of individual FPs and transmissions occurring in the excitation (gray) and emission (light green) passbands. Relative excitation (Ex.) and emission detection (Em.) efficiencies are shown on the left and right sides of the spectra, respectively. Their products were used to calculate the correction factors. StayGold is abbreviated as SG. See Fig. 3c.

Supplementary Fig. 9

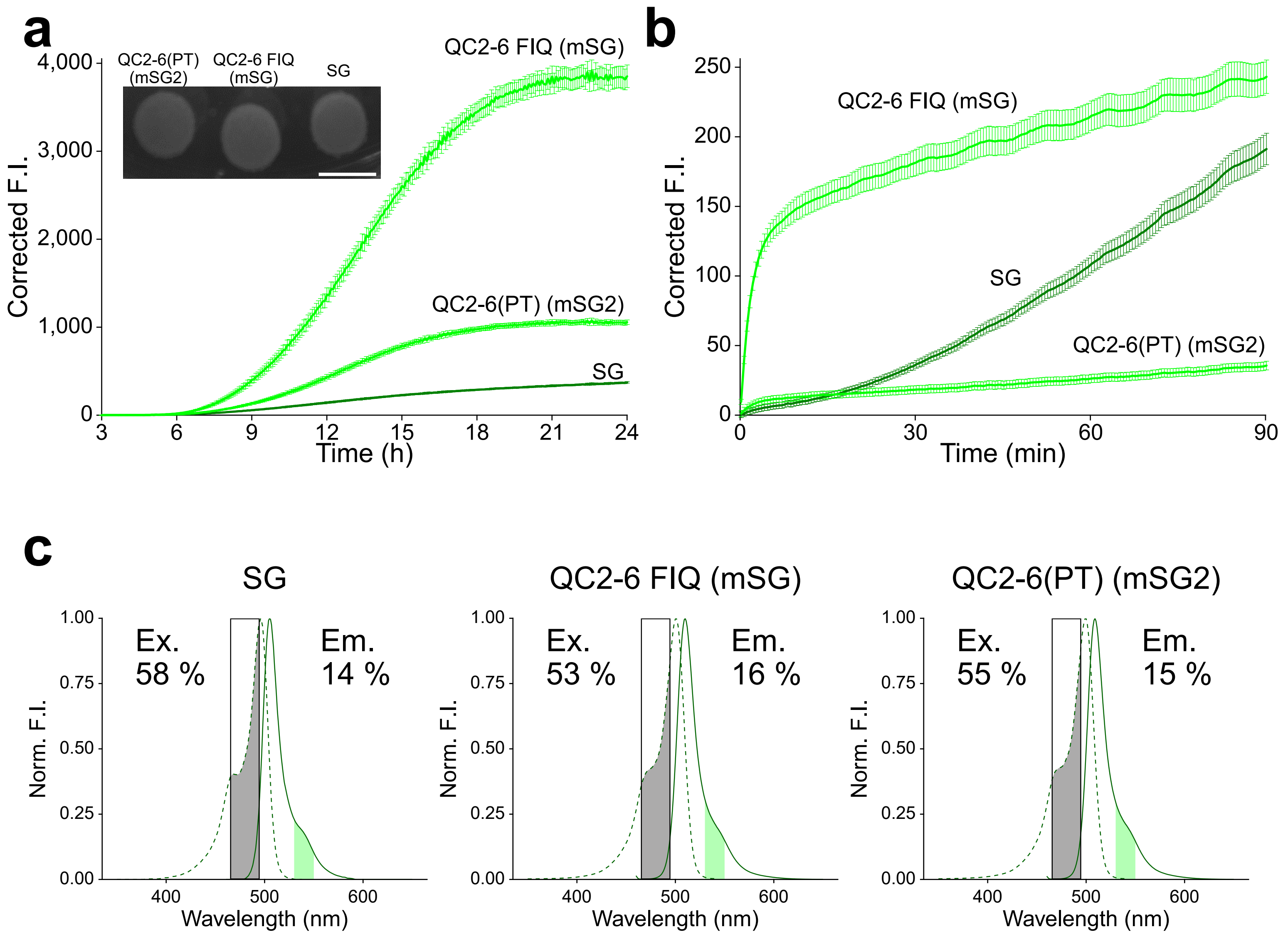

Supplementary Fig. 9 | Chromophore maturation in bacteria.
E. coli colonies expressing StayGold, mStayGold, and mStayGold2 on agar plates were directly imaged.
a, Fluorescence development in bacterial cells after plating at 37 °C. Data points are shown as means ± s.e.m. (n = 12 different colonies from 3 independent experiments). A photomicrograph showing the three colonies exhibiting the same growth rate. Scale bar, 5 mm.
b, Fluorescence development of anaerobically grown bacterial cells after exposure to air at 37 °C. Data points are shown as means ± s.e.m. (n = 18 different colonies from 3 independent experiments). See Supplementary Video 1.
a, b, Fluorescence intensities were corrected for the respective spectral throughputs (see below).
c, Spectral throughputs of StayGold, mStayGold, and mStayGold2 in the imaging system for observation of bacterial colonies. Normalized excitation (dotted line) and emission (solid line) spectra of individual FPs and transmissions occurring in the excitation (gray) and emission (light green) passbands. Relative excitation (Ex.) and emission detection (Em.) efficiencies are shown on the left and right sides of the spectra, respectively. Their products were used to calculate the correction factors. StayGold is abbreviated as SG.

Supplementary Fig. 10

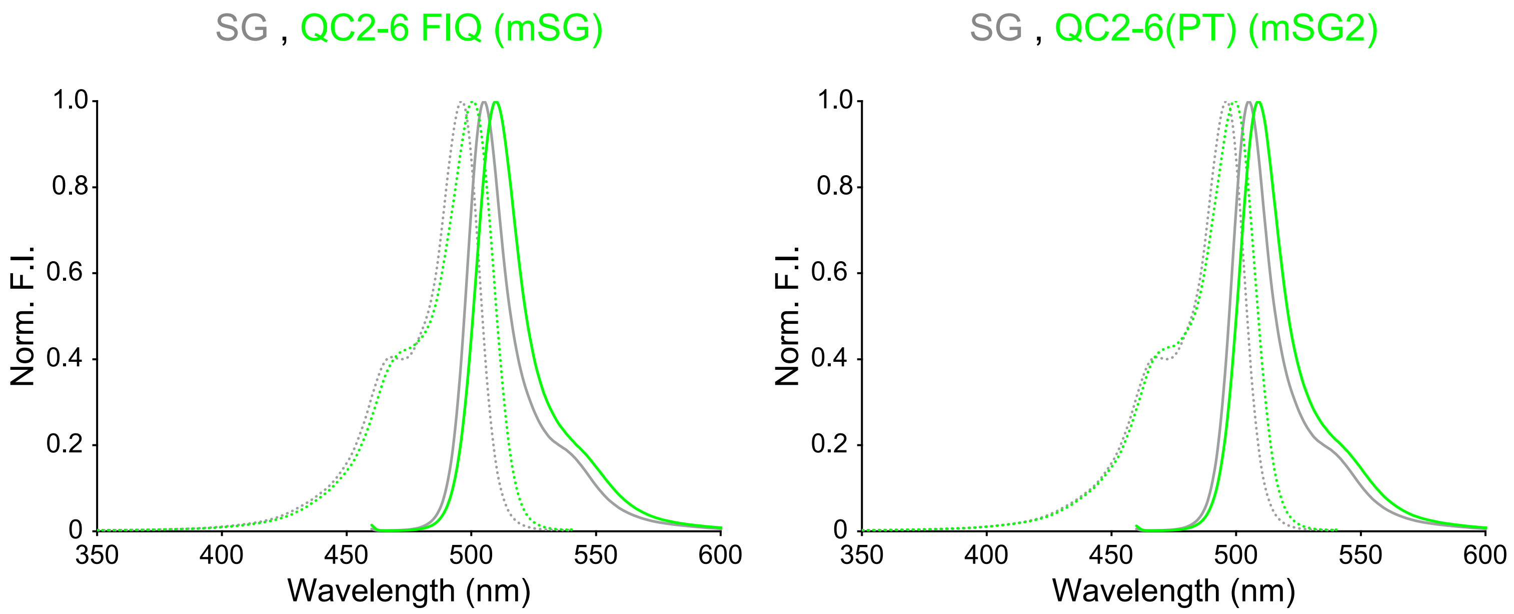

Supplementary Fig. 10 | Normalized excitation (green dotted line) and emission (green solid line) spectra of mStayGold and mStayGold2.
Superimposed to the spectra of StayGold (gray). The small Stokes shift of mSG and mSG2 should make it relatively difficult to maximize both excitation and emission collection but could be beneficial to homo-FRET application (ref. 8).

Supplementary Fig. 11

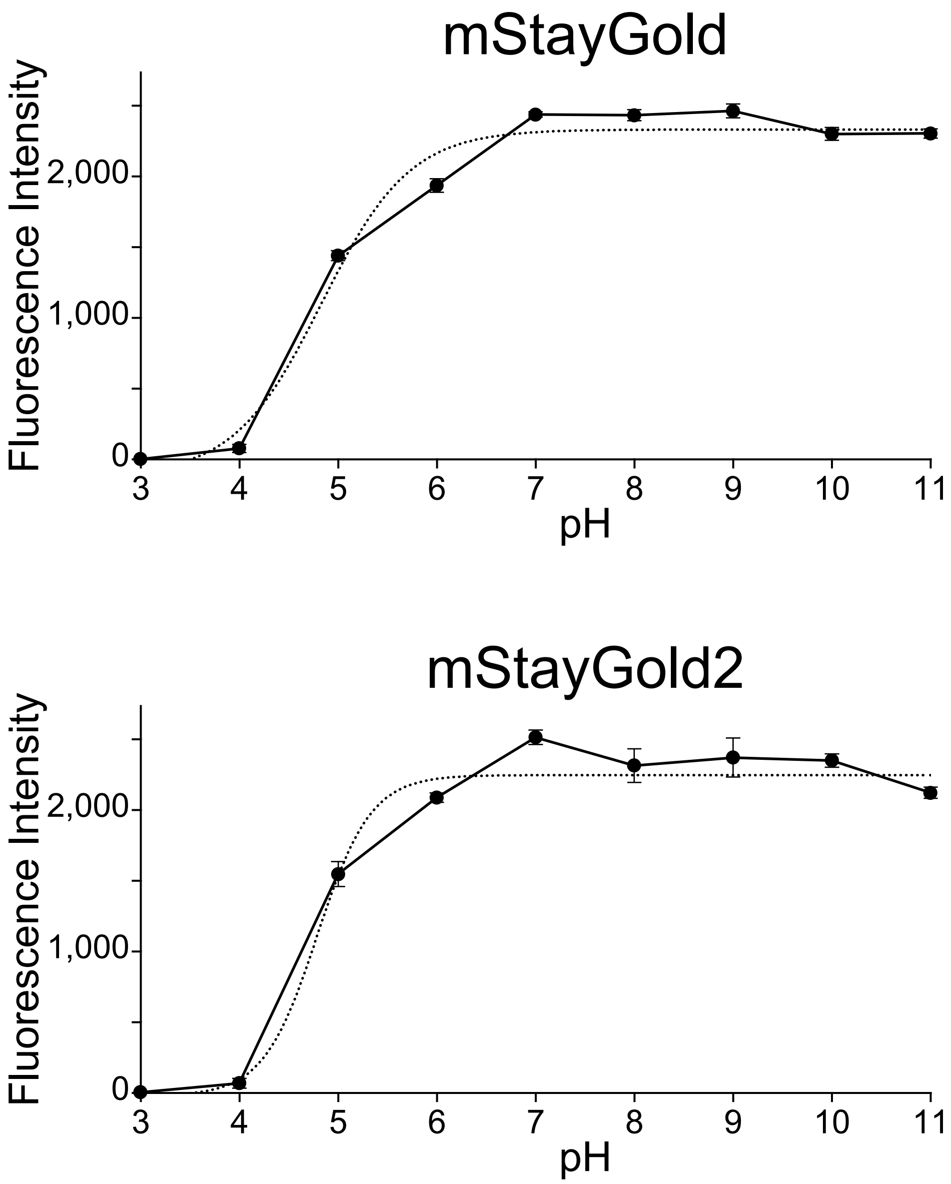

Supplementary Fig. 11 | pH sensitivity of mStayGold and mStayGold2.
The fluorescence intensity was plotted against pH and fitted using the Boltzmann sigmoid function. Data points are shown as means ± s.d. (n = 3 different experiments).

Supplementary Fig. 12

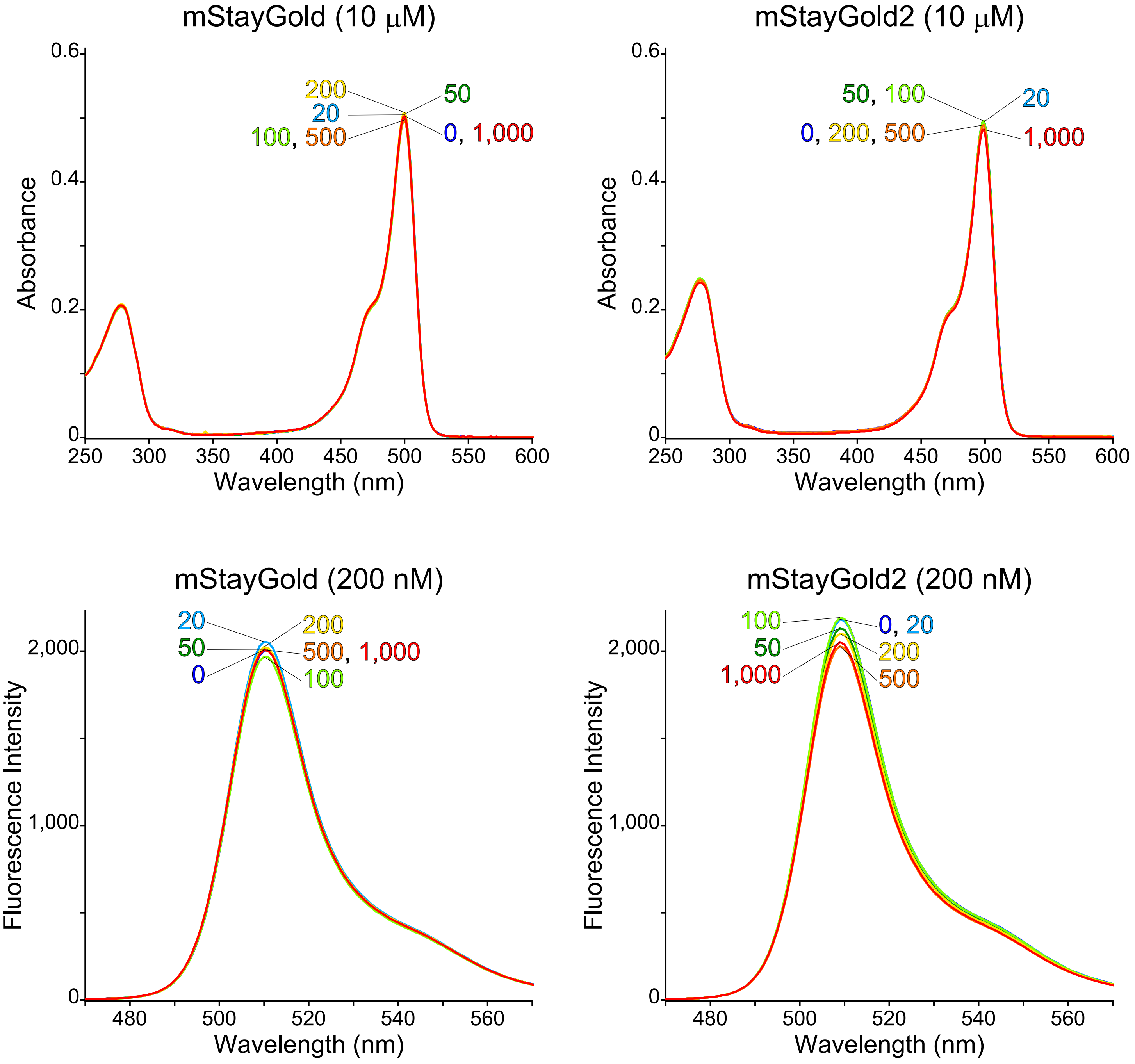

Supplementary Fig. 12 | Resistance of spectral properties of mStayGold and mStayGold2 to Cl^-. Absorption (top) and fluorescence (bottom) spectra. KCl was added to the indicated concentration at pH 7.4 buffered with 50 mM HEPES/NaOH.

Supplementary Fig. 13

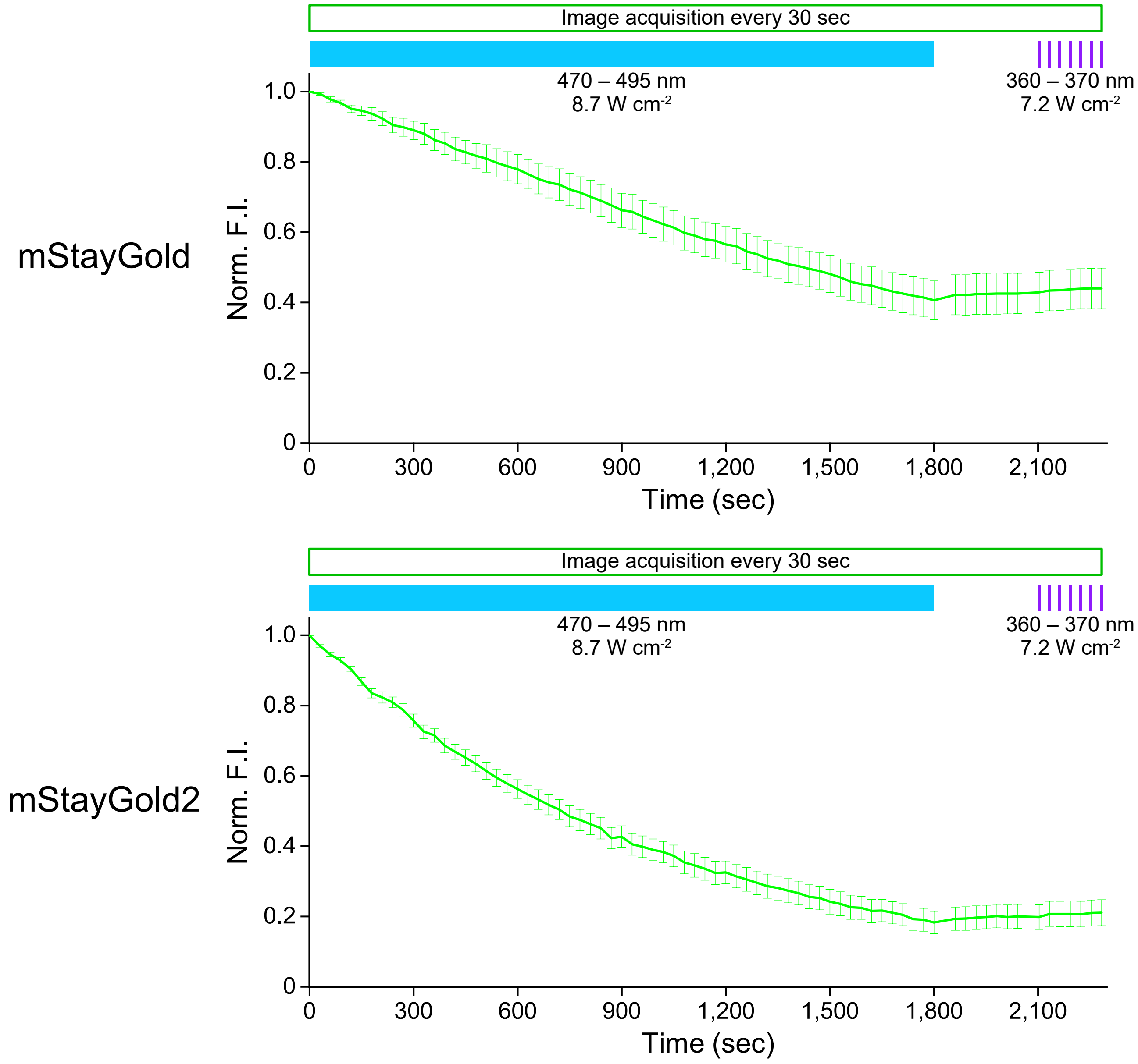

Supplementary Fig. 13 | Irreversibility of photobleaching of mStayGold and mStayGold2. WF photobleaching curves of mStayGold and mStayGold2 that were expressed as fusions to H2B in HeLa cells. Their green fluorescence was monitored every 30 s. Cells were illuminated first continuously through an excitation filter (470–495 nm, 8.7 W cm^{-2}) and then intermittently (pulse duration: 500 ms) through an excitation filter (360–370 nm, 7.6 W cm^{-2}). No substantial recovery was observed. Data points are shown as means ± s.d. (n = 5 cells). Similar results were obtained from 2 other independent experiments for mStayGold and mStayGold2 each.

Supplementary Fig. 14

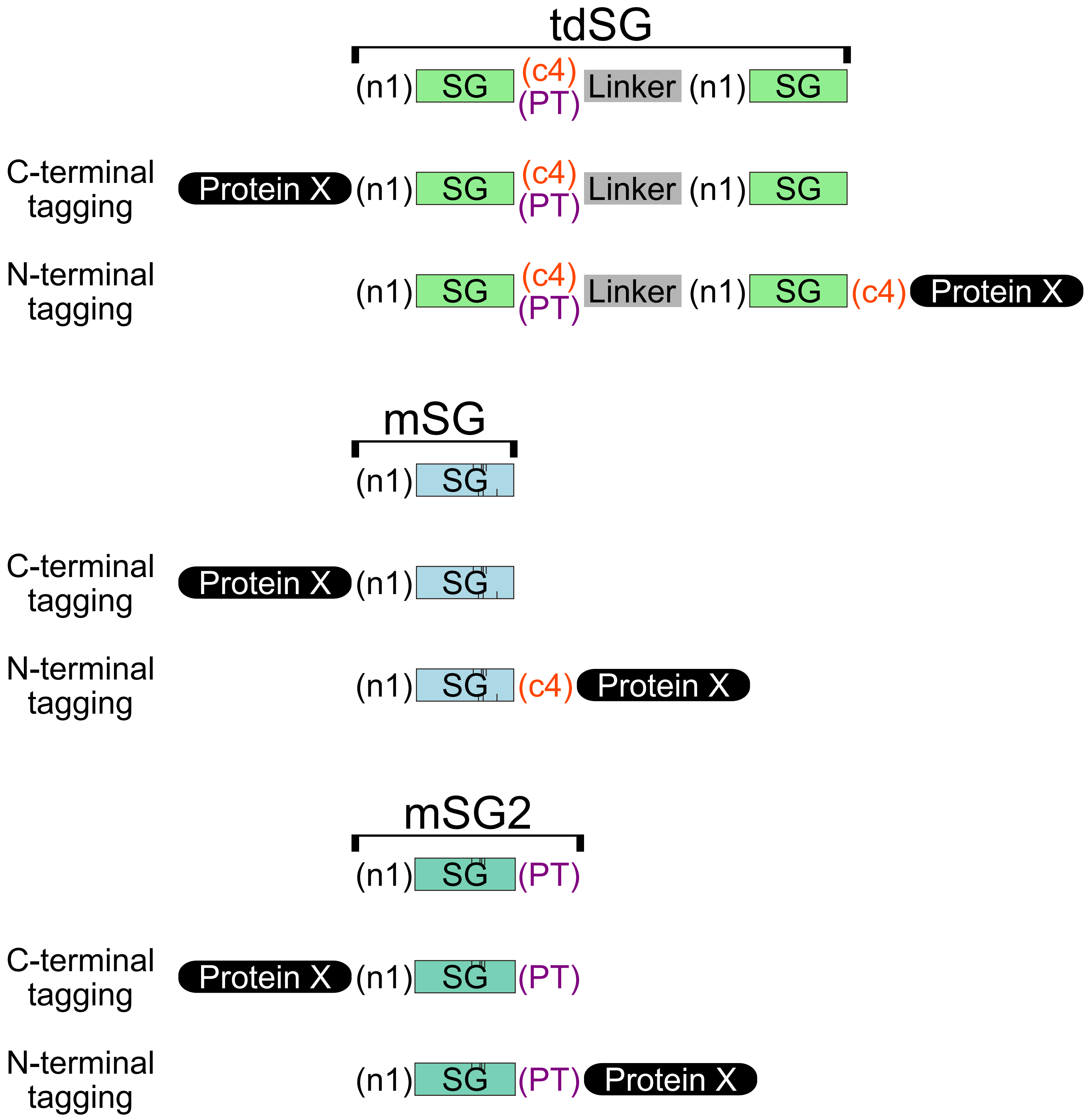

Supplementary Fig. 14 | How to tag a protein of interest (Protein X) with a tandem dimer construct of StayGold (tdSG), mStayGold, and mStayGold2.
StayGold is abbreviated as SG. SG variants, adaptors and linkers are colored in the same manner as in Extended Data Fig. 3.

Supplementary Fig. 15

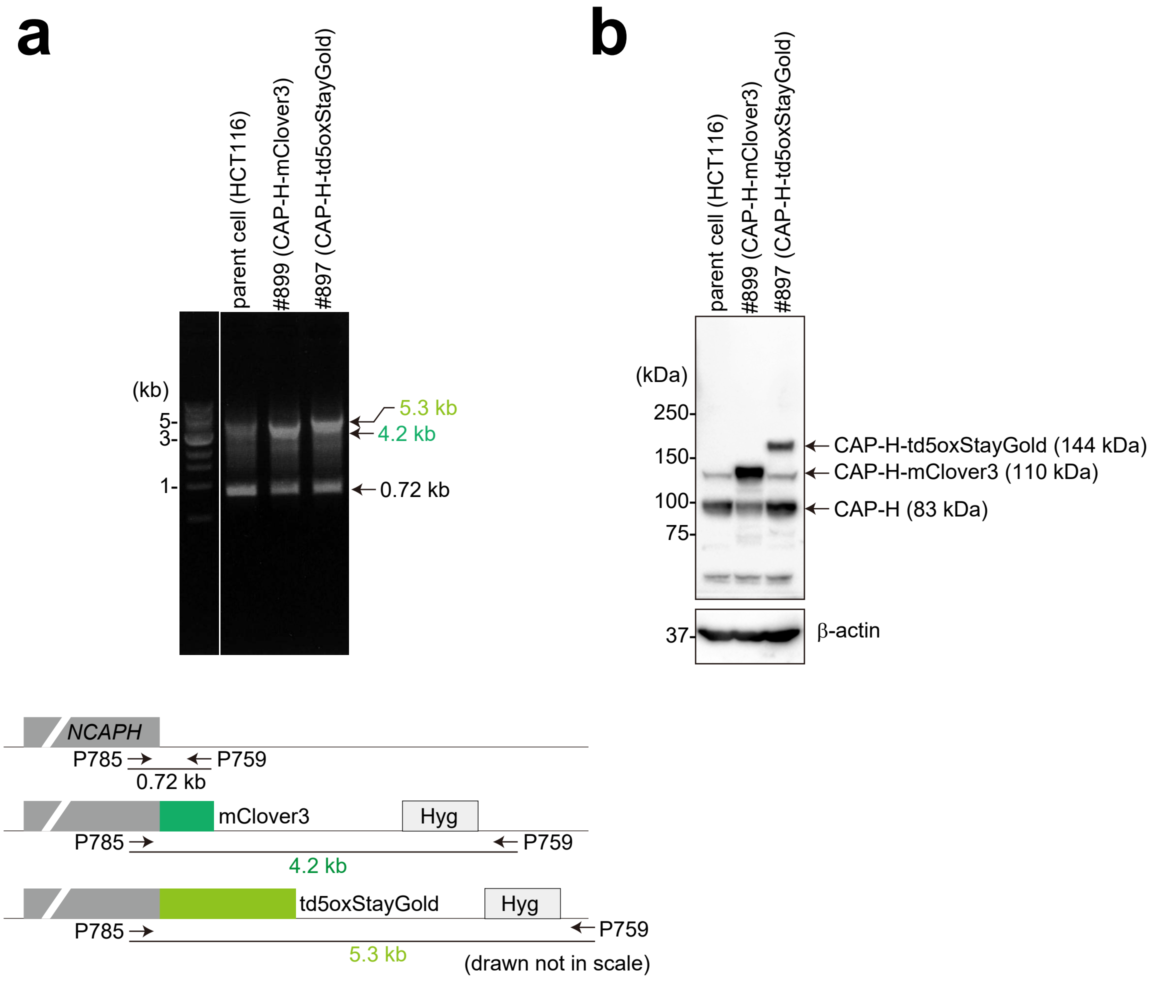

Supplementary Fig. 15 | Validation of knock-in cell lines.
Cell lines #899 and #897 were characterized in parallel with their parent (HCT116) by junction PCR (**a**) and western blotting (**b**). HCT116 cell line is a chromosomally stable human colorectal cancer cell line and has two alleles of the CAP-H gene (*NCAPH*). The following results indicate that the CAP-H gene was heterozygously tagged in both cell lines. The data shown are from a single experiment.
a, Primers located outside of the left (P785) and right (P759) homology arms were used. A 0.72-kb fragment derived from untagged CAP-H was detected for both cell lines #899 and #897 in addition to the fragments from mClover3-CAP-H (4.2 kb) and td5oxStayGold-tagged CAP-H (5.3 kb), respectively. Hyg: hygromycin resistance gene.
b, Anti–CAP-H antibody (Proteintech, #11515-1-AP) was used to characterize the size of CAP-H proteins. An 83-kDa band for untagged CAP-H was detected for both cell lines #899 and #897 in addition to those for mClover3-tagged (110 kDa) and td5oxStayGold-tagged (144 kDa) CAP-H proteins, respectively. Anti–β-actin monoclonal antibody (AC-15) was used as a loading control.

Supplementary Fig. 16

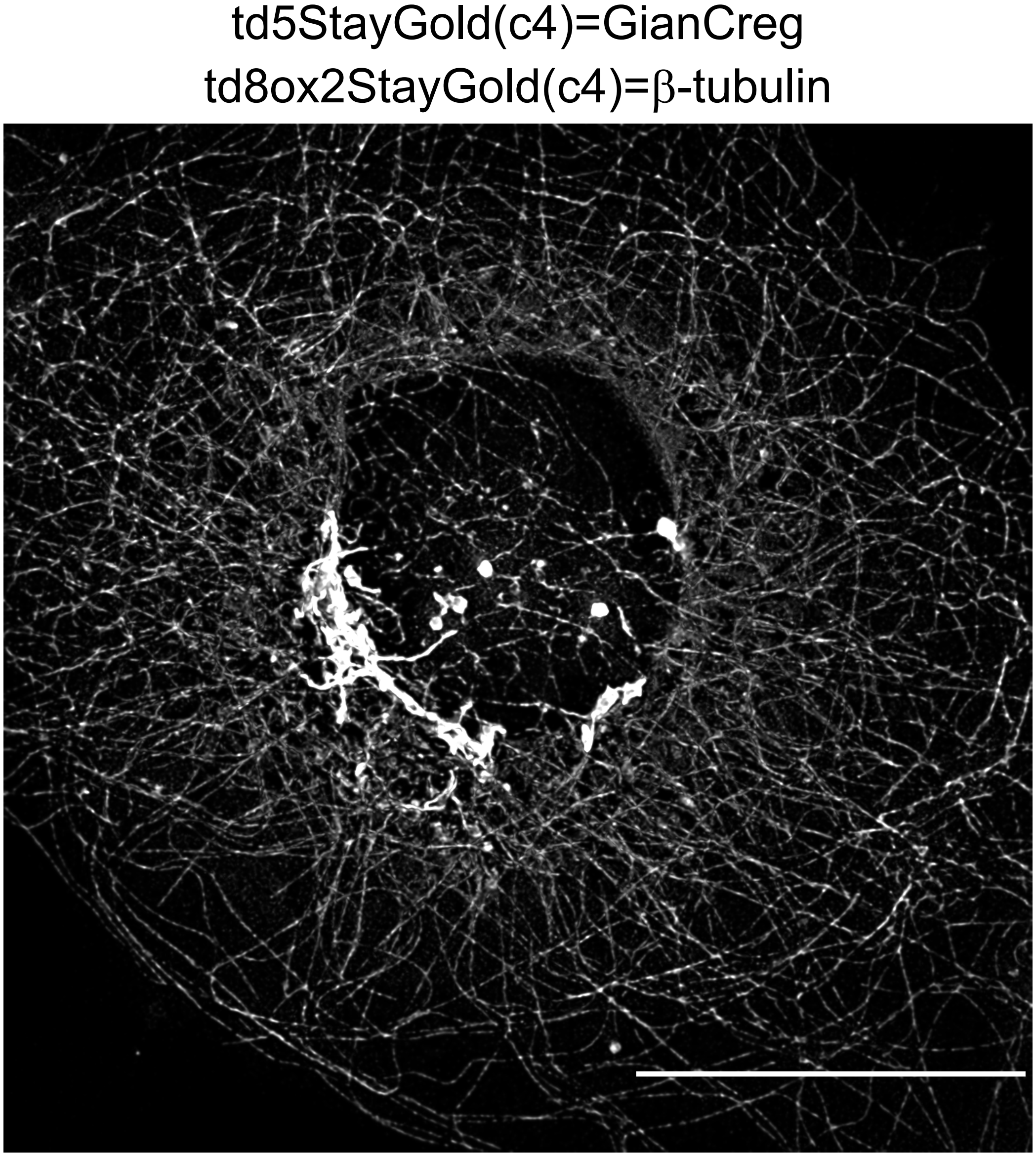

Supplementary Fig. 16 | Dual-targeting of StayGold variants to the Golgi apparatus and microtubule network.
COS-7 cells were cotransfected with td5StayGold(c4)=GianCreg and td8ox2StayGold(c4)=β-tubulin. A fine 3D reconstruction of the whole Golgi apparatus with a microtubule network was generated by volumetric imaging (*z*-step, 0.216 μm; *z*-range, 1.94 μm) using the lattice SIM method (Elyra 7). The Leap mode was used. Scale bar, 20 μm. Shown is a representative of n = 7 independent experiments (transfections).

Supplementary Fig. 17

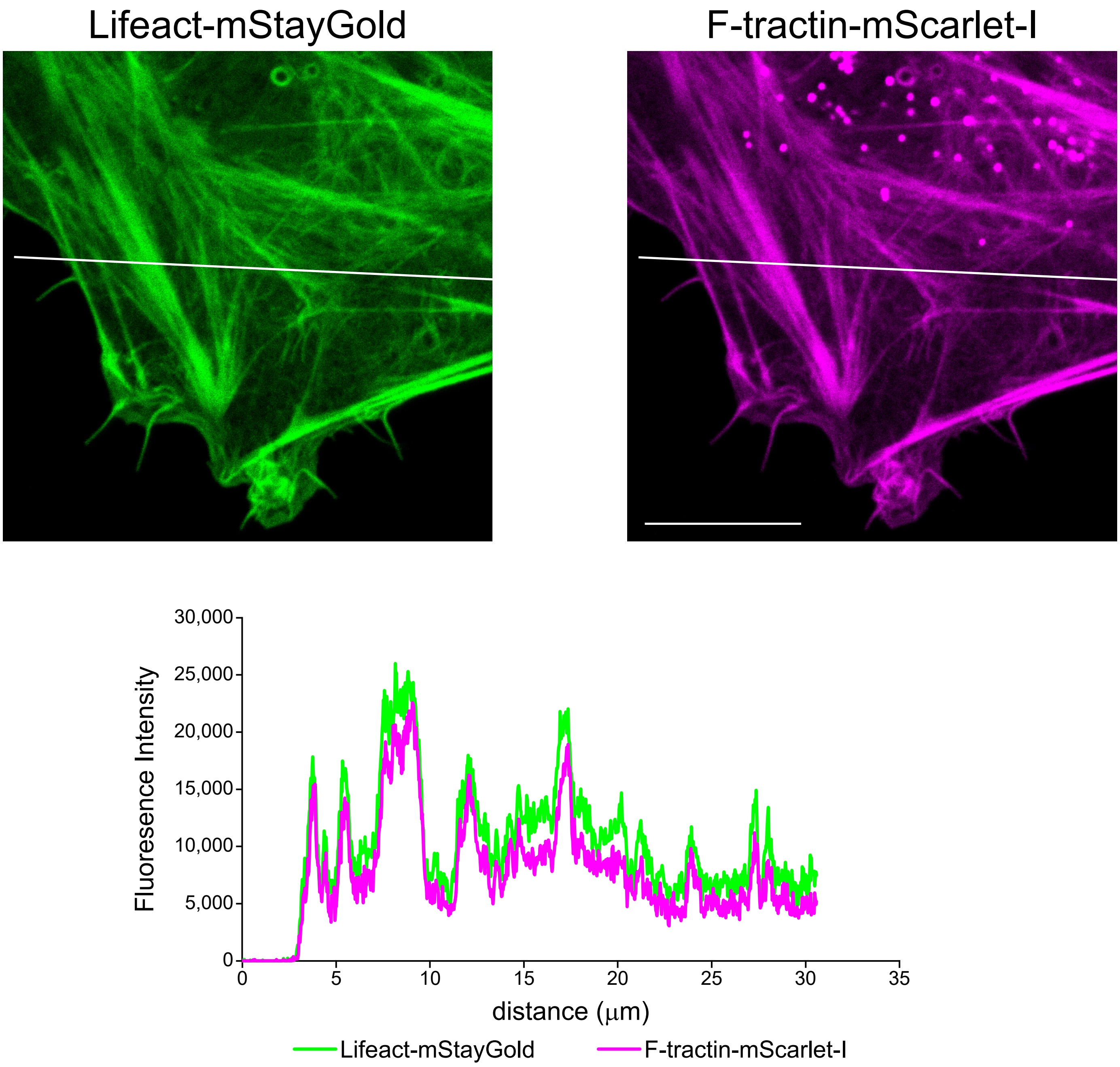

Supplementary Fig. 17 | Colocalization of Lifeact-mStayGold and F-tractin-mScarlet-I in a Vero cell.

top, CLSM (single-beam) images of Lifeact-mStayGold (left, green channel) and F-tractin-mScarlet-I (right, red channel). Scale bar, 10 μm.

bottom, The line profile plots indicate the intensity distribution of green and red channels through the white lines across the cell.

Representative of 10 cells over 3 independent transfections.

Supplementary Fig. 18

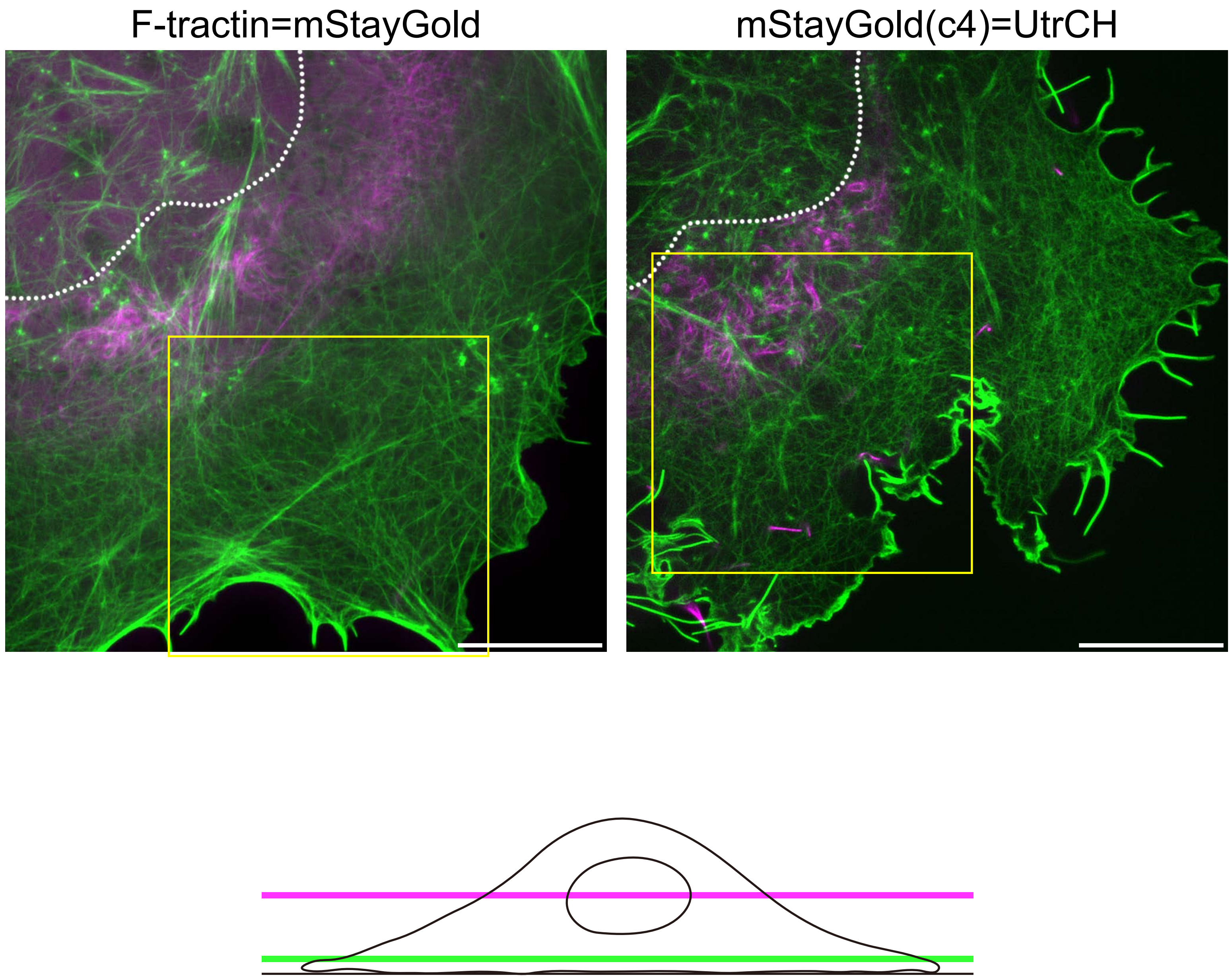

Supplementary Fig. 18 | F-actin distribution visualized by mStayGold-tagged actin-binding domains.
COS-7 cells expressing F-tractin=mStayGold (left) or mStayGold(c4)=UtrCH (right) were imaged by SDSRM (SpinSR10). UtrCH: utrophin calponin homology domain. For each experiment, a confocal image sectioned nearest the cell bottom is shown in green. Images were acquired continuously at 3.18 frames/s for 5.24 min (boxed regions, Supplementary Video 6). A confocal image sectioned across the center of the nucleus is shown in magenta. The nucleus is delineated by a dotted line. Scale bars, 10 μm. The F-tractin=mSayGold images shown are representative of *n* = 15 independent cell samples. The mStayGold(c4)=UtrCH images shown are representative of *n* = 13 independent cell samples.

Supplementary Fig. 19

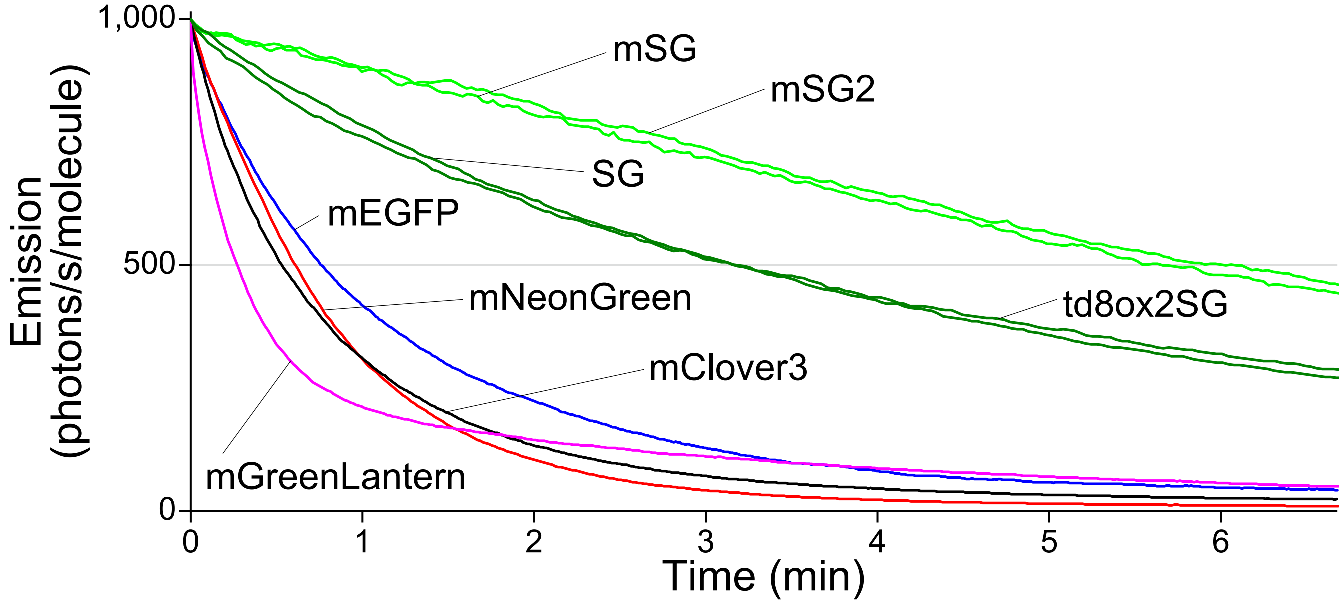

Supplementary Fig. 19 | Photostability of mStayGold, mStayGold2, StayGold, td8ox2StayGold, mEGFP, mClover3, mNeonGreen, and mGreenLantern in live cells with single-beam LSCM. FPs were expressed as fusions to histone 2B (H2B) in HeLa cells. Irradiance: 0.569–0.695 W cm-2. Photobleaching curves are calculated based on the irradiance and FP molecular brightness at 488 nm, plotted as intensity versus normalized total exposure time with an initial emission rate of 1,000 photons/s/molecule. StayGold is abbreviated as SG. Curves are colored as follows. mSG and mSG2, green; SG and td8ox2SG, dark green; mEGFP, blue; mNeonGreen, red; mClover3, black; mGreenLantern, magenta. Data points are shown as means (n = 3 experiments).
The photobleaching half-lives from the initial emission rate of 1,000 photons/s/molecule down to 500 ($t_{1/2}$) were 341 ± 27 s for mSG, 360 ± 19 s for mSG2, 192 ± 34 s for SG, 192 ± 16 s for td8ox2SG, 47 ± 9 s for mEGFP, 32 ± 2 s for mClover3, 36 ± 2 s for mNeonGreen, and 16 ± 0.7 s for mGreenLantern. Values are means ± s.d. (n = 3 independent experiments).

Supplementary Fig. 20

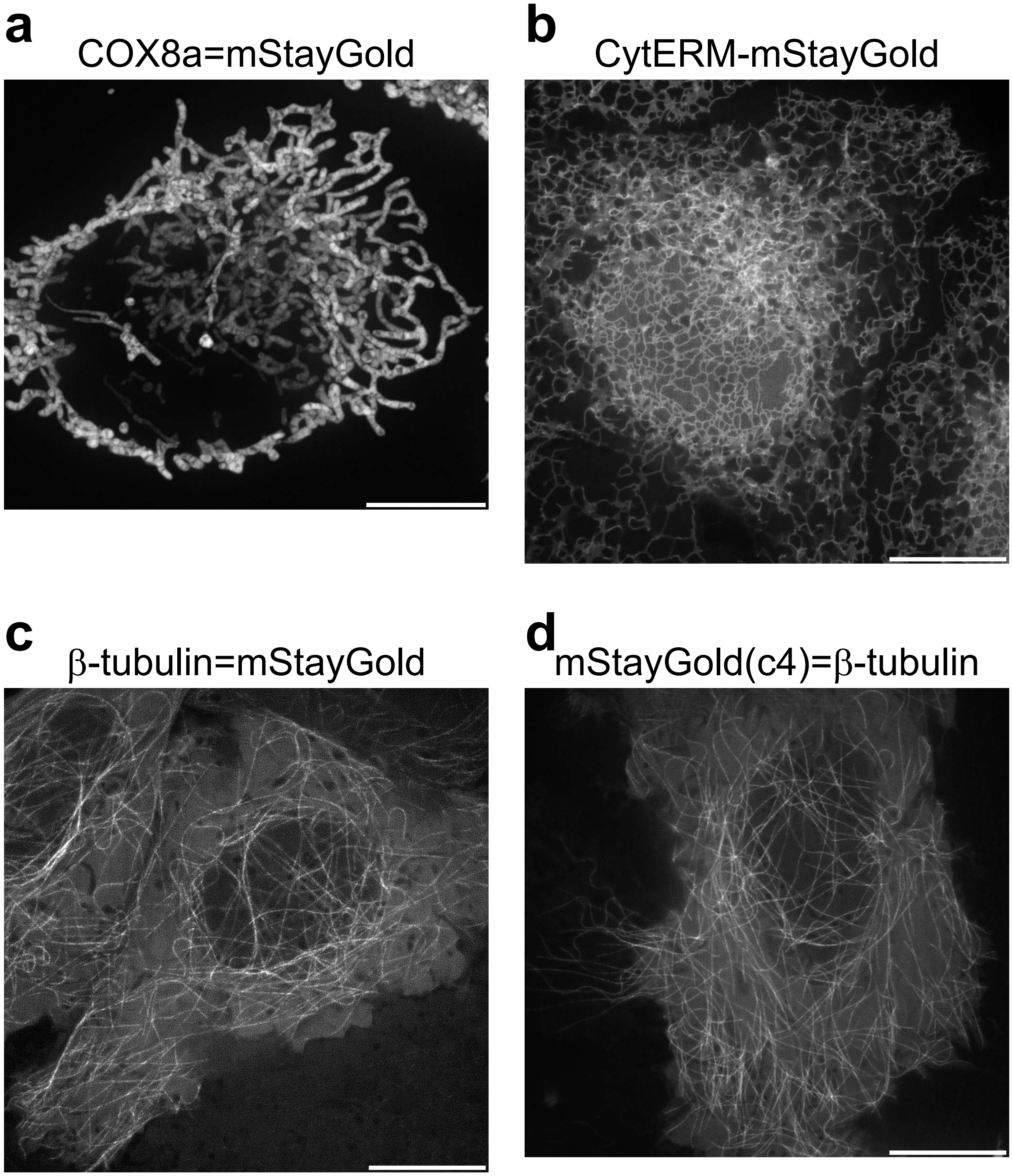

Supplementary Fig. 20 | Examples of molecular fusion and membrane targeting applications of mStayGold.

a, A cell-wide 3D reconstruction of the inner mitochondrial membrane. HeLa cells constitutively expressing COX8a=mStayGold were volume (*z* step, 0.25 μm; *z* range, 9.0 μm) imaged by SpinSR10. An MIP image is shown. Shown is a representative of $n = 4$ cell samples.

b-d, Confocal images highlighting the ER (**b**) and microtubule (**c, d**) networks. HeLa cells transfected with cDNAs of CytERM-mStayGold (**b**), β-tubulin=mStayGold (**c**), and mStayGold(c4)=β-tubulin (**d**) were imaged by SDSRM (SpinSR10). Shown is a representative of $n = 4$ cell samples for each transfection. Scale bars, 10 μm.

Supplementary Table 1

	StayGold pH8.5	StayGold pH5.6
**Wavelength (Å)**	1.0	1.0
**Resolution range (Å)**	91.2 - 1.56 (1.62 - 1.56)	67.0 - 2.2 (2.28 - 2.2)
**Space group**	P 2_1	P 6_1
**Unit cell**	53.9 Å 44.9 Å 93.8 Å 90° 103.4° 90°	134.0 Å 134.0 Å 59.0 Å 90° 90° 120°
**Total reflections**	335808 (28363)	325103 (19041)
**Unique reflections**	61988 (5842)	30868 (3015)
**Multiplicity**	5.4 (4.9)	10.5 (6.3)
**Completeness (%)**	99.2 (93.0)	99.8 (98.1)
**Mean I/sigma(I)**	8.85 (0.46)	10.97 (2.77)
**Wilson B-factor**	17.48	14.25
**R-merge**	0.06797 (0.4089)	0.1706 (0.4423)
**R-meas**	0.07498 (0.4577)	0.1794 (0.4842)
**R-pim**	0.03111 (0.2015)	0.05485 (0.1913)
**CC1/2**	0.999 (0.91)	0.994 (0.85)
**CC***	1.000 (0.976)	0.998 (0.958)
**Reflections used in refinement**	61893 (5759)	30858 (3015)
**Reflections used for R-free**	1986 (197)	1994 (192)
**R-work**	0.1863 (0.3585)	0.1547 (0.2039)
**R-free**	0.2153 (0.3804)	0.1897 (0.2869)
**CC(work)**	0.967 (0.869)	0.968 (0.888)
**CC(free)**	0.956 (0.824)	0.951 (0.833)
**Number of non-hydrogen atoms**	4160	4043
**macromolecules**	3475	3488
**ligands**	40	63
**solvent**	663	492
**Protein residues**	424	425
**RMS(bonds)**	0.002	0.002
**RMS(angles)**	0.54	0.62
**Ramachandran favored (%)**	98.55	97.12
**Ramachandran allowed (%)**	1.45	2.88
**Ramachandran outliers (%)**	0.00	0.00
**Rotamer outliers (%)**	0.54	1.05
**Clashscore**	2.99	2.77
**Average B-factor (Å^2)**	22.63	15.56
**macromolecules (Å^2)**	19.57	14.09
**ligands (Å^2)**	13.83	13.61
**solvent (Å^2)**	32.87	26.23

Supplementary Table 1 | Data collection and refinement statistics.
Statistics for the highest shell are shown in parentheses.

Supplementary Table 2

	Green/Red (G/R) ratio	Normalized G/R ratio	Correction factor	Cellular brightness
EGFP	0.185 ± 0.005	0.369 ± 0.010	1.706	0.216 ± 0.006
mGreenLantern	0.331 ± 0.016	0.659 ± 0.031	1.301	0.507 ± 0.024
mNeonGreen	0.219 ± 0.019	0.435 ± 0.038	1.374	0.317 ± 0.027
SG	0.503 ± 0.024	1.000 ± 0.048	1	1.000 ± 0.048
td5SG	1.050 ± 0.024	2.088 ± 0.048	1	2.088 ± 0.048
td5oxSG	1.364 ± 0.036	2.714 ± 0.072	1	2.714 ± 0.072
td8oxSG	1.373 ± 0.037	2.732 ± 0.074	1	2.732 ± 0.074
td8ox2SG	1.390 ± 0.035	2.766 ± 0.069	1	2.766 ± 0.069
QC2-6 FIQ (mSG)	0.537 ± 0.015	1.069 ± 0.031	1.130	0.946 ± 0.027
QC2-6(PT) (mSG2)	0.406 ± 0.012	0.809 ± 0.024	1.135	0.712 ± 0.021

Supplementary Table 2 | Determination of cellular brightness.
G/R ratio: The fluorescence of a green-emitting FP was divided by the mCherry fluorescence on each pixel and averaged over regions.
Normalized G/R ratio: The above ratio value was normalized to that of StayGold.
Correction factor: The product of the excitation and emission detection efficiencies of a green-emitting FP in the imaging system (Supplementary Fig. 7) was normalized to that of SG to give a correction factor.
Cellular brightness: The above Normalized G/R ratio was divided by the correction factor to give cellular brightness (Fig. 3b, Table 1).
StayGold is abbreviated as SG.

Supplementary Table 3

	Photobleaching half-time (sec)				
	10 W cm^{-2}	30 W cm^{-2}	100 W cm^{-2}	300 W cm^{-2}	1,000 W cm^{-2}
mEGFP	96 ± 11	28.8 ± 2.0	8.02 ± 0.48	2.33 ± 0.14	0.735 ± 0.005
mNeonGreen	75 ± 7	17.0 ± 0.9	5.32 ± 0.60	1.30 ± 0.07	0.314 ± 0.028
mClover3	20.3 ± 0.8	4.40 ± 0.14	0.946 ± 0.167	0.206 ± 0.007	0.0638 ± 0.0104
mGreenLantern	1.2 ± 0.2	0.30 ± 0.04	0.077 ± 0.010	0.024 ± 0.005	0.0124 ± 0.0023
SG	723 ± 13	110.6 ± 15.0	18.08 ± 1.49	1.88 ± 0.13	0.234 ± 0.037
td8ox2SG	716 ± 17	132.5 ± 32.3	15.10 ± 2.36	1.84 ± 0.24	0.204 ± 0.040
mSG	1,052 ± 32	265.4 ± 12.2	54.84 ± 6.08	12.05 ± 0.87	1.860 ± 0.112
mSG2	559 ± 44	172.3 ± 11.9	36.65 ± 5.63	7.89 ± 0.63	1.530 ± 0.083
	Photostability $t_{1/2}$ (sec)				
	10 W cm^{-2}	30 W cm^{-2}	100 W cm^{-2}	300 W cm^{-2}	1,000 W cm^{-2}
mEGFP	358 ± 40	321 ± 23	298 ± 18	260 ± 15	273 ± 2
mNeonGreen	440 ± 38	300 ± 16	313 ± 35	229 ± 12	185 ± 16
mClover3	95 ± 4	62 ± 2	45 ± 8	29 ± 1	30 ± 5
mGreenLantern	7.0 ± 1.2	5.3 ± 0.7	4.6 ± 0.6	4.3 ± 0.8	8.0 ± 1.4
SG	8,297 ± 155	3,808 ± 515	2,076 ± 171	646 ± 44	268 ± 43
td8ox2SG	8,137 ± 190	4,519 ± 1,103	1,716 ± 268	629 ± 81	232 ± 46
mSG	9,713 ± 298	7,348 ± 337	5,062 ± 561	3,336 ± 240	1,717 ± 103
mSG2	5,459 ± 432	5,049 ± 348	3,581 ± 550	2,313 ± 184	1,495 ± 81

Supplementary Table 3 | Photostability of StayGold, td8ox2StayGold, mStayGold, mStayGold2, mEGFP, mNeonGreen, mGreenLantern under WF illumination with irradiance values of 10, 30, 100, 300, and 1,000 W cm^{-2}.
top, Calculated from intensity-normalized curves.
bottom, Time in seconds to reduce the emission rate from 1,000 to 500 photons s^{-1} molecule^{-1}.
Live HeLa cells expressing FP-H2B fusions were used. See Extended Data Fig. 10. Values are means ± s.d. (n = 3 independent experiments). StayGold is abbreviated as SG.

Supplementary Video captions

Supplementary Video 1 | Visualizing the oxygen-dependent development of fluorescence from colonies of JM109(DE3) cells expressing mSG, mSG2, or SG, which were fully grown under a strict anaerobic condition on the agar plate. See Supplementary Fig. 9b.

Supplementary Video 2 | Visualization of chromosome-targeting of td5oxStayGold-tagged condensin I at low copy number expressed via a genome-editing technique. After release from cell cycle arrest, genome-edited HCT116 cells (#897) were imaged for CAP-H-td5oxStayGold (at 488 nm excitation) and SiR-DNA-labeled chromosomes (at 637 nm excitation) using spinning-disk LSCM (SpinSR10) at the indicated times (hour: min). Every 1 min, 3D scanning was executed with a *z*-step size of 1 μm over an axial range of 13 μm, and the green and far-red fluorescence images were merged. Maximum intensity projection (MIP) images are shown. This movie (6.20 MB) has been generated via considerable compression of the original large-volume video data (1.52 GB). Compression was made using TMPGEnc. See Fig. 4. Shown is a representative of $n = 3$ independent experiments.

Supplementary Video 3 | High-speed visualization of chromosome-targeting of td5oxStayGold-tagged condensin I in genome-edited HCT116 cells. After release from cell cycle arrest, genome-edited HCT116 cells (#897) were imaged at a single *z* position for observing CAP-H-td5oxStayGold (488 nm excitation) and for SiR-DNA-labeled chromosomes (637 nm excitation) by spinning-disk LSCM (SpinSR10) at 1 frame per second. Merged images at the indicated times (min: s). This movie (9.05 MB) has been generated via considerable compression of the original large-volume video data (7.03 GB). Compression was made using FFmpeg. See Extended Data Fig. 6a.

Supplementary Video 4 | Photostability comparison between CAP-H-mClover3 and CAP-H-td5oxStayGold under the same optical conditions. Genome-edited HCT116 cells (#899, left vs. #897, right) during prometaphase were volume (*z* step, 0.25 μm; *z* range, 2.5 μm) imaged by spinning-disk LSCM (SpinSR10) with excitation at 488 nm continuously every 6.9 sec over a total period of 278 sec. MIP images are shown. This movie (1.40 MB) has been generated via considerable compression of the original large-volume video data (12.4 MB). Compression was made using TMPGEnc. Elapsed times (min: s). See Extended Data Fig. 6b.

Supplementary Video 5 | Visualization of td5StayGold-harboring Golgi membranes. Volumetric and continuous imaging of HeLa cells expressing td5StayGold(c4)=GianCreg in two independent experiments (top and bottom). Cells were volume (*z* step, 0.5 μm; *z* range, 2.5 μm) imaged by SDSRM (SpinSR10) continuously with an exposure time of 100 ms without using the *z*-drift compensator (IX3-ZDC2, Evident). MIP images are shown. This movie (9.88 MB) has been generated via considerable compression of the original large-volume video data (122 MB). Compression was made using TMPGEnc. Elapsed times (min: s). See Fig. 5b.

Supplementary Video 6 | Visualization of the Golgi apparatus and microtubule network. A COS-7 cell expressing td5StayGold(c4)=GianCreg and td8ox2StayGold(c4)=β-tubulin was volume (*z* step, 0.5 μm; *z* range, 1.5 μm) imaged by SDSRM (SpinSR10) continuously with an exposure time of 200 ms without using the *z*-drift compensator (IX3-ZDC2, Evident). MIP images are shown. This movie (9.43 MB) has been generated via considerable compression of the original large-volume video data (100 MB). Compression was made using TMPGEnc. Elapsed times (min: s).

Supplementary Video 7 | Visualizing F-actin dynamics by continuous, sustainable, cell-wide imaging. COS-7 cells expressing F-tractin=mStayGold (left) or mStayGold(c4)=UtrCH by imaged by SDSRM (SpinSR10) at a single *z* position at 3.18 frames/s for 5.24 min. Elapsed times (min: s). This movie (9.05 MB) has been generated via considerable compression of the original large-volume video data (7.03 GB). Compression was made using TMPGEnc. See Supplementary Fig. 18.

Supplementary Video 8 | Visualizing the effects of drugs on F-actin organization. COS7 cells expressing F-tractin=mStayGold (top) or mStayGld(c4)=UtrCH (bottom) were imaged by SDSRM (SpinSR10) at a single z position at 2.41 frames/s for 13.82 min. Cells were treated with 1 μM Cytochalasin D (left) or 2 μM Latrunculin A (right). Elapsed times (min: s). This movie (9.38 MB) has been generated via considerable compression of the original large-volume video data (4.29 GB). Compression was made using FFmpeg. See Extended Data Fig. 8.

Supplementary Video 9 | Visualizing the inner mitochondrial membrane dynamics by sustainable, cell-wide volumetric SIM imaging. HeLa cells expressing COX8a=mStayGold were 3D scanned continuously with a *z*-step size of 0.11 μm over

an axial range of 2.08 μm by lattice SIM (Elyra 7) at 37 °C. The total number of acquired volumes was 47. SIM^2 was used for image reconstruction. This movie (6.69 MB) has been generated via considerable compression of the original large-volume video data (47 MB). Compression was made using TMPGEnc. Elapsed times (min: s). See Fig. 5c.

Supplementary Video 10 | Visualizing the inner mitochondrial membrane dynamics by fast, sustainable imaging. HeLa cells expressing COX8a=mStayGold were imaged by SDSRM (SpinSR10). Single-plane images were acquired continuously at 8.70 frames/s (exposure time: 100 ms). This movie highlights stable mitochondria. The autofocus function of a *z*-drift compensator (IX3-ZDC2, Evident) was continuously active. The total number of acquired frames was 1,000. This movie (6.71 MB) has been generated via considerable compression of the original large-volume video data (70.7 MB). Compression was made using TMPGEnc. Elapsed times (min: s). The stable IMM dynamics shown is a representative of n = 17 cells over n = 10 independent transfections.

Supplementary Video 11 | Visualizing the inner mitochondrial membrane dynamics by fast, sustainable imaging. HeLa cells expressing COX8a=mStayGold were imaged by SDSRM (SpinSR10). Single-plane images were acquired continuously at 2.41 frames/s (exposure time: 400 ms). This movie highlights a mobile mitochondrium. The autofocus function of a *z*-drift compensator (IX3-ZDC2, Evident) was continuously active. The total number of acquired frames was 500. This movie (3.19 MB) has been generated via considerable compression of the original large-volume video data (31.4 MB). Compression was made using TMPGEnc. Elapsed times (min: s). The mobile IMM dynamics shown is a representative of n = 14 cells over n = 9 independent transfections.

Supplementary Video 12 | Agonist-, antagonist-, and Ca^{2+} ionophore-induced longitudinal changes in IMM structures revealed by fast, sustained, wide imaging. HeLa cells expressing COX8a=mStayGold were imaged by SDSRM (SpinSR10) continuously at a temporal resolution of 2.5 frames per second. Two representative experimental data are shown. Histamine, cyproheptadine, and ionomycin were applied at 1 minute, 2.5 minutes, and 4 minutes, respectively. The autofocus function of a *z*-drift compensator (IX3-ZDC2, Evident) was continuously active. This movie (8.9 MB) has been generated via considerable compression of the original large-volume video data

(412 MB). Compression was made using TMPGEnc. Elapsed times (min: s). See Extended Data Fig. 9. Representatives of *n* = 12 independent experiments (transfections).

Supplementary References

1. Cranfill, P.J. et al. Quantitative assessment of fluorescent proteins. *Nat. Methods* **13,** 557–562 (2016).
2. Campbell, B.C., Petsko, G.A. & Liu, C.F. Crystal structure of green fluorescent protein Clover and design of Clover-based redox sensors. *Structure* **26,** 225–237.e3 (2018).
3. Shaner, N.C. et al. A bright monomeric green fluorescent protein derived from *Branchiostoma lanceolatum*. *Nat. Methods* **10,** 407–409 (2013).
4. Shinoda, H. et al. Acid-tolerant monomeric GFP from *Olindias formosa*. *Cell Chem. Biol.* **25,** 330–338.e7 (2018).
5. Shimozono, S. & Miyawaki, A. Engineering FRET constructs using CFP and YFP. *Methods Cell Biol.* **85,** 381–393 (2008).
6. Shinzawa-Itoh, K. et al. Purification of active respiratory supercomplex from bovine heart mitochondria enables functional studies. *J. Biol. Chem.* **291,** 5178–4184 (2016).
7. Hirano, M. et al. A highly photostable and bright green fluorscent protein. *Nat. Biotechnol.* **40,** 1132–1142 (2022).
8. Jares-Erijman, E. A. & Jovin, T. M. FRET imaging. *Nat. Biotechnol.* **21,** 1387–1395 (2003).
