# Supplementary material for: StayGold variants for molecular fusion and membrane-targeting applications
Source: Nat Methods. 2023 Nov 30;21(4):648–56. doi: 10.1038/s41592-023-02085-6 (PMC11009113; doi:10.1038/s41592-023-02085-6)
Supplement: Supplementary file 2 — Reporting Summary [file 41592_2023_2085_MOESM2_ESM.pdf]

nature portfolio

Corresponding author(s): Atsushi Miyawaki
Last updated by author(s): Oct 1, 2023

Reporting Summary

Nature Portfolio wishes to improve the reproducibility of the work that we publish. This form provides structure for consistency and transparency in reporting. For further information on Nature Portfolio policies, see our Editorial Policies and the Editorial Policy Checklist.

Statistics

For all statistical analyses, confirm that the following items are present in the figure legend, table legend, main text, or Methods section.

n/a	Confirmed	
☐	☒	The exact sample size (*n*) for each experimental group/condition, given as a discrete number and unit of measurement
☐	☒	A statement on whether measurements were taken from distinct samples or whether the same sample was measured repeatedly
☒	☐	The statistical test(s) used AND whether they are one- or two-sided<br>*Only common tests should be described solely by name; describe more complex techniques in the Methods section.*
☒	☐	A description of all covariates tested
☒	☐	A description of any assumptions or corrections, such as tests of normality and adjustment for multiple comparisons
☐	☒	A full description of the statistical parameters including central tendency (e.g. means) or other basic estimates (e.g. regression coefficient) AND variation (e.g. standard deviation) or associated estimates of uncertainty (e.g. confidence intervals)
☒	☐	For null hypothesis testing, the test statistic (e.g. *F*, *t*, *r*) with confidence intervals, effect sizes, degrees of freedom and *P* value noted<br>*Give P values as exact values whenever suitable.*
☒	☐	For Bayesian analysis, information on the choice of priors and Markov chain Monte Carlo settings
☒	☐	For hierarchical and complex designs, identification of the appropriate level for tests and full reporting of outcomes
☐	☒	Estimates of effect sizes (e.g. Cohen's *d*, Pearson's *r*), indicating how they were calculated

Our web collection on statistics for biologists contains articles on many of the points above.

Software and code

Policy information about availability of computer code

Data collection	cellSens (ver.3.1.1 and ver.3.2, EVIDENT), Elyra 7 (ZEN 3.0 SR FP2 (black) ver. 16.0.10.306, ZEISS), FV3000 (ver.2.4.1.198, EVIDENT), Beamline Scheduling Software (BSS) (ver. 2, RIKEN SPring-8 / SPring-8/JASRI), Fusion Solo.7S (Fusion Capt v17.02, Vilber Bio Imaging), Metamorph (ver. 7.1, Molecular Devices), Incucyte (ver. 2022B, SARTORIUS) and LAS X (ver. 3.5.5.19976, Leica Microsystems)
Data analysis	Excel (365, 2019), R (ver. 4.0.3), cellSens (ver.3.1.1 and ver. 3.2), ImageJ (ver. 1.52a and ver. 1.53t), DIALS 2.2.5-g89235367c-release, phenix.phaser (ver. 2.8.3), phenix.refine (ver. 1.20.1), Coot (ver. 8.0.005), CCP4 (ver. 8.0), PyMOL (ver. 2.5.4), ChimeraX (ver. 1.2), TMPGEncPlus (ver. 2.5), FFmpeg (n5.1-5-gaba74d7843-20220802), ZEN (ver. 9.1, 2014), Adobe Photoshop (ver.22.5.8 and CS5 ver. 12.1), LAS X FLIM/FCS (ver. 3.5.5, Leica Microsystems) and Origin Pro(2021b)<br>A customized program was generated based on C++ and OpenCV 3.4.1 (https://opencv.org) for mitochondria dynamics. The code of the program is included in the same R2DMS repository as the raw data (see the Data section below).

For manuscripts utilizing custom algorithms or software that are central to the research but not yet described in published literature, software must be made available to editors and reviewers. We strongly encourage code deposition in a community repository (e.g. GitHub). See the Nature Portfolio guidelines for submitting code & software for further information.

Data

Policy information about availability of data

All manuscripts must include a data availability statement. This statement should provide the following information, where applicable:

- Accession codes, unique identifiers, or web links for publicly available datasets
- A description of any restrictions on data availability
- For clinical datasets or third party data, please ensure that the statement adheres to our policy

The accession numbers in the DDBJ/GenBank databases are LC756333 for mStayGold (QC2-6 FIQ), LC756334 for mStayGold2 (QC2-6(PT)), LC756335 for td5StayGold, LC756336 for td5oxStayGold, and LC756337 for td8ox2StayGold.
The entry IDs in the Protein Data Bank are 8ILK and 8ILL for atomic structures of StayGold crystallized at pH 8.5 and pH 5.6, respectively.
All data generated in this study are available through the RIKEN Research Data & copyrighted-work Management System (R2DMS) (https://dmsgrdm.riken.jp/egdq4/). They are associated with the following items:
Figures 2–5
Extended Data Figs. 1, 2, 5–10
Supplementary Figs. 1, 2, 4–6, 9–13, 15–20
Supplementary Videos 1–12

Human research participants

Policy information about studies involving human research participants and Sex and Gender in Research.

Reporting on sex and gender	This study did not involve human research participants.
Population characteristics	This study did not involve human research participants.
Recruitment	This study did not involve human research participants.
Ethics oversight	This study did not involve human research participants.

Note that full information on the approval of the study protocol must also be provided in the manuscript.

Field-specific reporting

Please select the one below that is the best fit for your research. If you are not sure, read the appropriate sections before making your selection.

☒ Life sciences ☐ Behavioural & social sciences ☐ Ecological, evolutionary & environmental sciences

For a reference copy of the document with all sections, see nature.com/documents/nr-reporting-summary-flat.pdf

Life sciences study design

All studies must disclose on these points even when the disclosure is negative.

Sample size	No explicit calculations were made to determine sample size. We empirically determined the sample size that ensured reproducibility.<br>n = 3 independent WF photobleaching experiments for each construct (Fig. 2, Table 1)<br>n = 4 independent cellular brightness experiments for each construct (Figure 3b, Table 1)<br>n = 3 independent maturation experiments for each construct (Figure 3c)<br>n = 3 independent OSER experiments for each construct (Extended Data Fig. 1)<br>n = 3 independent Fluoppi (PB1-FP) experiments for each construct (Extended Data Fig. 2)<br>n = 3 independent Fluoppi (FP-PB1) experiments for each construct (Extended Data Fig. 5)<br>n = 3 independent colocalization experiments for each Golgi marker (Extended Data Fig. 7)<br>n = 3 independent experiments for photostability at high irradiances for each construct (Extended Data Fig. 10)<br>n = 12 different colonies from 3 independent experiments that examined maturation in E. coli for each construct (Supplementary Fig. 9a)<br>n = 18 different colonies from 3 independent experiments that examined maturation in E. coli for each construct (Supplementary Fig. 9b)<br>n = 3 independent pH dependence measurements for each construct (Supplementary Fig. 11)<br>n = 5 independent cells of one representative experiment that examined photochromism (Supplementary Fig. 13)<br>n = 3 independent experiments for photobleaching under single-beam LSCM for each construct (Supplementary Fig. 19)
Data exclusions	No data were excluded.
Replication	In principle, representative images are shown with "multiple cells per field of view." Exceptions are seen in Extended Data Fig. 6b (top), Extended Data Fig. 7, Extended Data Fig. 8, and Supplementary Figs. 16, 17, 18 and 20.<br><br>The following imaging experiments were independently performed at least twice with similar results. The information can be seen in either

figure legends or Methods section.

<Figure 2, Supplementary Fig. 6>
The curves are representative of three repetitions (n = 3 independent experiments)

<Figure 4>
Shown is a representative of n = 3 independent experiments.

<Figure 5b>
Shown are two representatives of n = 19 independent experiments (transfections) that used SpinSR10 (SDSRM) to image fast-moving tubular structures emerging from the Golgi apparatus.

<Figure 5c>
The cristae dynamics shown is a representative of n = 3 similar observations using Elyra 7 (lattice SIM).

<Extended Data Fig. 8, Supplementary Video 7>
The images and videos shown are representative of
n = 12 independent experiments for cytochalasin D/F-tractin=mStayGold,
n = 7 independent experiments for latrunculin A/F-tractin=mStayGold,
n = 7 independent experiments for cytochalasin D/mStayGold(c4)=UtrCH,
n = 4 independent experiments for latrunculin A/mStayGold(c4)=UtrCH.

<Extended Data Fig. 9, Supplementary Video 12>
Shown are two representatives of n = 12 independent experiments (transfections) that observed histamine- and ionomycin-induced decreases in the mobility of IMM structures.

<Extended Data Fig. 10a>
The curves are representative of three repetitions (n = 3 independent experiments).

<Supplementary Fig. 1a, b>
Shown are representatives of 3 repetitions (n = 3 independent transfections).

<Supplementary Fig. 2>
Representative of n = 3 transfections for OSER and Fluoppi each.

<Supplementary Fig. 4>
Shown is a representative of n = 4 independent experiments that detected different electrophoretic mobility between QC2-6 and QC2-6 FIQ.

<Supplementary Fig. 13>
Shown is a representative of n = 3 independent experiments (transfections) that observed photochromism for mStayGold and mStayGold2 each.

<Supplementary Fig. 15>
The data shown are from a single experiment.

<Supplementary Fig. 16>
Shown is a representative of n = 7 independent experiments (transfections) that imaged rapid motion of both the Golgi apparatus and microtubule network.

<Supplementary Fig. 17>
Shown are representatives of 10 cells over 3 independent transfections.

<Supplementary Fig. 18, Supplementary Video 7>
The F-tractin=mStayGold images shown are representative of n = 15 independent cell samples.
The mStayGold(c4)=UtrCH images shown are representative of n = 13 independent cell samples.

<Supplementary Fig. 20>
Shown is a representative of n = 4 cell samples for each construct.

Randomization	No allocation was performed in this study. All the cell samples observed were randomly selected.
Blinding	Blinding was not done because the data acquisition and analysis were conducted under the identical criteria/conditions/parameters in each comparison.

Reporting for specific materials, systems and methods

We require information from authors about some types of materials, experimental systems and methods used in many studies. Here, indicate whether each material, system or method listed is relevant to your study. If you are not sure if a list item applies to your research, read the appropriate section before selecting a response.

Materials & experimental systems

n/a	Involved in the study	
☐	☒	Antibodies
☐	☒	Eukaryotic cell lines
☒	☐	Palaeontology and archaeology
☒	☐	Animals and other organisms
☒	☐	Clinical data
☒	☐	Dual use research of concern

Methods

n/a	Involved in the study	
☒	☐	ChIP-seq
☒	☐	Flow cytometry
☒	☐	MRI-based neuroimaging

Antibodies

Antibodies used

• Rabbit anti-GM130 Ab (MBL, cat. no. : PM061, Lot no.: 004)
• Rabbit anti-Giantin Ab (PROTEINTECH, cat. no.: 22270-1-AP, Lot no.: 00016709)
• Mouse anti-TGN46 Ab (Sigma-Aldrich, cat. no.: SAB4200355, clone name: TGN46-8)
• Rabbit anti-CAP-H antibody (Proteintech, cat. No.: #11515-1-AP, Lot no.: 00002069)
• Mouse anti-β-actin antibody (Sigma-Aldrich, cat. no.: A1978, clone name: AC-15)
• Donkey anti–rabbit IgG (H+L) highly cross-adsorbed secondary Ab, Alexa Fluor 647–conjugated (Thermo Fisher, cat. no.: A-31573, Lot no.: 2181018)
• Donkey anti–mouse IgG (H+L) highly cross-adsorbed secondary Ab, Alexa Fluor 647–conjugated (Thermo Fisher, cat. no.: A-31571, Lot no.: 2136787)

Validation

• anti-GM130 Ab (MBL, PM061): validated by the manufacturer based on western blotting, and immunofluorescence data. Relevant citations include Nat. Commun. 10(1): 603 (2019), and JBC 292(10): 4089–4098 (2017)
• anti-Giantin Ab (PROTEINTECH, 22270-1-AP): validated by the manufacturer based on western blotting, immunoprecipitation, and immunofluorescence data. Relevant citations include iScience 23(3): 100952 (2020), and J Alzheimers Dis. 85(2): 863–876 (2022)
• anti-TGN46 Ab (Sigma-Aldrich, SAB4200355): validated by the manufacturer based on western blotting, and immunofluorescence data. A Relevant citation includes PLoS pathogens. 18(7): e1010629 (2022)
• anti-CAP-H antibody (Proteintech, 11515-1-AP):validated by the manufacturer based on western blotting, immunoprecipitation, immunofluorescence, ELISA, and immunohistochemistry data. Relevant citations include J Cell Sci 131(6):jcs212092 (2018), and Sci. Rep.12(1):9578 (2022)
• anti-β-actin antibody (Sigma-Aldrich, A1978):validated by the manufacturer based on western blotting data. Relevant citations include PLoS Genet. 15(7):e1008266 (2019), and Mol Cell Biol. 27(8):3165-75 (2007)

Eukaryotic cell lines

Policy information about cell lines and Sex and Gender in Research

Cell line source(s)	HeLa.S3 cells were obtained from ATCC (CCL-2.2).<br>COS-7 cells were obtained from ATCC (CRL-1651).<br>HCT116 cells (CCL-247) were obtained from Masato T. Kanemaki (National Institute of Genetics, Japan).<br>Vero cells were obtained from ATCC (CCL-81).
Authentication	The HeLa.S3 cell line was authenticated by STR profiling. The other cell lines were not authenticated.
Mycoplasma contamination	The HeLa.S3 and COS-7 cell lines were shown to be free from Mycoplasma contamination. The other cell lines were not tested.
Commonly misidentified lines<br>(See ICLAC register)	No commonly misidentified cell lines were used in this study.
